# Supplementary material for: A consensus privacy metrics framework for synthetic data
Source: Patterns (N Y). 2025 Jul 29;6(10):101320. doi: 10.1016/j.patter.2025.101320 (PMC12546437; doi:10.1016/j.patter.2025.101320)
Supplement: Document S1. Supplemental methods and notes and Tables S1–S17 [file mmc1.pdf]

**Patterns, Volume *6***

## Supplemental information

# A consensus privacy metrics framework for synthetic data

**Lisa Pilgram, Fida Kamal Dankar, Jörg Drechsler, Mark Elliot, Josep Domingo-Ferrer, Paul Francis, Murat Kantarcioglu, Linglong Kong, Bradley Malin, Krishnamurty Muralidhar, Puja Myles, Fabian Prasser, Jean Louis Raisaro, Chao Yan, and Khaled El Emam**

# Supplemental Methods and Notes

## Evolution of Statements, Explanations and Qualitative Analysis

The initial question for the consensus process was formulated as follows "How should we evaluate privacy in synthetic data". From this question, our critical analysis (i.e., the report in [1]) was developed which then served to identify relevant statements around privacy metrics in synthetic data.

In the first round, there were 15 statements to score. In the second round, statements were revised to address any ambiguity, two new statements were introduced and four statements omitted based on the panelists' feedback. In the third round, two more statements were omitted so that a total of 11 statements were considered in the final round.

In the scoring rounds, panelists scored statements while having the report (as provided in [1]) as background information. The level of agreement was indicated on a five-point Likert scale and comments could be provided to give explanations for the indicated level of agreement. The scoring rounds were conducted online using the Welphi software. In the online tool, each statement was presented to the panelist with an explanation to clarify the meaning and underlying reasoning.

In the following, we explain the evolution of statements including rephrasing, removal, or introduction of each statement (S1 to S17) and its explanation (E1 to E17). The numbering is meant as unique identifier of the statement. If, for example, S1 is rephrased without changes in meaning (i.e., minor change), a version number is added and it becomes S1.1; if the statement changes its initial meaning (i.e., major change), it is introduced as a new statement with a new identifier. The numbering aligned with the chronological order of the rounds meaning that the numbering becomes discontinuous throughout the study due to the omission of certain statements and the introduction of new ones. If statements or explanations are rephrased, their identifier is highlighted in bold in the round when the change is introduced.

We further present the qualitative analysis from panelists' comments with respect to statements where an agreement score below 4 was indicated in the final round. Note that the qualitative analysis was limited by the panelists' comments, meaning that there were statements (e.g. S16.1) where an agreement level below 4 was indicated but no comment provided. Key findings from this analysis have also been included in the main manuscript where they help to understand the outcomes of the Delphi process.

The panelists' comments were analysed with two objectives: First, to refine the statements and the report in between the rounds and second, to understand the perspective of panelists who indicated an agreement level below 4 in the statements. The comments were coded according to key topics for each statement [2,3]. Key topics were then classified into the following categories:

1. Misunderstandings and ambiguity: Topics that resulted from misunderstandings and reflected ambiguity in the report and/or statement.
2. Misconceptions: Topics that resulted from misconceptions that have been proved wrong in literature and/or through simulations in the report.
3. Counterarguments: Topics that covered remaining counterarguments to the statement.
4. Unrelated: Topics that were unrelated to the disagreement or uncertainty in the statement.

While comments of categories 1 and 2 were used in between the rounds to adjust the statements, category 3 informed the discussion presented in this manuscript.

## Threat Modeling

### The Adversary’s Background Knowledge (S1)

**Table S1. Statement S1**

| | **Round 1** | **Round 2** | **Round 3 (Final Round)** |
|---|---|---|---|
| **Statement** | S1: Disclosure vulnerability metrics should be based on quasi-identifiers rather than on complete records. | S1: Disclosure vulnerability metrics should be based on quasi-identifiers rather than on complete records. | **S1.1**: Disclosure vulnerability metrics should be based on quasi-identifiers. These may vary depending on the data context (e.g., can still be all attributes) and are ascertained by the data controller. |

| | | | |
|---|---|---|---|
| Explanation | E1: Decades of research and practice on identity disclosure in anonymized data, known re-identification attacks, and respective guidelines are based on the assumption that adversary prior knowledge is represented by the quasi-identifiers. Assuming that an adversary knows all of the variables is not necessarily a worst-case assumption for synthetic data. | **E1.1**: Quasi-identifies represent the background knowledge of an adversary. The primary reasons for this recommendation are:<br><br>(a) Decades of research and practice on identity disclosure in anonymized data, known re-identification attacks, and respective guidelines are based on the assumption that adversary prior knowledge is represented by the quasi-identifiers.<br><br>(b) Assuming that an adversary knows all of the variables is not necessarily a worst-case assumption for synthetic data (please see report section 1.5.1 in the report for an explanation of this point). It can be shown that by considering all of the variables we may be underestimating disclosure vulnerability.<br><br>(c) There are generally accepted criteria for deciding what a quasi-identifier is. Quasi-identifiers can vary and are ultimately ascertained by the data controller for a given dataset. | E1.1: Quasi-identifies represent the background knowledge of an adversary. The primary reasons for this recommendation are:<br><br>(a) Decades of research and practice on identity disclosure in anonymized data, known re-identification attacks, and respective guidelines are based on the assumption that adversary prior knowledge is represented by the quasi-identifiers.<br><br>(b) Assuming that an adversary knows all of the variables is not necessarily a worst-case assumption for synthetic data (please see section 1.5.1 in the report for a detailed explanation and justification for this point). It can be shown that by considering all the variables we may be underestimating disclosure vulnerability.<br><br>(c) There are generally accepted criteria for deciding what a quasi-identifier is. Quasi-identifiers can vary and are ultimately ascertained by the data controller for a given dataset. Treating all attributes as quasi-identifiers is itself an assumption. |
| Commentary | NA | The explanation was rephrased to include further details from the report as a rationale for this statement. | The rephrasing of the statement did not change its meaning but incorporated parts of the explanation. |

| | | | |
|---|---|---|---|
| **Score Distribution** | Strongly Disagree: 0/13 (0.0%)<br>Disagree: 3/13 (23.1%)<br>Neutral: 3/13 (23.1%)<br>Agree: 4/13 (30.8%)<br>Strongly Agree: 3/13 (23.1%) | Strongly Disagree: 0/13 (0.0%)<br>Disagree: 3/13 (23.1%)<br>Neutral: 2/13 (15.4%)<br>Agree: 5/13 (38.5%)<br>Strongly Agree: 3/13 (23.1%) | Strongly Disagree: 0/13 (0.0%)<br>**Disagree: 2/13 (15.4%)**<br>**Neutral: 1/13 (7.7%)**<br>Agree: 7/13 (53.8%)<br>Strongly Agree: 3/13 (23.1%) |
| **Qualitative Analysis** | Even though there was stable consensus on agreement for this recommendation, we want to acknowledge that there were 2/13 (15.4%) experts who disagreed with this statement. While our simulation on quasi-identifiers (QIs) in the critical analysis (see [1]) provided compelling evidence against the first argument, subjectivity in defining QIs remained as a key topic of disagreement. For example, the diagnosis of obesity or diabetes mellitus can be considered as a QI since its knowability can be quite high (e.g., public awareness, photos, discussions on social media) while the diagnosis of interstitial nephropathy may not be a familiar term to the patient themselves. Consequently, it can be challenging to label the attribute *diagnosis* as either QI or non-QI across all diagnoses. Also, the context of the data may shift over time and an attribute that was once deemed not to be a QI may then be considered one. This contextual interpretation is very likely to result in inter-individual variability. Another key point was made that any attribute could be a QI depending on the adversary so that all attributes in a record should be considered as QIs. While it may be the most prudent approach to treat all attributes as QIs, this could, in practice, become computationally problematic as a prudent approach would also require considering all potential combinations of those QIs when calculating the metrics. | | |
| **Score Distribution Plot** | 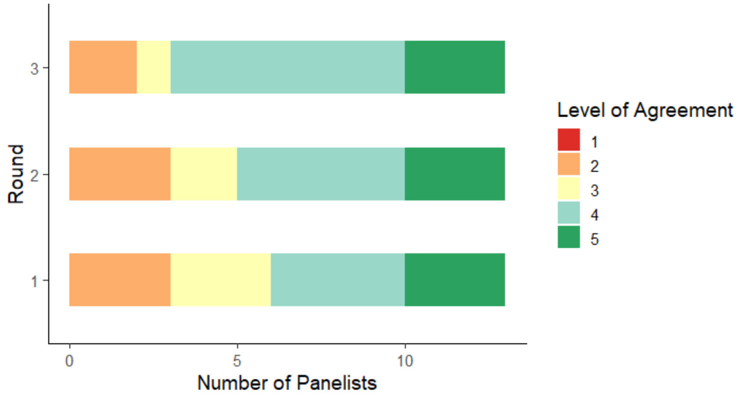 | | |

## Motivations, Constraints and Targets (S2)

**Table S2. Statement S2**

| | **Round 1** | **Round 2** | **Round 3 (Final Round)** |
|---|---|---|---|
| **Statement** | S2: Disclosure vulnerability metrics should not be calculated on a pre-selected subset of “vulnerable” records but for all of the records. | S2: Disclosure vulnerability metrics should not be calculated on a pre-selected subset of “vulnerable” records but for all of the records. | S2: Disclosure vulnerability metrics should not be calculated on a pre-selected subset of “vulnerable” records but for all of the records. |
| **Explanation** | E2: Pre-selection of records mainly involves choosing “vulnerable” records but it remains unclear if these records indeed are the ones with highest disclosure vulnerability or rather vulnerable in a more ethical sense. | **E2.1**: Pre-selection of records mainly involves choosing “vulnerable” records but it remains unclear if these records indeed are the ones with highest disclosure vulnerability or rather vulnerable in a more ethical sense. Knowledge on which records are the ones with highest disclosure vulnerability can only be obtained when calculating the vulnerability for all records in the first place. | E2.1: Pre-selection of records mainly involves choosing “vulnerable” records but it remains unclear if these records indeed are the ones with highest disclosure vulnerability or rather vulnerable in a more ethical sense. Knowledge on which records are the ones with highest disclosure vulnerability can only be obtained when calculating the vulnerability for all records in the first place. |
| **Commentary** | NA | The explanation was rephrased to include further details from the report as a rationale for this statement. | NA |
| **Score Distribution** | Strongly Disagree: 0/13 (0.0%)<br>Disagree: 0/13 (0.0%)<br>Neutral: 3/13 (23.1%)<br>Agree: 3/13 (23.1%)<br>Strongly Agree: 7/13 (53.8%) | Strongly Disagree: 0/13 (0.0%)<br>Disagree: 0/13 (0.0%)<br>Neutral: 2/13 (15.4%)<br>Agree: 2/13 (15.4%)<br>Strongly Agree: 9/13 (69.2%) | Strongly Disagree: 0/13 (0.0%)<br>Disagree: 0/13 (0.0%)<br>Neutral: 2/13 (15.4%)<br>Agree: 2/13 (15.4%)<br>Strongly Agree: 9/13 (69.2%) |

| | |
|---|---|
| **Score Distribution Plot** | 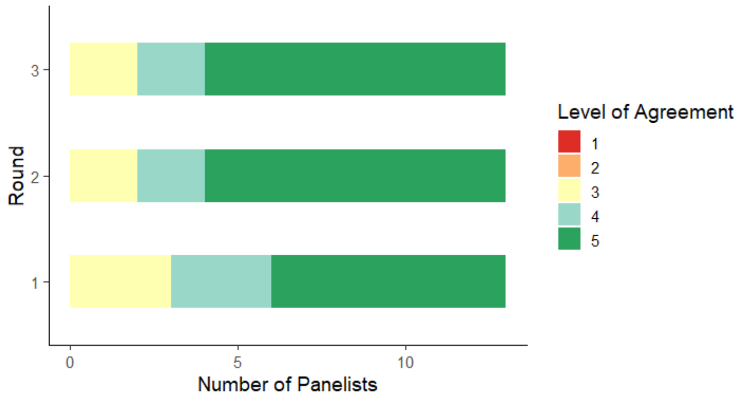  |

# Identity Disclosure

## Record-Level Similarity (S3)

**Table S3. Statement S3**

| | **Round 1** | **Round 2** | **Round 3 (Final Round)** |
|---|---|---|---|
| **Statement** | S3: Similarity metrics that are not part of attribute or membership disclosure should not be used to report privacy in synthetic data. | **S3.1**: Stand-alone similarity metrics (i.e., that are not part of attribute or membership disclosure) should not be used to report privacy in synthetic data. | S3.1: Stand-alone similarity metrics (i.e., that are not part of attribute or membership disclosure) should not be used to report privacy in synthetic data. |
| **Explanation** | E3: Similarity metrics that are not part of calculating membership or attribute disclosure metrics have no precise interpretation. In contrast to anonymized data, matching a synthetic to training record by itself does not translate into an identity disclosure vulnerability as it does not imply learning correct information. Similarity by itself may or may not translate into disclosure vulnerability. This ambiguity when interpreting such metrics makes clear that they are not proper disclosure vulnerability metrics. | **E3.1**: Stand-alone similarity metrics (in fully synthetic data) that are not part of calculating membership or attribute disclosure metrics have no precise interpretation. Our analysis in Section 2.2.6 shows that these metrics do not convey additional information beyond membership and attribute disclosure metrics. | E3.1: Stand-alone similarity metrics (in fully synthetic data) that are not part of calculating membership or attribute disclosure metrics have no precise interpretation. Our analysis in Section 2.2.6 shows that these metrics do not convey additional information beyond membership and attribute disclosure metrics. |
| **Commentary** | NA | The rephrasing in the statement did not change its meaning but avoided ambiguity in the definition of record-level similarity (versus its use as part of other metrics). The explanation was rephrased to include further details from the report as a rationale for this statement. | NA |
| **Score** | Strongly Disagree: 0/13 (0.0%) | Strongly Disagree: 0/13 (0.0%) | Strongly Disagree: 0/13 (0.0%) |

<table>
<tr><td></td><td>Disagree: 0/13 (0.0%)<br>Neutral: 3/13 (23.1%)<br>Agree: 6/13 (46.1%)<br>Strongly Agree: 4/13 (30.8%)</td><td>Disagree: 0/13 (0.0%)<br>Neutral: 2/13 (15.4%)<br>Agree: 6/13 (46.1%)<br>Strongly Agree: 5/13 (38.5%)</td><td>Disagree: 0/13 (0.0%)<br>Neutral: 2/13 (15.4%)<br>Agree: 7/13 (53.8%)<br>Strongly Agree: 4/13 (30.8%)</td></tr>
<tr><td>Score Distribution Plot</td><td colspan="3">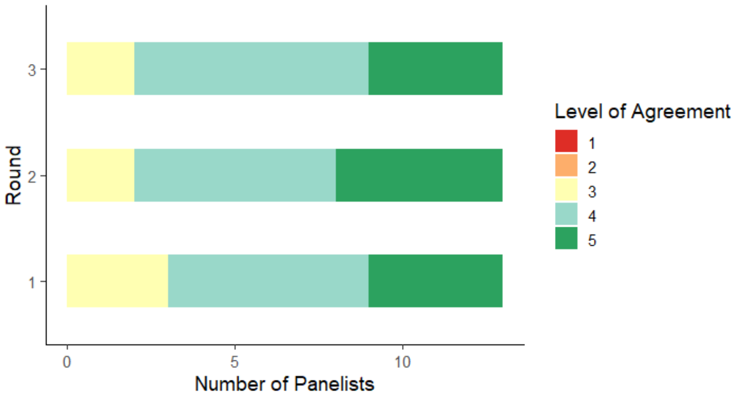
</td></tr>
</table>

## Membership Disclosure

### Assumptions in Narrative and Metric (S4)

**Table S4. Statement S4**

| | **Round 1** | **Round 2** | **Round 3 (Final Round)** |
|---|---|---|---|
| **Statement** | S4: Membership disclosure vulnerability should only be evaluated when the adversary would learn something new for targets drawn from the same population as the training dataset. | **S4.1:** Membership disclosure vulnerability should only be evaluated when the assumptions of the current metrics hold which is that the adversary would learn something new for targets drawn from the same population as the training dataset. | S4.1: Membership disclosure vulnerability should only be evaluated when the assumptions of the current metrics hold which is that the adversary would learn something new for targets drawn from the same population as the training dataset. |
| **Explanation** | E4: Membership disclosure metrics assume that the adversary draws target records from the same population as the training dataset is drawn from. The information they learn from such an attack is membership and in case of interventional studies the information that a certain procedure was applied. A vaccination study is an example where the calculation of membership disclosure vulnerability by current metrics is meaningful. | **E4.1:** Current membership disclosure metrics assume that the adversary draws target records from the same population as the training dataset is drawn from. Most narratives, however, make the assumption that targets are drawn from a different population. While the latter may be a reasonable scenario, it is not reflected in current metrics. Therefore, current metrics should only be applied when the assumption of the metric is in line with the adversarial assumptions, otherwise using the current metrics in the wrong context may give wrong vulnerability estimates. | E4.1: Current membership disclosure metrics assume that the adversary draws target records from the same population as the training dataset is drawn from. Most narratives, however, make the assumption that targets are drawn from a different population. While the latter may be a reasonable scenario, it is not reflected in current metrics. Therefore, current metrics should only be applied when the assumption of the metric is in line with the adversarial assumptions, otherwise using the current metrics in the wrong context may give wrong vulnerability estimates. |
| **Commentar y** | NA | The rephrasing in the statement did not change its meaning but incorporated parts of the explanation. The explanation was rephrased to include further details from | NA |

| | | | |
|---|---|---|---|
| | | the report as a rationale for this statement. | |
| **Score Distribution** | Strongly Disagree: 1/13 (7.7%)<br>Disagree: 2/13 (15.4%)<br>Neutral: 0/13 (0.0%)<br>Agree: 8/13 (61.5%)<br>Strongly Agree: 2/13 (15.4%) | Strongly Disagree: 2/13 (15.4%<br>Disagree: 2/13 (15.4%)<br>Neutral: 0/13 (0.0%)<br>Agree: 8/13 (61.5%)<br>Strongly Agree: 1/13 (7.7%) | Strongly Disagree: 0/13 (0.0%)<br>**Disagree: 1/13 (7.7%)**<br>Neutral: 0/13 (0.0%)<br>Agree: 9/13 (69.2%)<br>Strongly Agree: 3/13 (23.1%) |
| **Qualitative Analysis** | While 12/13 (92.3%) panelists agreed to this statement, there was 1/13 (7.7%) disagreement. Key topics that have been raised as counterarguments are the benefits of measuring membership disclosure in terms of trust, the consideration of membership rather as a flavor of attribute disclosure than a disclosure by itself and the unclear informative value of membership disclosure in practice. The remaining disagreement cannot be clearly attributed to one of these key topics. Given that most common key topics in previous rounds were misunderstandings and ambiguity, it may be a remnant of that. | | |
| **Score Distribution Plot** | 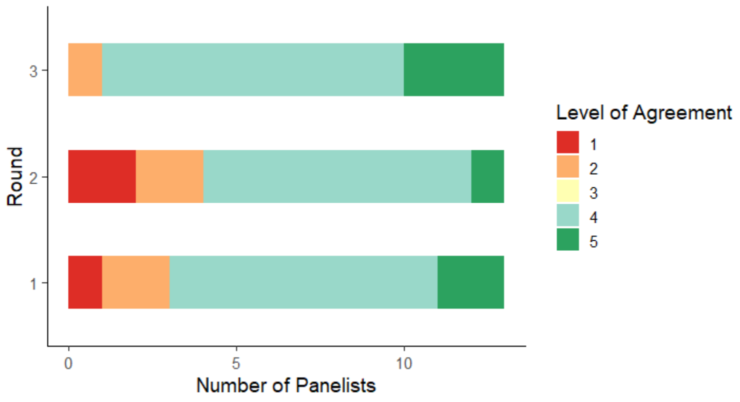 | | |

## Prevalence-Aware Interpretation: A Naïve Membership Guess (S5, S6 & S16)

**Table S5. Statement S5**

| | **Round 1** | **Round 2** | **Round 3 (Final Round)** |
|---|---|---|---|
| **Statement** | S5: Calculating membership disclosure vulnerability is only meaningful when the naïve (i.e., inherent) membership disclosure vulnerability is low. | **S5**: omitted | NA |
| **Explanation** | E5: If the F_naive value is already high then the calculation of an absolute or relative membership disclosure vulnerability of the synthetic data does not provide meaningful guidance in decisions on SDG. The computation does not alter the conclusion that the situation exhibits high membership disclosure and the focus should only be on attribute disclosure. | NA | NA |
| **Commentary** | NA | The statement was removed since it was overly prescriptive and not universally applicable as good practice. | NA |
| **Score Distribution** | Strongly Disagree: 0/13 (0.0%)<br>Disagree: 4/13 (30.8%)<br>Neutral: 1/13 (7.7%)<br>Agree: 6/13 (46.1%)<br>Strongly Agree: 2/13 (15.4%) | NA | NA |

| | |
|---|---|
| **Score Distribution Plot** | 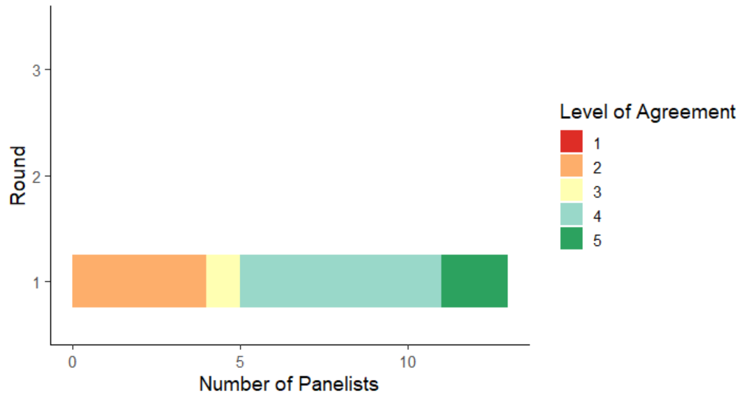 |

**Table S6. Statement S6**

| | **Round 1** | **Round 2** | **Round 3 (Final Round)** |
|---|---|---|---|
| **Statement** | S6: In membership disclosure vulnerability, a relative vulnerability higher than its threshold should only be considered as unacceptably high when the absolute vulnerability is higher than its threshold. | **S6**: omitted | NA |
| **Explanation** | E6: The relative metric cannot be interpreted uniformly across the entire scale and is probably not always informative by itself. | NA | NA |
| **Commentary** | NA | The statement was removed since it does not hold for *F1 score* where S16 accounts for a prevalence-aware interpretation. | NA |
| **Score Distribution** | Strongly Disagree: 1/13 (7.7%)<br>Disagree: 0/13 (0.0%)<br>Neutral: 2/13 (15.4%)<br>Agree: 8/13 (61.5%)<br>Strongly Agree: 2/13 (15.4%) | NA | NA |

| Score Distribution Plot | 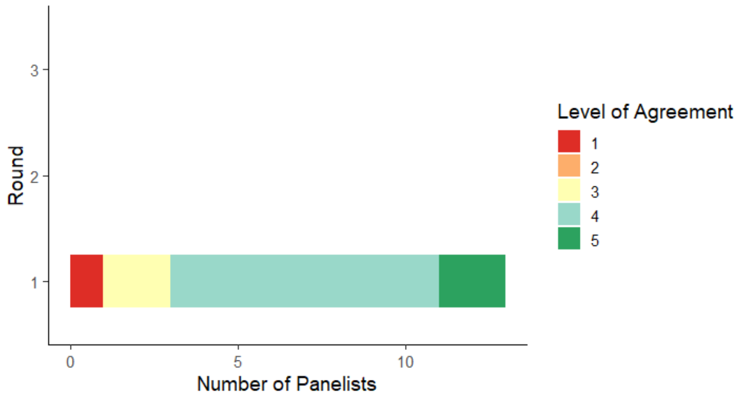 |
| --- | --- |

**Table S7. Statement S16**

| | Round 1 | Round 2 | Round 3 (Final Round) |
|---|---|---|---|
| **Statement** | NA | **S16**: Because the most commonly used membership disclosure metric is an F1 score, and this is prevalence dependent, it needs to be reported relative to an adversary guessing membership. | **S16.1**: Because the F1 score, which is commonly used in membership disclosure metrics, is prevalence dependent, it needs to be reported relative to an adversary guessing membership. |
| **Explanation** | NA | **E16**: It is known that metrics like the F1 score vary with prevalence. The absolute value can therefore not be uniformly interpreted. For example, with a low naïve guess (e.g., 0.01), an F1 score of 0.5 would still be considered as high, reflecting reasonable success given the rarity of members. This naïve baseline can be accounted for by a relative metric (F_rel) or by adjusting threshold according to the baseline. | **E16.1**: It is known that metrics like the F1 score are affected by prevalence. The absolute value can therefore not be uniformly interpreted. For example, with a low naïve guess (e.g., 0.01), an F1 score of 0.5 would still be considered as high, reflecting reasonable success given the rarity of members. This naïve baseline can be accounted for by a relative metric (F_rel) or by adjusting threshold according to the baseline. |
| **Commentary** | NA | The statement was introduced to replace S5 & S6, addressing the same theme with a more applicable recommendation. | The rephrasing in the statement did not change the recommendation but clarified the causal explanation. The explanation was rephrased to include further details from the report as a rationale for this statement. |
| **Score Distribution** | NA | Strongly Disagree: 1/13 (7.7%)<br>Disagree: 0/13 (0.0%)<br>Neutral: 2/13 (15.4%)<br>Agree: 4/13 (30.8%)<br>Strongly Agree: 6/13 (46.1%) | Strongly Disagree: 0/13 (0.0%)<br>Disagree: 0/13 (0.0%)<br>Neutral: 1/13 (7.7%)<br>Agree: 5/13 (38.5%)<br>Strongly Agree: 7/13 (53.8%) |

| | |
|---|---|
| **Score Distribution Plot** | 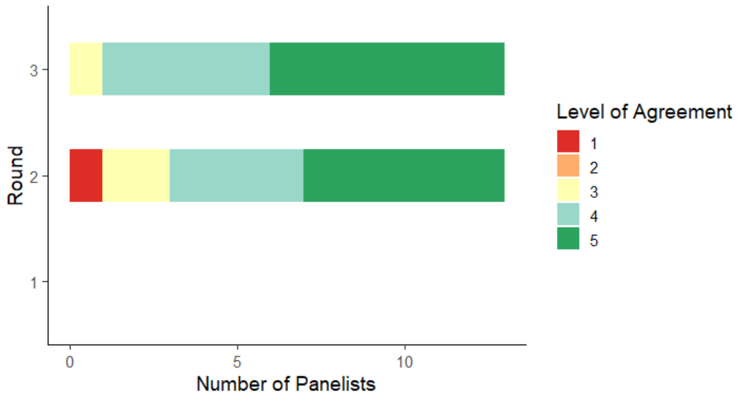 |

## Attribute Disclosure

### The Scope of Attribute Disclosure Vulnerability (S7)

**Table S8. Statement S7**

| | Round 1 | Round 2 | Round 3 (Final Round) |
|---|---|---|---|
| **Statement** | S7: Meaningful attribute disclosure vulnerability only applies to individuals that are in the dataset (i.e., members). Penalizing accurate prediction on individuals that have not been part of the dataset (i.e., group privacy) requires a broader ethical framework. | S7: Meaningful attribute disclosure vulnerability only applies to individuals that are in the dataset (i.e., members). Penalizing accurate prediction on individuals that have not been part of the dataset (i.e., group privacy) requires a broader ethical framework. | S7: Meaningful attribute disclosure vulnerability only applies to individuals that are in the dataset (i.e., members). Penalizing accurate prediction on individuals that have not been part of the dataset (i.e., group privacy) requires a broader ethical framework. |
| **Explanation** | E7: In technical privacy research an increasingly accepted principle is to make sure that being a member in a dataset does not increase the likelihood of an adversary gaining sensitive information about an individual so that everything that can be learned about the individual can also be learned without them being a member of the dataset. From this perspective, it follows that inferences about non-members cannot be attribute disclosure in the sense of a privacy compromise. This would be more consistent with the goals of research where inference about non-members results from publishing aggregate statistics and prognostic results. | E7: In technical privacy research an increasingly accepted principle is to make sure that being a member in a dataset does not increase the likelihood of an adversary gaining sensitive information about an individual so that everything that can be learned about the individual can also be learned without them being a member of the dataset. From this perspective, it follows that inferences about non-members cannot be attribute disclosure in the sense of a privacy compromise. This would be more consistent with the goals of research where inference about non-members results from publishing aggregate statistics and prognostic results. | E7: In technical privacy research an increasingly accepted principle is to make sure that being a member in a dataset does not increase the likelihood of an adversary gaining sensitive information about an individual so that everything that can be learned about the individual can also be learned without them being a member of the dataset. From this perspective, it follows that inferences about non-members cannot be attribute disclosure in the sense of a privacy compromise. This would be more consistent with the goals of research where inference about non-members results from publishing aggregate statistics and prognostic results. |
| **Sco re** | Strongly Disagree: 0/13 (0.0%) | Strongly Disagree: 0/13 (0.0%) | Strongly Disagree: 0/13 (0.0%) |

| | | | |
|---|---|---|---|
| | Disagree: 2/13 (15.4%)<br>Neutral: 0/13 (0.0%)<br>Agree: 4/13 (30.8%)<br>Strongly Agree: 7/13 (53.8%) | Disagree: 2/13 (15.4%)<br>Neutral: 0/13 (0.0%)<br>Agree: 3/13 (23.1%)<br>Strongly Agree: 8/13 (61.5%) | **Disagree: 1/13 (7.7%)**<br>**Neutral: 1/13 (7.7%)**<br>Agree: 1/13 (7.7%)<br>Strongly Agree: 10/13 (76.9%) |
| **Qualitative Analysis** | **Qualitative Analysis**<br>While achieving broad agreement among the panelists (11/13, 84.6%), this statement also resulted in 1/13 (7.7%) rating on disagreement and 1/13 (7.7%) on uncertainty. There were no counterarguments in the key topics identified regarding this statement. However, there are opinions in the literature that would support a different conclusion. A legal analysis, for example, describes that inferring information about non-members could indeed be interpreted as a privacy violation [4]. At the same time, however, the authors acknowledge that in the United States (US) this would probably raise First Amendment challenges. The First Amendment in the US protects freedom of speech and inference constitutes speech. Similarly, it has been proposed that the concept of privacy can be extended to groups or to collective privacy in the context of big data [5,6]. The remaining disagreement may be in line with these opinions, may be opposed to the idea of a broader ethical framework or result from misunderstanding and/or ambiguity in the statement. | | |
| **Score Distribution Plot** | 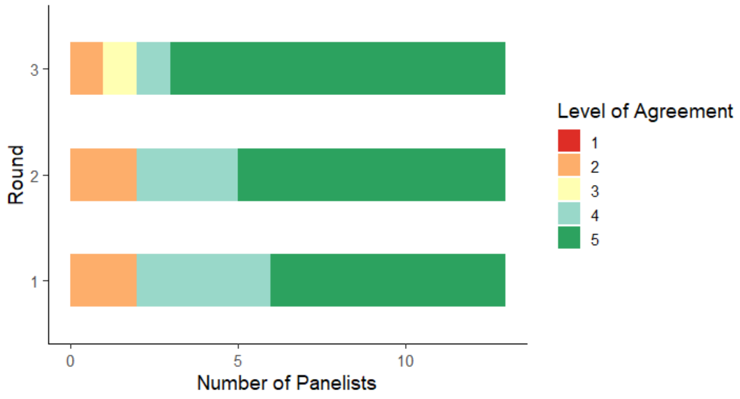 | | |

**Knowledge Generation: A Non-Member Baseline (S8)**

**Table S9. Statement S8.**

| | **Round 1** | **Round 2** | **Round 3 (Final Round)** |
|---|---|---|---|
| **Statement** | S8: A relative attribute disclosure vulnerability that takes a non-member baseline into account is meaningful. | S8: A relative attribute disclosure vulnerability that takes a non-member baseline into account is meaningful. | S8: A relative attribute disclosure vulnerability that takes a non-member baseline into account is meaningful. |
| **Explanation** | E8: A baseline derived from the prediction accuracy experienced by non-members distinguishes attribute disclosure vulnerability from knowledge generation. In technical privacy research an increasingly accepted principle is to make sure that being a member in a dataset does not materially increase the likelihood of an adversary gaining sensitive information about an individual so that everything that can be learned about the individual can also be learned without them being a member of the dataset. From this perspective, it follows that inferences about non-members cannot be attribute disclosure in the sense of a privacy compromise. | E8: A baseline derived from the prediction accuracy experienced by non-members distinguishes attribute disclosure vulnerability from knowledge generation. In technical privacy research an increasingly accepted principle is to make sure that being a member in a dataset does not materially increase the likelihood of an adversary gaining sensitive information about an individual so that everything that can be learned about the individual can also be learned without them being a member of the dataset. From this perspective, it follows that inferences about non-members cannot be attribute disclosure in the sense of a privacy compromise. | E8: A baseline derived from the prediction accuracy experienced by non-members distinguishes attribute disclosure vulnerability from knowledge generation. In technical privacy research an increasingly accepted principle is to make sure that being a member in a dataset does not materially increase the likelihood of an adversary gaining sensitive information about an individual so that everything that can be learned about the individual can also be learned without them being a member of the dataset. From this perspective, it follows that inferences about non-members cannot be attribute disclosure in the sense of a privacy compromise. |
| **Score Distribution** | Strongly Disagree: 0/13 (0.0%)<br>Disagree: 0/13 (0.0%)<br>Neutral: 0/13 (0.0%)<br>Agree: 6/13 (46.1%)<br>Strongly Agree: 7/13 (53.8%) | Strongly Disagree: 0/13 (0.0%)<br>Disagree: 0/13 (0.0%)<br>Neutral: 0/13 (0.0%)<br>Agree: 5/13 (38.5%)<br>Strongly Agree: 8/13 (61.5%) | Strongly Disagree: 0/13 (0.0%)<br>Disagree: 0/13 (0.0%)<br>Neutral: 0/13 (0.0%)<br>Agree: 3/13 (23.1%)<br>Strongly Agree: 10/13 (76.9%) |

| Score Distribution Plot | 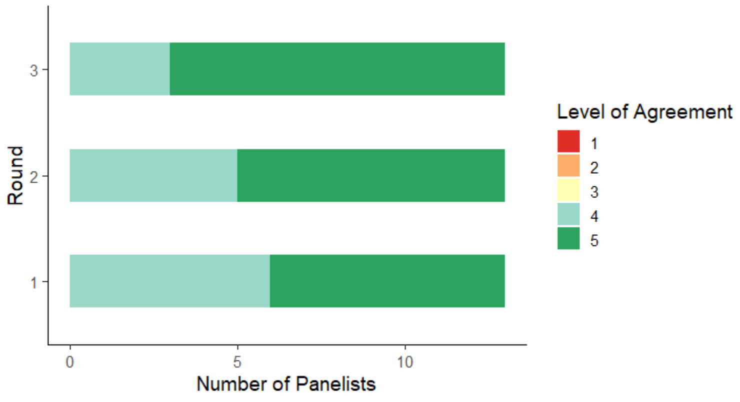  |
|---|---|

## Interpretation Beyond Random Guessing (S9)

**Table S10. Statement S9**

| | **Round 1** | **Round 2** | **Round 3 (Final Round)** |
|---|---|---|---|
| **Statement** | S9: In attribute disclosure vulnerability, a relative vulnerability higher than its threshold is only considered as unacceptably high when the absolute vulnerability is higher than its threshold. | S9: In attribute disclosure vulnerability, a relative vulnerability higher than its threshold is only considered as unacceptably high when the absolute vulnerability is higher than its threshold. | S9: In attribute disclosure vulnerability, a relative vulnerability higher than its threshold is only considered as unacceptably high when the absolute vulnerability is higher than its threshold. |
| **Explanation** | E9: The relative metric cannot be interpreted uniformly across the entire scale and is probably not always informative by itself. Therefore the decision rule for acceptable attribute disclosure needs to also consider the absolute vulnerability value. | E9: The relative metric cannot be interpreted uniformly across the entire scale and is probably not always informative by itself. Therefore the decision rule for acceptable attribute disclosure needs to also consider the absolute vulnerability value. | E9: The relative metric cannot be interpreted uniformly across the entire scale and is probably not always informative by itself. Therefore the decision rule for acceptable attribute disclosure needs to also consider the absolute vulnerability value. |
| **Score Distribution** | Strongly Disagree: 1/13 (7.7%)<br>Disagree: 0/13 (0.0%)<br>Neutral: 2/13 (15.4%)<br>Agree: 9/13 (69.2%)<br>Strongly Agree: 1/13 (7.7%) | Strongly Disagree: 1/13 (7.7%)<br>Disagree: 0/13 (0.0%)<br>Neutral: 1/13 (7.7%)<br>Agree: 9/13 (69.2%)<br>Strongly Agree: 2/13 (15.4%) | Strongly Disagree: 0/13 (0.0%)<br>**Disagree: 1/13 (7.7%)**<br>Neutral: 0/13 (0.0%)<br>Agree: 10/13 (76.9%)<br>Strongly Agree: 2/13 (15.4%) |

| | |
|---|---|
| **Qualitative Analysis** | Among our experts, there was 1/13 (7.7%) who disagreed with this statement. Counterarguments in key topics related to this statement did not question the relevance of the absolute scale but rather the application of a threshold in this context or the use of two metrics instead of a proportional metric as in [7]. The discussion on thresholds in general is presented below in the main manuscript. A proportional metric as in [7] is defined relative to the maximum possible attribute disclosure vulnerability beyond the non-member baseline:<br><br>$$R = \frac{r_{train} - r_{control}}{1 - r_{control}} \quad (1)$$<br><br>where $r_{train}$ is the prediction accuracy for members and $r_{control}$ the one for non-members. The idea is that this metric accounts for both absolute and relative scale. It treats differences at the upper end of the scale as more severe than those at the lower end. At the same time, however, it penalizes high non-member baselines and thereby penalizes potentially relevant population-level information (i.e., knowledge generation). Assuming, for example, a non-member baseline of 0.9 accuracy. This is commonly seen as a very good or excellent model [8]. The high accuracy on unseen data (i.e., non-members) suggests that relevant and externally valid population-level information have been derived from the data. At this end of the scale, however, a slightly higher member accuracy of 0.95 would result in a high proportional vulnerability estimate of 0.5. Using a threshold of 0.2 which has been used for such ratios elsewhere [9–11], this would be considered as an unacceptably high vulnerability. A proportional metric thus comes with the challenge that potentially highly valuable insights would be considered as disclosure even though the actual difference is negligible. |
| **Score Distribution Plot** | 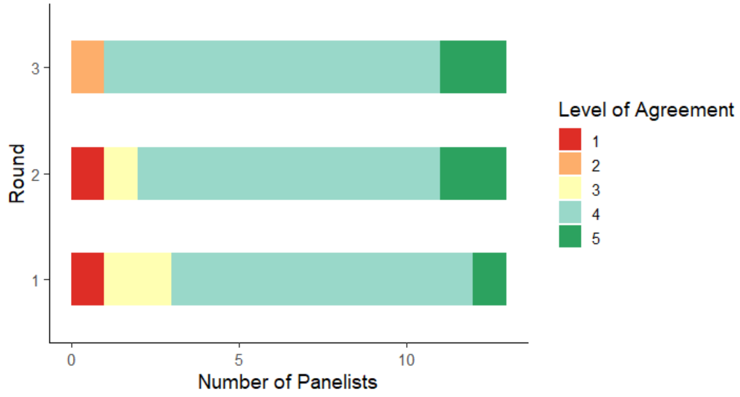 |

## Differential Privacy (S10 & S17)

**Table S11. Statement S10**

| | **Round 1** | **Round 2** | **Round 3 (Final Round)** |
|---|---|---|---|
| **Statement** | S10: Non-differentially private SDG models should be preferred over current applications of Differential Privacy in SDG. | **S10:** omitted | NA |
| **Explanation** | E10: The value of the privacy budget epsilon has no uniform translation into privacy and can only be interpreted contextually. The consequences of the privacy budget are not the same across different statistical analyses and its value cannot be easily translated into empirical privacy. Differentially private synthetic data would therefore still require a privacy evaluation. Current implementations cannot provide a reasonable privacy-utility trade-off. | NA | NA |
| **Commentary** | The statement was developed based on the initial report. | The statement was removed since it assessed the application of DP to SDG instead of focusing on epsilon as a privacy metric. | NA |
| **Score Distribution** | Strongly Disagree: 3/13 (23.1%)<br>Disagree: 1/13 (7.7%)<br>Neutral: 7/13 (53.8%)<br>Agree: 1/13 (7.7%)<br>Strongly Agree: 1/13 (7.7%) | NA | NA |

| | |
|---|---|
| **Score Distribution Plot** | 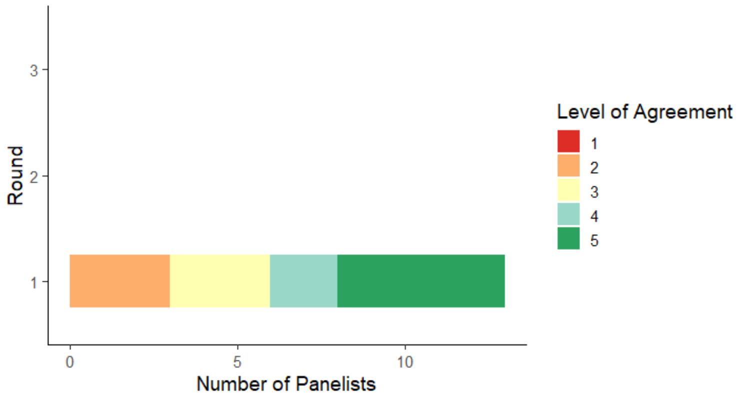 |

**Table S12. Statement S17**

| | Round 1 | Round 2 | Round 3 (Final Round) |
|---|---|---|---|
| **Statement** | NA | **S17**: The privacy budget epsilon is not an adequate metric to report disclosure vulnerability. Disclosure vulnerability would still need to be evaluated using the same metrics as those applied to non-differentially private synthetic data. | **S17.1**: The privacy budget epsilon is not an adequate metric to report disclosure vulnerability unless it is set to a value close to 0. Even when differential privacy methods are used, disclosure vulnerability would still need to be evaluated using the same metrics as those applied to non-differentially private synthetic data. |
| **Explanation** | NA | **E17**: The value of the privacy budget epsilon has no uniform translation into empirical privacy and can only be interpreted contextually. This is in particular true when the privacy budget is relatively large. Differentially private synthetic data would therefore still require a full privacy evaluation. | E17: The value of the privacy budget epsilon has no uniform translation into empirical privacy and can only be interpreted contextually. This is in particular true when the privacy budget is relatively large. Differentially private synthetic data would therefore still require a full privacy evaluation. |
| **Commentary** | NA | The statement was introduced to focus on the privacy evaluation of DP-SDG, rather than on utility or implementation challenges. | The rephrasing in the statement did not change the recommendation but increased readability. |
| **Score Distribution** | NA | Strongly Disagree: 1/13 (7.7%)<br>Disagree: 1/13 (7.7%)<br>Neutral: 1/13 (7.7%)<br>Agree: 4/13 (30.8%)<br>Strongly Agree: 6/13 (46.1%) | Strongly Disagree: 0/13 (0.0%)<br>Disagree: 0/13 (0.0%)<br>Neutral: 2/13 (15.4%)<br>Agree: 2/13 (15.4%)<br>Strongly Agree: 9/13 (69.2%) |

| | |
|---|---|
| **Score Distribution Plot** | 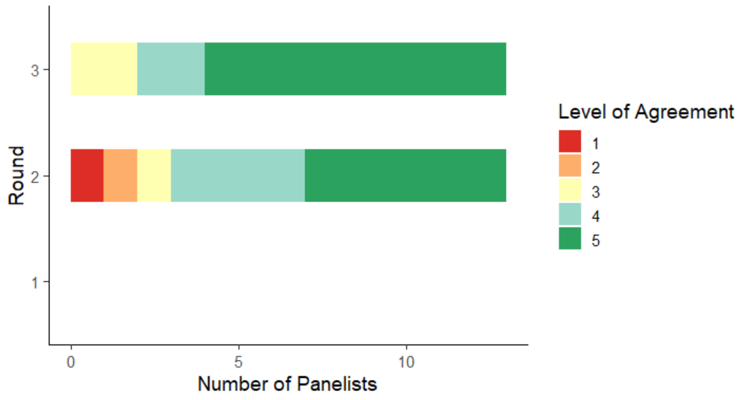 |

## Metric Interpretation and Decision-Making

### Stochasticity of the Process (S11)

**Table S13. Statement S11**

| | Round 1 | Round 2 | Round 3 (Final Round) |
|---|---|---|---|
| **Statement** | S11: When evaluating an SDG model, disclosure vulnerability metrics need to be reported for multiple (e.g. averaged) synthetic datasets. | **S11.1:** When evaluating a specific trained SDG model, disclosure vulnerability metrics need to be reported both for individual and multiple synthetic datasets (e.g. averaged across them and variation). | S11.1: When evaluating a specific trained SDG model, disclosure vulnerability metrics need to be reported both for individual and multiple synthetic datasets (e.g. averaged across them and variation). |
| **Explanation** | E11: SDG is a generative process with stochastic variability in its output. When evaluating an SDG model rather than a single synthetic dataset, an aggregate across multiple synthetic datasets from the same model would be appropriate. | **E11.1**: SDG is a generative process with stochastic variability in its output. When evaluating an SDG model rather than a single synthetic dataset, an aggregate (average and standard deviation) across multiple synthetic datasets from the same model would be appropriate. This would then reflect the vulnerability of an SDG model tied to a training dataset. However, if the decision-making scenario is data release, then the disclosure vulnerability for the specific synthetic dataset(s) may be the most relevant. Given that it is not always possible to determine a priori the exact decision-making scenario, it would be prudent to have both types of results. | E11.1: SDG is a generative process with stochastic variability in its output. When evaluating an SDG model rather than a single synthetic dataset, an aggregate (average and standard deviation) across multiple synthetic datasets from the same model would be appropriate. This would then reflect the vulnerability of an SDG model tied to a training dataset. However, if the decision-making scenario is data release, then the disclosure vulnerability for the specific synthetic dataset(s) may be the most relevant. Given that it is not always possible to determine a priori the exact decision-making scenario, it would be prudent to have both types of results. |
| **Commentary** | The statement was developed based on the initial report. | The rephrasing in the statement included the implicit assumptions about other decision-making scenarios. The explanation was rephrased to include | NA |

| | | | |
|---|---|---|---|
| | | further details from the report as a rationale for this statement. | |
| **Score Distribution** | Strongly Disagree: 0/13 (0.0%)<br>Disagree: 2/13 (15.4%)<br>Neutral: 2/13 (15.4%)<br>Agree: 4/13 (30.8%)<br>Strongly Agree: 5/13 (38.5%) | Strongly Disagree: 0/13 (0.0%)<br>Disagree: 0/13 (0.0%)<br>Neutral: 1/13 (7.7%)<br>Agree: 5/13 (38.5%)<br>Strongly Agree: 7/13 (53.8%) | Strongly Disagree: 0/13 (0.0%)<br>Disagree: 0/13 (0.0%)<br>Neutral: 1/13 (7.7%)<br>Agree: 5/13 (38.5%)<br>Strongly Agree: 7/13 (53.8%) |
| **Score Distribution Plot** | 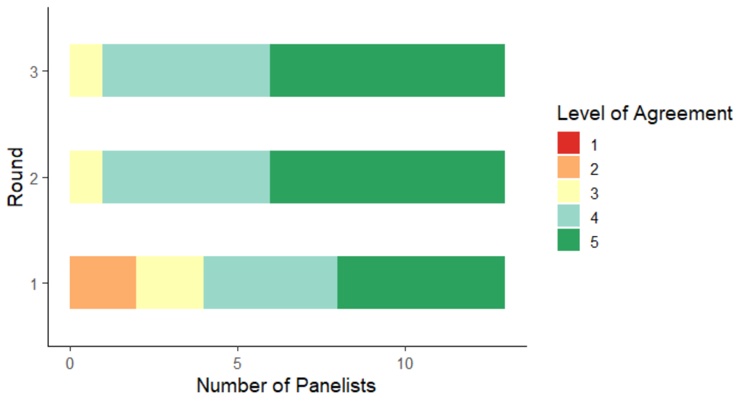 | | |

## Thresholds (S12, S13, S14 & S15)

**Table S14. Statement S12**

| | **Round 1** | **Round 2** | **Round 3 (Final Round)** |
|---|---|---|---|
| **Statement** | S12: In membership disclosure vulnerability, an absolute vulnerability (i.e., F1 value) lower than or equal to 0.5 can be considered as acceptably low. | **S12**: omitted | NA |
| **Explanation** | E12: Membership disclosure vulnerability is a binary classification problem. Accordingly, an accuracy of 0.5 would be a random guess between members and non-members. This threshold applies to both, the naïve (inherent) vulnerability and the absolute vulnerability of the synthetic data. | NA | NA |
| **Commentary** | NA | The statement was removed and replaced by S16 since it does not hold for *F1 score* which is commonly used to report membership disclosure vulnerability. | NA |
| **Score Distribution** | Strongly Disagree: 2/13 (15.4%)<br>Disagree: 1/13 (7.7%)<br>Neutral: 3/13 (23.1%)<br>Agree: 5/13 (38.5%)<br>Strongly Agree: 2/13 (15.4%) | NA | NA |

| | |
|---|---|
| **Score Distribution Plot** | 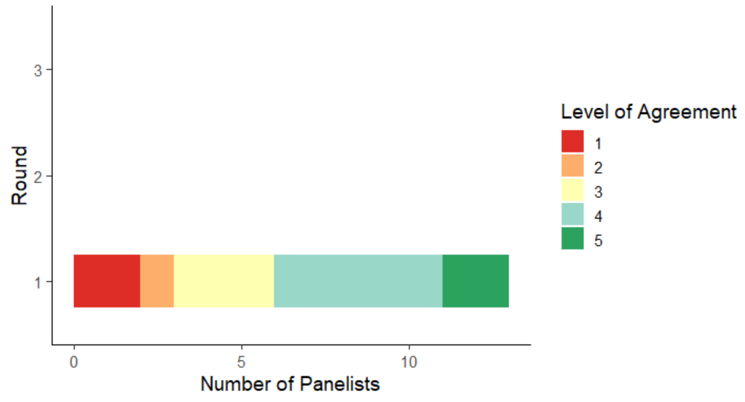 |

**Table S15. Statement S13**

| | Round 1 | Round 2 | Round 3 (Final Round) |
|---|---|---|---|
| **Statement** | S13: In membership disclosure vulnerability, a relative vulnerability (i.e., F_rel value) lower than or equal to 0.2 can be considered as acceptably low. | **S13.1**: As a default threshold value for membership disclosure vulnerability, a relative vulnerability (i.e., F_rel value) less than or equal to 0.2 can be considered as acceptably low. | **S13.2**: As an anchor for membership disclosure vulnerability, a relative F1 score vulnerability (i.e., F_rel value) of 0.2 is suggested. |
| **Explanation** | E13: A common way to define thresholds is to rely on precedents. The threshold that have been used in the literature is 0.2 for relative metrics when compared to a naïve baseline (F_rel). | **E13.1**: The relative metric (F_rel) is a good way to account for the naïve baseline in a non uniformly interpretable F1. This threshold has been used in the literature for the relative membership disclosure metric (F_rel). In practice, thresholds that are recommended based on precedents may be adjusted up or down based on the context. Such adjustments need to be considered carefully and be justified. This context is characterized by the sensitivity of the data, potential harm and appropriateness of consent and notice. | **E13.2**: The relative metric (F_rel) is a good way to account for a naïve baseline to interpret F1. This threshold has been used in the literature for the relative membership disclosure metric (F_rel). In practice, thresholds that are recommended based on precedents may be adjusted up or down based on the context. Such adjustments need to be considered carefully and be justified. This context is characterized by the sensitivity of the data, potential harm and appropriateness of consent and notice. |
| **Commentary** | NA | The rephrasing in the statement did not change the recommendation but incorporated parts of the explanation. The explanation was rephrased to include further details from the report as a rationale for this statement. | The rephrasing in the statement did not change the recommendation but clarified the meaning of a default threshold / anchor value. The explanation was rephrased to increase readability. |
| **Score Distributio** | Strongly Disagree: 2/13 (15.4%)<br>Disagree: 0/13 (0.0%)<br>Neutral: 6/13 (46.1%) | Strongly Disagree: 3/13 (23.1%)<br>Disagree: 0/13 (0.0%)<br>Neutral: 7/13 (53.8%) | Strongly Disagree: 1/13 (7.7%)<br>Disagree: 1/13 (7.7%)<br>Neutral: 7/13 (53.8%) |

<table>
<tr><td></td><td>Agree: 4/13 (30.8%)<br><br>Strongly Agree: 1/13 (7.7%)</td><td>Agree: 2/13 (15.4%)<br><br>Strongly Agree: 1/13 (7.7%)</td><td>Agree: 2/13 (15.4%)<br><br>Strongly Agree: 2/13 (15.4%)</td></tr>
<tr><td>Qualitative Analysis</td><td colspan="3">The panel’s ratings reflected a consensus on uncertainty regarding this statement. In the qualitative analysis, two key topics could be identified: First, any threshold, anchor or default value for disclosure vulnerability is considered as inadequate. One reason that was given for this perspective is the contextual interpretation of privacy vulnerability as described above. Another reason is that vulnerability can be reported very differently, for example, based on an average value as well as a maximum value. A concern is that when giving an anchor value people may rely on this value without considering relevant aspects that may trigger adjustments to the value. And second, there is not enough evidence for the very anchor value proposed. Implications of this result are discussed below.</td></tr>
<tr><td>Score Distribution Plot</td><td colspan="3">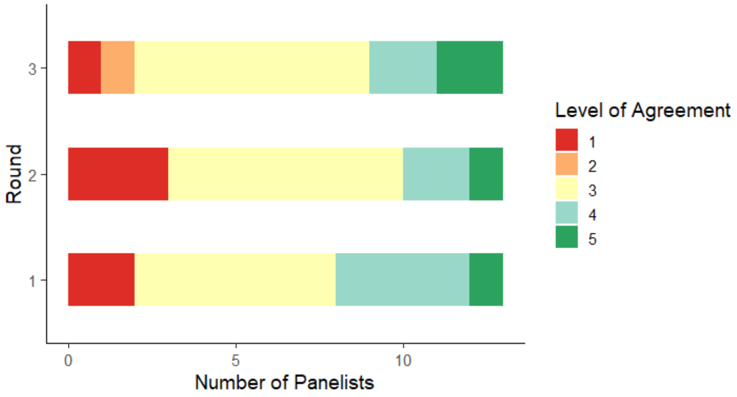
</td></tr>
</table>

**Table S16. Statement S14**

| | Round 1 | Round 2 | Round 3 (Final Round) |
|---|---|---|---|
| **Statement** | S14: In attribute disclosure vulnerability, a distance to the non-member baseline lower than or equal to 0.15 can be considered as acceptably low. | **S14.1**: As a default threshold value for attribute disclosure vulnerability, a difference from the non-member baseline less than or equal to 0.15 can be considered as acceptably low. | **S14.1**: omitted |
| **Explanation** | E14: For attribute disclosure vulnerability, thresholds could be based on common interpretations of prediction accuracy. Across different labeling systems, labels such as poor, moderate or good prediction accuracy typically change with differences of 0.1 to 0.2 in AUC. A threshold of 0.15 therefore appears reasonable. The threshold would apply to the difference in AUC between member and non-member accuracy, not to a proportional metric (such as a Kappa-like metrics that are relative to the maximum possible attribute disclosure vulnerability beyond knowledge generation). | **E14.1**: For attribute disclosure vulnerability, thresholds could be based on common interpretations of prediction accuracy. Across different labeling systems, labels such as poor, moderate or good prediction accuracy typically change with differences of 0.1 to 0.2 in AUC. A threshold of 0.15 therefore appears reasonable. The threshold would apply to the difference in AUC between member and non-member accuracy. In practice, thresholds that are recommended based on precedents may be adjusted up or down based on the context. Such adjustments need to be considered carefully and be justified. This context is characterized by the sensitivity of the data, potential harm and appropriateness of consent and notice. | NA |
| **Commentary** | NA | The rephrasing in the statement did not change the recommendation but incorporated parts of the explanation. The explanation was rephrased to include further details from the report as a rationale for this statement. | The statement was removed since it was overly speculative drawing from evidence that was not directly related to privacy. |

| | | | |
|---|---|---|---|
| **Score Distribution** | Strongly Disagree: 1/13 (7.7%)<br>Disagree: 2/13 (15.4%)<br>Neutral: 6/13 (46.1%)<br>Agree: 2/13 (15.4%)<br>Strongly Agree: 2/13 (15.4%) | Strongly Disagree: 3/13 (23.1%)<br>Disagree: 1/13 (7.7%)<br>Neutral: 7/13 (53.8%)<br>Agree: 1/13 (7.7%)<br>Strongly Agree: 1/13 (7.7%) | NA |
| **Score Distribution Plot** | 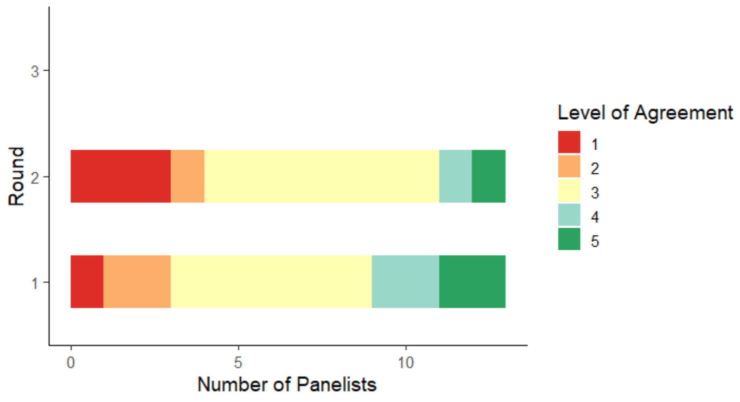 | | |

**Table S17. Statement S15**

| | Round 1 | Round 2 | Round 3 (Final Round) |
|---|---|---|---|
| **Statement** | S15: In attribute disclosure vulnerability, an absolute vulnerability (i.e., accuracy of a member) lower than or equal to 0.6 can be considered as acceptably low. | **S15.1**: As a default threshold value for attribute disclosure vulnerability, an absolute vulnerability (i.e., accuracy of predicting a member sensitive attribute) less than or equal to 0.6 can be considered as acceptably low. | **S15.1**: omitted |
| **Explanation** | E15: Across different labeling systems, poor accuracy is mainly assigned to an AUC lower than or equal to 0.6. Following this interpretation, a threshold of 0.6 could be established for absolute vulnerability, meaning that only accuracies that exceed poor prediction (i.e., 0.6) can be meaningful disclosure. | **E15.1**: Across different labeling systems, poor accuracy is mainly assigned to an AUC lower than or equal to 0.6. Following this interpretation, a threshold of 0.6 could be established for absolute vulnerability, meaning that only accuracies that exceed poor prediction (i.e., 0.6) can be meaningful disclosure. In practice, thresholds that are recommended based on precedents may be adjusted up or down based on the context. Such adjustments need to be considered carefully and be justified. This context is characterized by the sensitivity of the data, potential harm and appropriateness of consent and notice. | NA |
| **Commentary** | NA | The rephrasing in the statement did not change the recommendation but incorporated parts of the explanation. The explanation was rephrased to include further details from the report as a rationale for this statement. | The statement was removed since it was overly speculative drawing from evidence that was not directly related to privacy. |

| | | | |
|---|---|---|---|
| **Score Distribution** | Strongly Disagree: 1/13 (7.7%)<br>Disagree: 2/13 (15.4%)<br>Neutral: 4/13 (30.8%)<br>Agree: 4/13 (30.8%)<br>Strongly Agree: 2/13 (15.4%) | Strongly Disagree: 2/13 (15.4%)<br>Disagree: 1/13 (7.7%)<br>Neutral: 3/13 (23.1%)<br>Agree: 4/13 (30.8%)<br>Strongly Agree: 3/13 (23.1%) | NA |
| **Score Distribution Plot** | 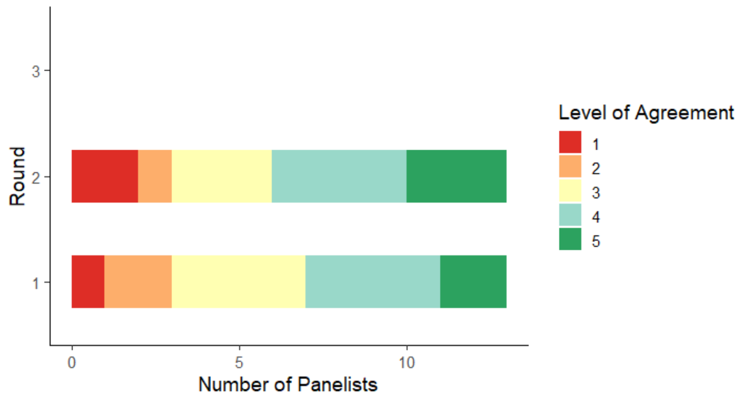 | | |

## Supplemental References

1. Pilgram, L., Dankar, F.K., Drechsler, J., Elliot, M., Domingo-Ferrer, J., Francis, P., Kantarcioglu, M., Malin, B., Muralidhar, K., Myles, P., et al. (2025). A Consensus Privacy Metrics Framework for Synthetic Data - Critical Analysis of Privacy Metrics (Report). OSF, https://osf.io/vz5x9.

2. Khodyakov, D., Grant, S., Kroger, J., and Bauman, M. (2023). RAND Methodological Guidance for Conducting and Critically Appraising Delphi Panels (RAND Corporation). https://www.rand.org/pubs/tools/TLA3082-1.html.

3. Malterud, K. (2001). Qualitative research: standards, challenges, and guidelines. Lancet *358*, 483–488. https://doi.org/10.1016/S0140-6736(01)05627-6.

4. Gal, M., and Lynskey, O. (2023). Synthetic Data: Legal Implications of the Data-Generation Revolution. 109 Iowa Law Review, Forthcoming, https://doi.org/10.2139/ssrn.4414385.

5. Mühlhoff, R. (2021). Predictive privacy: towards an applied ethics of data analytics. Ethics Inf Technol *23*, 675–690. https://doi.org/10.1007/s10676-021-09606-x.

6. Mantelero, A. (2017). From Group Privacy to Collective Privacy: Towards a New Dimension of Privacy and Data Protection in the Big Data Era. In Group Privacy: New Challenges of Data Technologies, L. Taylor, L. Floridi, and B. van der Sloot, eds. (Springer International Publishing), pp. 139–158. https://doi.org/10.1007/978-3-319-46608-8_8.

7. Giomi, M., Boenisch, F., Wehmeyer, C., and Tasnádi, B. (2023). A Unified Framework for Quantifying Privacy Risk in Synthetic Data. Preprint at arXiv, https://doi.org/10.48550/arXiv.2211.10459.

8. Hond, A.A.H. de, Steyerberg, E.W., and Calster, B. van (2022). Interpreting area under the receiver operating characteristic curve. The Lancet Digital Health *4*, e853–e855. https://doi.org/10.1016/S2589-7500(22)00188-1.

9. Mendelevitch, O., and Lesh, M.D. (2021). Fidelity and Privacy of Synthetic Medical Data. Preprint at arXiv, 2101.08658 [cs].

10. El Emam, K., Mosquera, L., and Fang, X. (2022). Validating A Membership Disclosure Metric For Synthetic Health Data. JAMIA Open *5*, ooac083. https://doi.org/10.1093/jamiaopen/ooac083.

11. El Kababji, S., Mitsakakis, N., Fang, X., Beltran-Bless, A.-A., Pond, G., Vandermeer, L., Radhakrishnan, D., Mosquera, L., Paterson, A., Shepherd, L., et al. (2023). Evaluating the Utility and Privacy of Synthetic Breast Cancer Clinical Trial Data Sets. JCO Clin Cancer Inform, e2300116. https://doi.org/10.1200/CCI.23.00116.
