# Supplementary material for: A consensus privacy metrics framework for synthetic data
Source: Patterns (N Y). 2025 Jul 29;6(10):101320. doi: 10.1016/j.patter.2025.101320 (PMC12546437; doi:10.1016/j.patter.2025.101320)
Supplement: Document S2. Article plus supplemental information [file mmc2.pdf]

Patterns

Article

A consensus privacy metrics framework for synthetic data

Graphical abstract

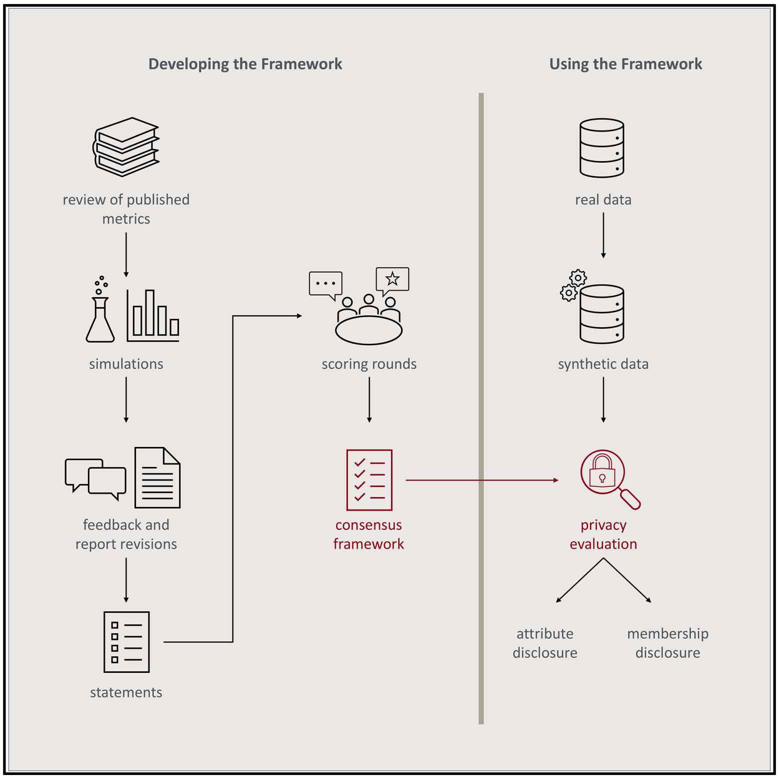

Authors

Lisa Pilgram, Fida Kamal Dankar, Jörg Drechsler, ..., Jean Louis Raisaro, Chao Yan, Khaled El Emam

Correspondence

lpilgram@ehealthinformation.ca (L.P.), kelemam@ehealthinformation.ca (K.E.E.)

In brief

Synthetic data can enable privacy-preserving data sharing across multiple sectors, but residual privacy vulnerabilities must be evaluated. Through a formal consensus process, this study developed an expert consensus framework to evaluate privacy in synthetic data.

Highlights

- Privacy metrics for synthetic data should make their assumptions explicit
- Membership disclosure and attribute disclosure should be evaluated in synthetic data
- Record-level similarity is not a valid proxy for identity disclosure
- Thresholds are needed to guide decision-making and should be a focus in future work

Pilgram et al., 2025, Patterns 6, 101320
October 10, 2025 © 2025 The Author(s). Published by Elsevier Inc.
https://doi.org/10.1016/j.patter.2025.101320

CellPress

Patterns

CellPress
OPEN ACCESS

Article

A consensus privacy metrics framework for synthetic data

Lisa Pilgram,[1,2,3,*] Fida Kamal Dankar,[2] Jörg Drechsler,[4,5,6] Mark Elliot,[7] Josep Domingo-Ferrer,[8] Paul Francis,[9] Murat Kantarcioglu,[10] Linglong Kong,[11] Bradley Malin,[12,13,14] Krishnamurty Muralidhar,[15] Puja Myles,[16] Fabian Prasser,[17] Jean Louis Raisaro,[18] Chao Yan,[12] and Khaled El Emam[1,2,19,*]

[1]School of Epidemiology and Public Health, University of Ottawa, Ottawa, ON K1H 8M5, Canada
[2]CHEO Research Institute, Ottawa, ON K1H 8L1, Canada
[3]Department of Nephrology and Medical Intensive Care, Charité – Universitätsmedizin Berlin, 10117 Berlin, Germany
[4]Department for Statistical Methods, Institute for Employment Research, Nuernberg, 90478 Bavaria, Germany
[5]Department of Statistics, Ludwig-Maximilians-Universität, Munich, 80539 Bavaria, Germany
[6]Joint Program in Survey Methodology, University of Maryland, College Park, MD 20742, USA
[7]The Cathie Marsh Institute Research, School of Social Sciences, University of Manchester, M13 9PL Manchester, UK
[8]Department of Computer Engineering and Mathematics, Universitat Rovira i Virgili, Tarragona, 43007 Catalonia, Spain
[9]Max Planck Institute for Software Systems, Kaiserslautern, 67663 Rhineland-Palatinate, Germany
[10]Department of Computer Science, Virginia Tech, Blacksburg, VA 24060, USA
[11]Department of Mathematical and Statistical Sciences, University of Alberta, Edmonton, AB T6G 2R3, Canada
[12]Department of Biomedical Informatics, Vanderbilt University Medical Center, Nashville, TN 37203, USA
[13]Department of Biostatistics, Vanderbilt University Medical Center, Nashville, TN 37203, USA
[14]Department of Computer Science, Vanderbilt University, Nashville, TN 37240, USA
[15]Department of Marketing and Supply Chain Management, Price College of Business, University of Oklahoma, Norman, OK 73019, USA
[16]Medicines and Healthcare Products Regulatory Agency, SW1W 9SZ London, UK
[17]Berlin Institute of Health at Charité – Universitätsmedizin Berlin, Medical Informatics Group, 10117 Berlin, Germany
[18]Biomedical Data Science Center, University Hospital Lausanne, 1011 Lausanne, Switzerland
[19]Lead contact
*Correspondence: lpilgram@ehealthinformation.ca (L.P.), kelemam@ehealthinformation.ca (K.E.E.)
https://doi.org/10.1016/j.patter.2025.101320

THE BIGGER PICTURE Synthetic data generation offers a potential solution to provide high-quality data while preserving privacy, thereby acting as a research and innovation accelerator across sectors. The idea is to train models, often leveraging artificial intelligence, to learn the statistical properties of real data and generate synthetic data from these models. Synthetic data should consequently not have a one-to-one mapping to real data. To prevent residual privacy vulnerabilities, we need a standardized evaluation for synthetic data. This study presents expert consensus recommendations for evaluating privacy in synthetic data. A standardized evaluation can facilitate the adoption of synthetic data generation, contribute to better access to and sharing of data, and ultimately promote the use of data for secondary research purposes, innovation, reproducibility, and transparency.

SUMMARY

Synthetic data generation is a promising approach for sharing data for secondary purposes in sensitive sectors. However, to meet ethical standards and legislative requirements, it is necessary to demonstrate that the privacy of the individuals upon which the synthetic records are based is adequately protected. Through an expert consensus process, we developed a framework for privacy evaluation in synthetic data. The most commonly used metrics measure similarity between real and synthetic data and are assumed to capture identity disclosure. Our findings indicate that they lack precise interpretation and should be avoided. There was consensus on the importance of membership and attribute disclosure, both of which involve inferring personal information. The framework provides recommendations to effectively measure these types of disclosures, which also apply to differentially private synthetic data if the privacy budget is not close to zero. We further present future research opportunities to support widespread adoption of synthetic data.

© 2025 The Author(s). Published by Elsevier Inc.
This is an open access article under the CC BY license (http://creativecommons.org/licenses/by/4.0/).

INTRODUCTION

Data access for secondary analysis remains a challenge,[1] sometimes taking months,[2,3] with success rates of, for example, obtaining data for meta-analysis projects ranging from 0% to 58%.[3–8] To address access challenges, there is growing interest in using synthetic data generation (SDG) techniques to enable broader sharing of data for research and analysis.[9–20] In health research, for example, synthetic datasets have been made available for research, including the National COVID Cohort Collaborative (N3C) of the US National Institutes of Health,[21] the Centers for Medicare & Medicaid Services Data Entrepreneur's Synthetic Public Use files, synthetic cardiovascular and COVID-19 datasets available from the Clinical Practice Research Datalink (CPRD) in the United Kingdom,[11] cancer data from Public Health England (Simulacrum), synthetic variants of the French public health system claims and hospital dataset (SNDS), and synthetic microdata from Israel's National Registry of Live Births.[22] Furthermore, the authors of several studies have recently been making synthetic variants of data used in their research papers publicly available to enable open science.[23–26]

Such broad sharing of synthetic datasets requires strong assurances that the privacy of individuals is protected. Unlike statistical disclosure control methods that create protected data by perturbing original data or reducing their detail,[27–30] synthetic data are generated by sampling records from a distribution learned during model training.[31] It is thereby grounded in the original data but should not preserve a one-to-one mapping between the synthetic records and real individuals. For this reason, one might naively conclude that synthetic data have a low disclosure vulnerability. However, if the SDG model, for example, overfits the original data, then an adversary may still be able to learn sensitive information about individuals. Therefore, a privacy assessment is still needed to demonstrate that the generated synthetic datasets do indeed have low disclosure vulnerability.

In certain domains, such as the healthcare sector, a privacy assessment of synthetic data is of particular importance considering the sensitive nature of the data and the more significant potential harm that would arise from privacy breaches. However, a recent review showed that privacy is not always assessed when SDG is used as a privacy enhancing technology for healthcare data.[32] These and other authors[33,34] posit that the lack of consensus on how to measure privacy vulnerabilities in synthetic data may have contributed to those vulnerabilities not being evaluated at all. Similarly, various calls for developing privacy frameworks and standards for synthetic health data have just recently been published.[35–37]

Therefore, there is a need to assess and reach a consensus on the current work on privacy evaluation in synthetic data. Such a consolidation will enable the comparison of SDG methods, facilitate the development of standardized benchmark datasets and software, support decisions on sharing synthetic data, and establish greater regulatory certainty for synthetic data.

The objectives of this study were therefore to develop a consensus framework for how to evaluate privacy vulnerability in synthetic data. Key terms used throughout this study such as "SDG" or "privacy vulnerability" are defined in Table 1. The approach taken in the study was to convene a global panel of privacy experts who contributed to two objectives:

(1) the critical analysis of privacy metrics and evaluation practices in synthetic data based on the current body of work, to identify their strengths and weaknesses and
(2) the development of consensus-based recommendations on how to evaluate privacy in synthetic data.

During our critical analysis (first objective), it became clear to us that many of the proposed privacy metrics do not provide an interpretable vulnerability estimate for synthetic data. Rather than identifying a single "best" metric, we focus on recommending good practices for the use and interpretation of privacy metrics that are currently used in practice to measure relevant vulnerabilities in synthetic data. We also discourage metrics and practices lacking meaningful interpretability and outline directions for future research. While the focus of this manuscript is on the consensus recommendations (second objective), we also report findings from the critical analysis (objective 1) that directly informed the development of the framework. The full critical analysis is available as a stand-alone report.[47]

RESULTS

The synthetic data privacy literature typically refers to the three disclosure concepts as indicated in Table 1: identity disclosure, membership disclosure, and attribute disclosure. A wide variety of metrics have been used to evaluate these concepts in synthetic data.[32,48–50] Most of them can be classified into metrics that measure membership or attribute disclosure. Metrics that evaluate record-level similarity often do not explicitly define the type of disclosure they target, but there are examples where it has explicitly been used to approximate identity disclosure.[51,52] Parameters such as the privacy (loss) budget used in the context of differentially private SDG (DP-SDG) to characterize the privacy of synthetic data do not fit into any of these categories and can therefore be seen as a stand-alone category.

Within each category, multiple different metrics have been described in the literature. The recommendations presented in this paper were developed through a systematic consensus process (Delphi study), preceded by a literature-informed critical analysis of these metrics. This critical analysis justified the development of proposed recommendations, referred to as statements, which were presented to the panelists in the Delphi rounds. The analysis itself was provided to the panelists as a background report throughout the study (available in Pilgram et al. on the Open Science Frameworf, OSF[47]).

The statements were related to the four categories of privacy metrics (i.e., identity disclosure, membership disclosure, attribute disclosure, and DP) as well as overarching considerations. During the Delphi rounds, each statement was accompanied by an explanation to provide more clarity and relevant definitions. Panelists indicated their agreement with each statement on a five-point Likert scale, with 1 indicating "strongly disagree" and 5 indicating "strongly agree." Some statements underwent adjustments throughout the study process based on the panelists' feedback, as reflected in statement version numbers (see Figure 1). Most changes involved rephrasing that incorporated

Table 1. Definition of key terms

Adversary<br>An adversary is an "individual or unit that can, whether intentionally or not, exploit potential vulnerabilities."[38] The goal is to identify an individual or infer personal information about him or her. The adversary is often conceptualized as the anticipated data recipient.[39]
Attribute disclosure<br>Attribute disclosure is when an adversary can infer sensitive information about a target individual based on a dataset's attributes.[27,40,41]
Direct identifier<br>A direct identifier is an attribute that uniquely identifies an individual (e.g., Social Security number).[38] We assume that direct identifiers are not part of the training dataset.
Identity disclosure<br>Identity disclosure is concerned about correctly assigning an identity to a record in a dataset and encompasses the idea of singling out or linking records.[38,42]
Membership disclosure<br>Membership disclosure is the ability of an adversary to determine that a target individual was in the original dataset used to train the SDG model (i.e., a member of the training dataset).[43]
Privacy<br>In this paper, we mean informational privacy, which is concerned with whether personal information is disclosed, rather than the potential harm that may result from such disclosure.
Privacy metric<br>A privacy metric refers to a specific implementation to measure privacy in synthetic data, typically defined in a single paper. This encompasses underlying assumptions (e.g., about the adversary), methodological design (e.g., how an attack is mimicked by the data custodian), and the vulnerability reporting (e.g., the performance measurements). A metric is relative if it calculates vulnerability in relation to a baseline.
Privacy vulnerability<br>Privacy vulnerability is a measure of the likelihood of a privacy violation as a characteristic of the data.[44] This is distinct from "risk," which includes contextual factors such as the likelihood of an attack.[45]
Quasi-identifiers<br>Quasi-identifiers (QIs) are attributes in a dataset that are assumed to be known by an adversary (i.e., background knowledge). They are available to the adversary from public sources of information, because the adversary has access to non-public sources of information, and/or the adversary is an acquaintance of the target individual and has private background information about the individual.[38] Attributes that can be used to infer QIs are also considered QIs.[45]
Sensitive attributes<br>Sensitive attributes are the attributes that contain personal information about a target and are not considered to be direct identifiers or QIs. Typically, all remaining attributes are treated as sensitive attributes.[45]
Synthetic data generation<br>There are multiple ways that synthetic data can be generated. The term synthetic data generation (SDG) as used in this paper involves the training of a generative model on real data and the generation of fully synthetic data in the form of tabular individual-level data where one row corresponds to an individual.
Target<br>A target refers to a specific data subject or individual that the adversary is attempting to identify or infer personal information about. The attack or target dataset is the dataset of all targets.[46]
Threat modeling<br>Privacy metrics often mimic an attack by an adversary while utilizing the resources available to the data custodian. This requires making assumptions about the adversary, which is referred to as the threat modeling process.[38]

parts of the explanations into the statements themselves, without altering their meaning. However, some statements were omitted, while others were introduced (details are provided for each statement in Tables S1–S17).

Critically, each statement needed to be consistent in its meaning across at least two consecutive Delphi rounds as part of the stopping criterion. The stopping criterion was response stability, which was the lack of statistically significant (i.e., $p > 0.05$) group differences between two successive rounds as measured by the Wilcoxon matched-pairs signed rank test in line with other Delphi studies.[53,54] This was reached after the third round. The statements were then analyzed for consensus and agreement (see Figure 6 for how these criteria were defined). For most statements in the third round (10 of 11), the panelists' scorings indicated a consensus on agreement. These statements became the recommendations R1–R10 as presented in this paper. There was only 1 statement of 11 with consensus on uncertainty. All statements that were rated in the final round are given with their agreement levels in Table 2.

DISCUSSION

In this section, we explain the consensus recommendations and situate them within the broader landscape of privacy evaluation in synthetic data. Rather than separating results from discussion, we embed each recommendation from Table 2 within its relevant discussion topic, accompanied by context drawn from its statement evolution, from the panelists' feedback during the study process, from the literature, and from our critical analysis (also referred to as the detailed analysis report[47]). This integrated approach captures both the consensus recommendations and the underlying rationale that informed each recommendation.

The section is organized to reflect the steps in privacy evaluation, from threat modeling to specific disclosure concepts and to the interpretation of metrics and decision making.

Threat modeling

Privacy metrics are computed by the data custodian and are often based on simulating an attack by an adversary while utilizing the resources available to the data custodian. This requires making assumptions about the adversary as part of the threat modeling process.[38] However, privacy metrics in synthetic data often lack explicit threat models but make implicit assumptions through their design choices around the adversary's background knowledge, their motivations, constraints, and targets. These are discussed below.

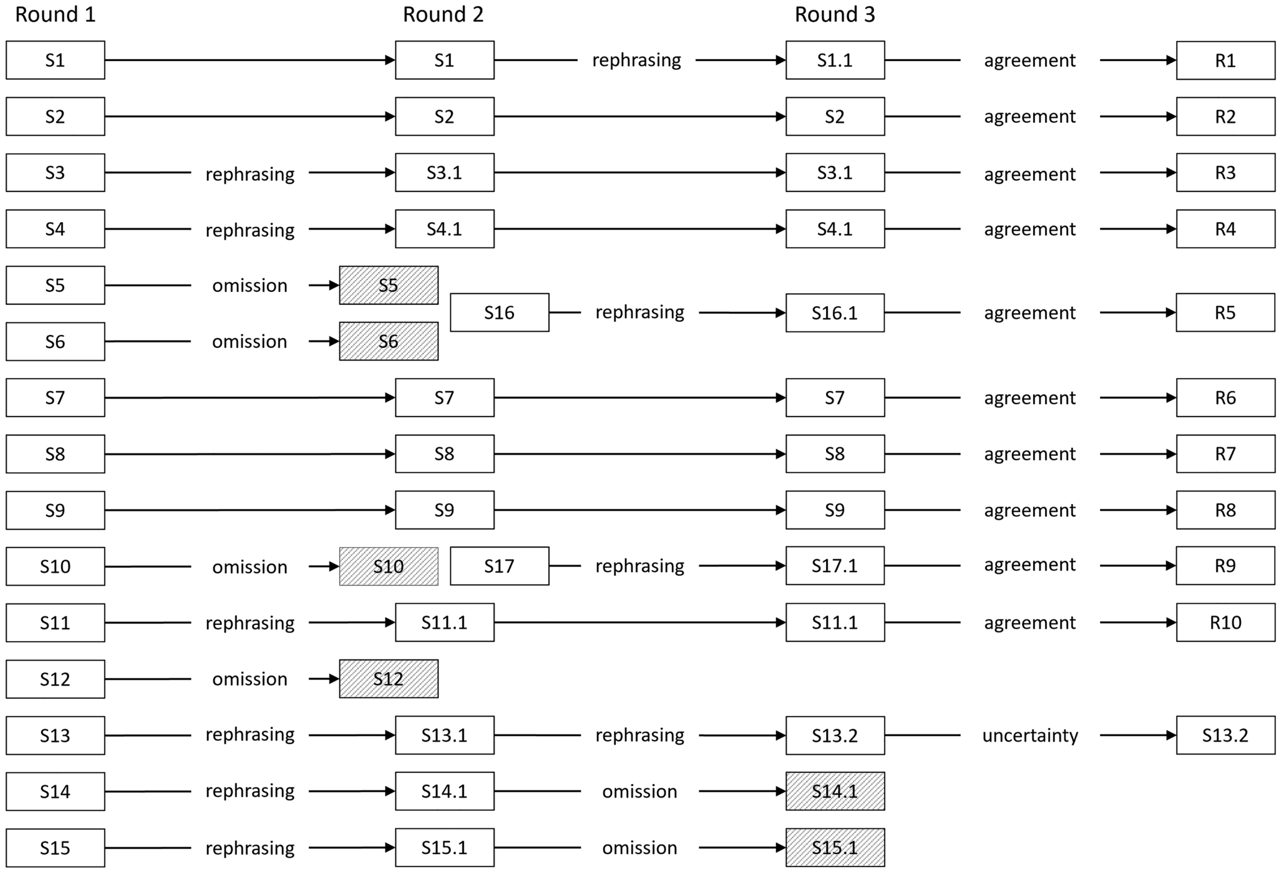

Figure 1. Statement evolution throughout the study

Statements were formulated in the literature-informed critical analysis (i.e., the report) and refined throughout the rounds based on the panelists' comments. Each statement was assigned with an identifier (e.g., S1), and version numbers (e.g., S1.1) were added in case of minor changes. The hatched statements are the ones that either underwent major changes resulting in a treatment as new statement or were omitted entirely. Details for each statement are provided in the supplemental information.

The adversary's background knowledge

Quasi-identifiers (QIs) are the *de facto* assumption of the adversary's background knowledge in line with the International Organization for Standardization (ISO) standard on ISO/IEC (International Electrotechnical Commission) 27559.[38] Many metrics base their calculation on entire records and thereby assume that all attributes are QIs and that this full set of attributes is leveraged by the adversary when attacking the synthetic data (see the report[47]). This assumption is rarely made explicit, yet the explicit documentation of QIs is necessary for interpreting the metric values.

A concern that is often raised in the context of QIs and was also expressed by some panelists (see the qualitative analysis in Table S1) is the subjectivity and inter-individual variability introduced by assumptions on the adversary's background knowledge. While this concern is valid, it should be noted that guidance for determining QIs has been published,[45,55] and the alternative of using all attributes is itself an assumption, namely that every attribute is known and utilized by the adversary. Some authors note that this would account for the worst-case scenario. However, it is also important to recognize that using all attributes does not necessarily result in the highest privacy vulnerability as calculated by the metrics. In the report, we conducted a simulation that illustrates how a metric for membership disclosure based on entire records did not result in a higher vulnerability than one based on a subset of attributes.[47] In fact, we observed that the more attributes were considered, the lower the estimated membership vulnerability on average. This observation was consistent across multiple datasets. A similar observation was made by Giomi et al.[56] when evaluating attribute disclosure. To properly explore a worst-case scenario, one would therefore need to evaluate all possible subsets of attributes and identify the subset with the largest vulnerability. This is rarely done in practice and would, in fact, quickly become computationally problematic.

By basing privacy metrics for synthetic data on carefully selected QIs, our recommendation R1 allows for a more realistic vulnerability estimate and ensures transparency around the assumptions underlying privacy evaluation.

Motivation, constraints, and targets

An adversary's motivation and constraints are further components of threat modeling that are often implicitly assumed in the calculations of privacy vulnerability. There are a variety of motivations that can drive an adversary[38,45,55,57–59] closely

CellPress
OPEN ACCESS

Table 2. Statements in the third (final) Delphi round

Number	Statement	Agreement, median (IQR)
Consensus on agreement		
R1	S1.1: disclosure vulnerability metrics should be based on quasi-identifiers. These may vary depending on the data context (e.g., can still be all attributes) and are ascertained by the data controller.	4 (0)
R2	S2: disclosure vulnerability metrics should not be calculated on a pre-selected subset of "vulnerable" records but for all of the records.	5 (1)
R3	S3.1: stand-alone similarity metrics (i.e., that are not part of the attribute or membership disclosure) should not be used to report privacy in synthetic data.	4 (1)
R4	S4.1: membership disclosure vulnerability should be evaluated only when the assumptions of the current metrics hold, which is that the adversary would learn something new for targets drawn from the same population as the training dataset.	4 (0)
R5	S16.1: because the F1 score, which is commonly used in membership disclosure metrics, is prevalence dependent, it needs to be reported relative to an adversary guessing membership.	5 (1)
R6	S7: meaningful attribute disclosure vulnerability applies only to individuals who are in the dataset (i.e., members). Penalizing accurate prediction on individuals who have not been part of the dataset (i.e., group privacy) requires a broader ethical framework.	5 (0)
R7	S8: a relative attribute disclosure vulnerability that takes a non-member baseline into account is meaningful.	5 (0)
R8	S9: in attribute disclosure vulnerability, a relative vulnerability higher than its threshold is only considered as unacceptably high when the absolute vulnerability is higher than its threshold.	4 (0)
R9	S17.1: the privacy budget ε is not an adequate metric to report disclosure vulnerability unless it is set to a value close to 0. Even when differential privacy methods are used, disclosure vulnerability would still need to be evaluated using the same metrics as those applied to non-differentially private synthetic data.	5 (1)
R10	S11.1: when evaluating a specific trained SDG model, disclosure vulnerability metrics need to be reported both for individual and multiple synthetic datasets (e.g., averaged across them and variation).	5 (1)
Consensus on uncertainty		
NA	S13.2: as an anchor for membership disclosure vulnerability, a relative F1 score vulnerability (i.e., F_{rel} value) of 0.2 is suggested.	3 (1)

These statements were rated by the panelists in the final round. Each statement was complemented by a brief explanation within the online tool and has a unique identifier with version numbers (e.g., S1.1), if applicable (see supplemental information). The Likert scale from 1 to 5 reflected the agreement level. For each statement, the median level of agreement across all panelists and its interquartile range (IQR) were calculated. Consensus was assumed with an IQR $\leq$1, and agreement with a median level of agreement >3. Consensus on agreement was achieved in 10 statements (i.e., IQR $\leq$1 and median level of agreement >3). These are considered to be recommendations and numbered R1–R10. One statement (S13.2) had a consensus on uncertainty (i.e., IQR $\leq$1 and median of 3) and cannot be seen as a recommendation.

linked to who their targets are likely to be but also how constrained they are.

In this context, we observed that privacy vulnerability is often calculated for a pre-selected subset of targets.[56,60–62] The selected targets are then labeled as "vulnerable" targets.[60,61,63] For example, "members of minorities" have been used as selected targets.[60] The idea is, again, to account for the worst-case scenario, assuming that these are the ones experiencing the maximum disclosure vulnerability. Such an *a priori* assumption is, however, not necessarily true.[56,62] A minority (or rare) record may be rare in its QIs—thereby more vulnerable to identity disclosure—yet have common or weakly correlated sensitive attributes, making it less vulnerable to attribute disclosure. It depends on the dataset, its correlational structure, and the vulnerability being examined. Consequently, our consensus recommendation R2 is to not pre-select a subset of "vulnerable" records. Estimating the maximum disclosure vulnerability across a synthetic dataset always involves calculating vulnerability for

each record in the first instance. Limiting the evaluation to a pre-selected subset introduces selection bias and may overlook targets with higher vulnerability than the selected ones.

Identity disclosure

Identity disclosure occurs when an individual's identity can be assigned to a record (see Table 1). SDG generates data that reflects the statistical properties of the training dataset without preserving any direct link between a synthetic and a real record. This means SDG should protect against identity disclosure by design. However, SDG models can, for example, overfit and thereby reveal an identity so that identity disclosure vulnerability may still be relevant in synthetic data. In the following, we discuss two approaches that have been used to approximate identity disclosure: record-level similarity and replicated uniques.

Record-level similarity

Record-level similarity metrics are the most prevalent metrics to measure privacy in synthetic data.[32] They assess the distance between the training and synthetic data. This distance can serve as a metric by itself,[64,65] meaning that closeness is then considered as an indicator of high vulnerability. It can also be compared against a baseline.[49,66,67] Record-level similarity metrics, however, fail to adequately account for identity disclosure in synthetic data for three main reasons. First, similarity between a synthetic and a training record does not necessarily imply privacy vulnerability.[66,68] If the identity disclosure vulnerability of the training record is very small, then similarity would not necessarily indicate elevated disclosure vulnerability. Second, the metrics typically do not account for scenarios where the same synthetic record is closest to multiple training records, which may reflect a different vulnerability than being closest to one record. Third, similarity based on QIs does not imply similarity in sensitive attributes. If the sensitive attributes of the synthetic record differ from those in similar training records, then an identity disclosure claim may not be meaningful. In light of these main limitations, the consensus recommendation R3 discourages the use of record-level similarity as stand-alone metrics.

Replicated uniques

A specific way to measure identity disclosure is through uniqueness.[45] For synthetic data, metrics derived from replicated uniques (i.e., records that are unique in the training data and replicated in the synthetic data) have been proposed as identity disclosure metrics.[56,69] We did not consider replicated uniques in the report and the consensus study, since they were not mentioned[32,48,50] or mentioned but classified as record-level similarity[49] in the underlying systematic reviews. Nevertheless, we want to highlight some of their characteristics. One challenge is a recurrent topic in the privacy literature, which is that a unique record in a dataset may or may not be unique in the population.[45,68,70] This is relevant in situations where the training data are drawn from a larger population. A holdout dataset, as proposed by Giomi et al.,[56] is unlikely to adequately account for the population, as results heavily depend on how much of the population is captured in this holdout dataset (i.e., its size). Also, the metrics' interpretation can be difficult when synthetic records that are identified as replicated uniques in terms of their QIs differ in sensitive attributes from the training records. This comes back to the issue of meaningful identity disclosure claims as discussed in the context of record-level similarity. Importantly, vulnerability can still be unacceptably high for non-unique records. Thus, uniqueness metrics can only provide a lower bound in terms of identity disclosure vulnerability.

Membership disclosure

Membership inference is a classification task with the labels being a member of the training dataset versus being a non-member. There are two main approaches in the literature to calculate membership disclosure vulnerability in synthetic data: one is to mimic an adversary who matches targets from an attack dataset to synthetic records (i.e., partitioning methods) and would give a membership guess when there is a match,[43,67,71–78] and the other is to mimic an adversary who trains a classifier, typically with the support of shadow models, and would give a membership guess when the target is classified as "member" by this model.[49,60,62,79–84] The main concern with current metrics for both approaches is their internal inconsistency of assumptions, whereby components of conflicting threat models are simultaneously leveraged.

Assumptions in narrative and metric

As mentioned previously, the definition of potential targets is a relevant component of threat modeling. The implicit or explicit assumption of current metrics for membership disclosure is that the adversary draws targets from the same population the training dataset is sampled from.[43,60,62,72,75–77,79–91] The attack dataset includes some records that are part of the training data (i.e., members) and some that are not (i.e., non-members), but all records are drawn from the same population the training dataset was sampled from. If this population is, for example, individuals with HIV, then all targets in the adversary's attack dataset are HIV^+ and membership status does not reveal this characteristic to the adversary because it is already known to the adversary. We call this scenario A (see Figure 2A).

At the same time, an often-used narrative for calculating membership disclosure vulnerability is the scenario where an adversary infers information about the population (e.g., individuals with HIV) through membership disclosure (e.g., in an HIV training dataset).[62,75,88,91] In this scenario, referred to as scenario B (see Figure 2B), targets must be drawn from a different population (e.g., individuals with and without HIV). Unlike scenario A, targets are then HIV^+ or HIV^-, and the membership status can reveal the positive HIV status to the adversary.

Current metrics for membership disclosure vulnerability operationalize scenario A while telling the narrative of scenario B, meaning that the threat model is not consistent.[62,75,88,91] These two scenarios, however, differ in the number of members that are included among the adversary's targets, meaning that the maximum possible success rate of the adversary can vary. This variation can be substantial, as shown in an example simulated attack included in our detailed report.[47]

This means that using metrics that operationalize scenario A to report on scenario B can be misleading. This is reflected in the consensus recommendation R4, which calls for aligning the membership disclosure metric's assumptions with the specific threat model under investigation.

Prevalence-aware interpretation: A naive membership guess

Membership disclosure is a binary classification task, and another concern relates to the use of classification performance

CellPress
OPEN ACCESS

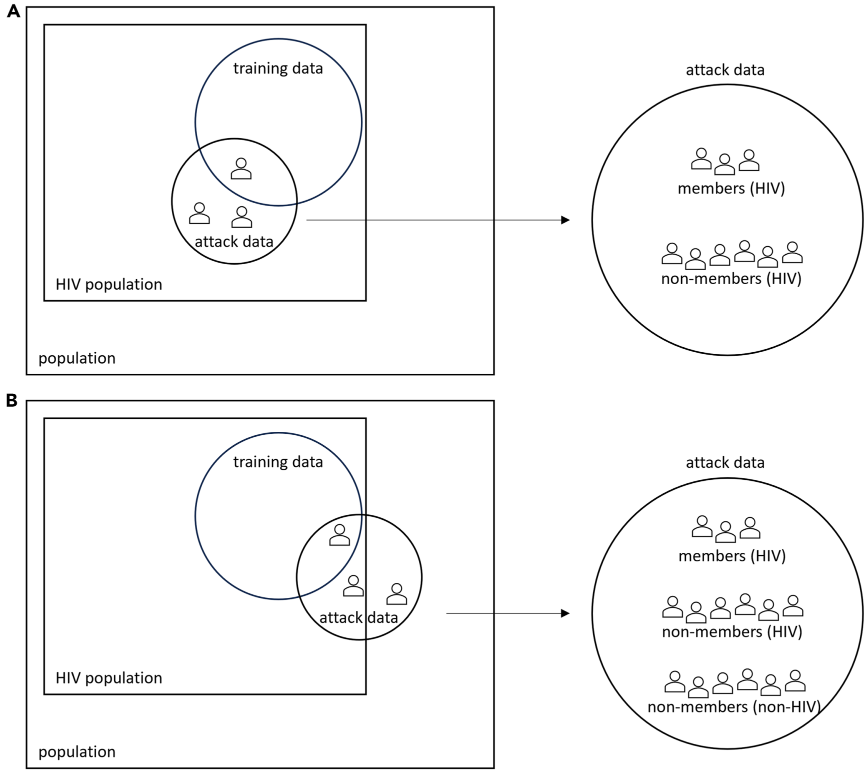

Figure 2. Different adversary's attack datasets

In these scenarios, the training (original) dataset consists of people with HIV. It is randomly drawn from an HIV population. In scenario (A), the attack dataset is randomly drawn from the same population as the training data (e.g., young people with HIV in Ottawa, Canada). This means all targets are individuals with HIV. In scenario (B), the attack dataset is randomly drawn from a superpopulation (e.g., all young people in Ottawa) and contains targets with and without HIV. In scenario (B), membership status can reveal the HIV status to the adversary.

measurements that build upon the confusion matrix (e.g., precision, recall, or F1 score) without acknowledging their sensitivity to prevalence.[72,75–77,79,81,82,89] Most membership disclosure metrics build an attack dataset that includes 50% members (i.e., prevalence)[72,75–77,79,82,85] or use another arbitrary fixed value.[71,81,86] If, however, targets are drawn from the same population that the training dataset is sampled from (i.e., scenario A), then the expected member prevalence is the sampling fraction of the training dataset.[43]

A prevalence-aware baseline can then ensure meaningful interpretation of confusion matrix-based metrics such as the F1 score. This can be an adversary who guesses membership without leveraging the synthetic data (i.e., naive baseline) as proposed by El Emam et al.[43] The naive baseline would be 1.0 when the training dataset is identical with the population (i.e., sampling fraction of 1) and gets close to zero the smaller the sampling fraction. The F1 score can be standardized by this baseline and is then a relative metric (i.e., Frel) given the incremental vulnerability introduced by the synthetic data. This approach is affirmed in our consensus recommendation R5. It is important to note that this is a specific recommendation tied to prevalence-dependent metrics, which are commonly used in practice. Other metrics that report, for example, the area under the receiver operating curve (AUROC) offer a more universal interpretation, with 0.5 corresponding to the naive membership guess, meaning that an explicit adjustment would not be required. The evolution of related consensus statements (see Tables S5–S7 and S14) reflects the challenge in interpreting membership disclosure metrics with respect to the chosen performance measurement.

These discussions in the context of membership disclosure triggered a broader question of performance measurements throughout the study process. A performance measurement can implicitly reflect the motivations and constraints of an adversary as it may ignore incorrect (i.e., false positive) or missed (i.e., false negative) disclosure claims. This influences the final vulnerability estimate and its interpretation. Although performance measurement is an integral component of a privacy metric, this aspect of design choice (e.g., AUROC versus F1 score) remains largely unexplored in the privacy literature. For example, the F1 score is, as mentioned, commonly used to report membership disclosure vulnerability.[43,75,79] It uses recall and precision to describe classification performance and ignores the portion of true negatives.[92] While this seems reasonable in the case of membership disclosure, where a consideration of true negatives could inflate the vulnerability value, the equal weighting of precision and recall should be informed by the threat model: if false positives are costly, then precision must have more weight than recall (e.g., F0.5 score). In attribute disclosure vulnerability (see below), the situation is even more complex: the prediction task can be categorical (i.e., classification) or continuous (i.e., regression). For example, with a binary sensitive attribute (i.e., binary classification), an adversary can be interested as much in true negatives as in true positives. Then, precision, recall, or the F1 score are not a good choice. Also, performance metrics derived from the confusion matrix (e.g., precision, recall, or the F1 score) rely on probability thresholds and are sensitive to class prevalence,[92–94] further complicating their interpretation in privacy evaluation. Other performance metrics like the AUROC are threshold independent but do not account for the associated costs that come with a specified threat model. As the space of performance measurements is large, and the implications of each choice have yet to be examined, it was not part of our consensus process. However, we identified it as a relevant gap and potential direction for future research so that recommendations can eventually be formulated.

Attribute disclosure

Attribute inference can be viewed as a prediction task in which an adversary uses their background knowledge (i.e., QIs) to

predict a sensitive attribute for their target. They leverage the synthetic data to train the prediction model. In the literature, a multitude of models have been used to solve this task,[56,60,61,63,95,96] where the model's prediction performance on the targets is commonly interpreted as a measure of attribute disclosure vulnerability.

The scope of attribute disclosure vulnerability

A fundamental challenge in attribute disclosure that we identified is that inferring a sensitive attribute is not a privacy violation per se. In fact, such inferences can occur even in the absence of any data being disclosed or without the target being part of a disclosed dataset (i.e., non-member). Model training and prediction are common tasks in scientific investigations, whether they are academic or commercial, and accurate predictions are the very aim of such investigations. The commonly used practice of interpreting high prediction accuracy as a privacy violation[72,76,96,97] implies that we should not retain important relationships in synthetic data. However, these relationships are important for generating generalizable knowledge about the underlying population. Such knowledge is very valuable, for example, in preventive medicine, where it can reduce mortality and improve health outcomes. However, it can also lead to harm, particularly when sensitive or stigmatizing patterns are revealed. In response, some authors have proposed that the concept of privacy can be extended to collective or group privacy in the context of big data.[98,99]

From a technical perspective, however, group privacy is primarily concerned about the harm that results from data release and goes beyond what privacy metrics are designed to capture or mitigate. This is not to say that the release of data, its analysis, and knowledge generation cannot be harmful to non-members. On the contrary, fair and responsible data release and use requires ethical considerations beyond individual privacy, addressing potential collective and group harms. This perspective is also acknowledged in regulatory guidelines on SDG.[100–102] Such considerations are consequently relevant but extend beyond what privacy metrics are aiming at (and are capable of). Reflecting this distinction, our consensus recommendation R6 states that meaningful attribute disclosure vulnerability applies only to individuals who are part of the dataset (i.e., members). Penalizing accurate prediction on individuals who have not been part of the dataset (i.e., group privacy) requires a broader ethical framework.

Knowledge generation: A non-member baseline

A major challenge that follows is to disentangle attribute inference that occurs from being part of a dataset from the inference due to being part of the population where the dataset is drawn from.[44,68,103–105] The idea is to make sure that being a member in a dataset does not increase the likelihood of an adversary gaining sensitive information about an individual such that everything that can be learned about the individual can also be learned without them being a member of the training dataset. To make that distinction, metrics have been proposed that quantify how being part of the dataset affects the correct inference of attributes about an individual and assign this value as a measure of disclosure (e.g., the metric proposed in Taub et al.[61] in the "differential confidentiality" notion or the metric in Stadler et al.[60]). However, a more computationally efficient approach is to establish a non-member baseline and compare this baseline to the information gained about members (e.g., Francis and Wagner and Giomi et al.[44,56]). The incremental prediction performance is then interpreted as a measure of attribute disclosure, which provides a meaningful interpretation as affirmed in our consensus recommendation R7.

Interpretation beyond random guessing

The incremental or relative attribute disclosure vulnerability compared to a non-member baseline is not sufficient to guide decision-making processes since the accuracy of the learned information (i.e., absolute prediction accuracy) matters from the perspective of both the adversary and the target individuals. Therefore, it is not only the difference from the non-member baseline, but also where the difference is within the range of the scale matters. It may, for example, be acceptable that there is a difference in prediction performance in cases where the accuracy of the learned information remains low and is no better or even worse than a random guess.

Consider a simple example where the sensitive attribute is binary (e.g., diagnosis or no diagnosis). If the AUROC is 0.4 for members and 0.1 for non-members, then the difference between them is arguably large but both values are worse than a random guess. The absolute values, in this case, indicate that this is not an attribute disclosure. However, if the member AUROC was 0.9 and the non-member AUROC was 0.6, the difference is the same, but the high member AUROC would suggest that an adversary learns the diagnosis with high accuracy. Our consensus recommendation R8 emphasizes this aspect. Metrics may use other performance measurements than AUROC, but the same principles apply: attribute disclosure is meaningful only when predictions outperform both a non-member baseline and a random guess.

DP

DP is a framework that can be and has been applied to SDG.[22,106–108] In DP-SDG, the parameter ε (or privacy budget) is typically interpreted as a measure of privacy. This parameter, or more precisely e^{ε}, is a relative quantification of how much the results of a mechanism—in our case, SDG—are allowed to differ when one record is changed in the dataset. This means that the privacy budget translates exponentially into changes in the results. With a budget close to 0, analytical output from the data hardly changes regardless of whether any particular individual is in the data. Such a small privacy budget can give the mathematical guarantee that privacy is preserved.[109] If the privacy budget becomes large, however, theoretical privacy presumptions cannot be easily translated into empirical privacy.[110] More broadly, the interpretation of the privacy budget is tied to its unit of privacy[103] and is likely to depend on the implementation.[111] This leads to the question of what should be considered a small ε and whether there exists any ε other than 0 with a clear privacy interpretation. Definitions of small ε largely vary across academia. For example, Muralidhar et al. make use of an ε of 1.0,[111] Stadler et al. implement an ε of 0.1,[60] Li et al. state that 4 is an empirically reasonable value,[86] Rosenblatt et al. consider an $\varepsilon < 3.0$ as low,[108] and Hayes et al. report an $\varepsilon < 10$ as acceptable.[81] Industrial and government applications also have ε values that vary from 0.1 (e.g., Rogers et al.[112]) to above 18 (e.g., Abowd et al.[113]), and the just recently published Guidelines for Evaluating Differential Privacy Guarantees by the US National Institute

CellPress
OPEN ACCESS

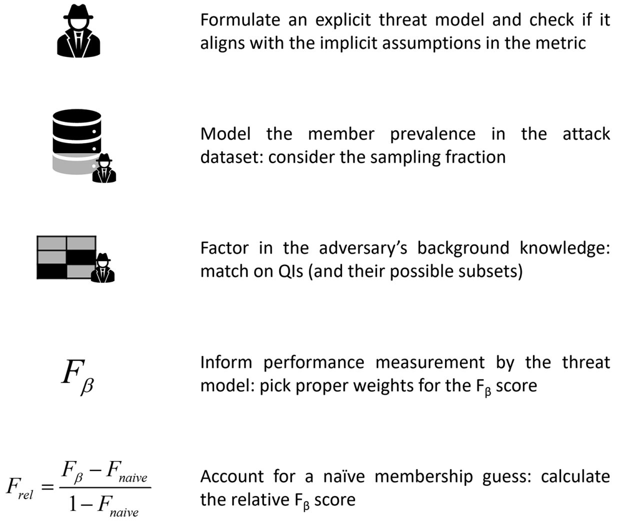

Figure 3. Practical guidance to calculate membership disclosure vulnerability

Steps to avoid common pitfalls and enhance the proper use of commonly used membership disclosure metrics (e.g., in El Emam et al. and Yan et al.[43,71]).

of Standards and Technology acknowledges that setting the parameter ε remains an open question.[114] Given the current lack of interpretability of large ε values, the consensus recommendation R9 highlights that privacy in terms of membership and attribute disclosure still needs to be evaluated empirically (unless ε is set to a value close to 0). Such an empirical privacy evaluation in DP-SDG has, for example, be done in Stadler et al., [60] Abowd et al.,[104] and Adams et al.[115]

Metric interpretation and decision-making

Privacy metrics are ultimately meant to inform decisions. These decisions may involve benchmarking or optimizing SDG models or making binary release decisions for one or multiple synthetic datasets. Each type of decision requires different considerations about how metrics are applied and interpreted.

Stochasticity of the process

SDG is a generative process with stochastic variability in its output. Stochasticity has been shown to be relevant in the utility evaluation of synthetic data,[116] where it has been recommended to average across 10 synthetic datasets to reach a plateau.[117] However, the impact of stochasticity is not limited to utility. Our consensus recommendation R10 highlights that when evaluating the vulnerability of a trained SDG model rather than a synthetic dataset, it is more appropriate to report the aggregate vulnerability (average and standard deviation) across multiple synthetic datasets from the same model. However, if the decision-making scenario is data release, then the disclosure vulnerability for the specific synthetic dataset(s) may be the most relevant. Given that it is not always possible to determine *a priori* the exact decision-making scenario, it can be prudent to have both types of results.

Thresholds

Whether a privacy vulnerability metric is absolute or relative, whether it is the F1 score, the AUROC or another performance measurement, having a threshold (or anchor value) to compare against is necessary to interpret the metric and for decision-making. This is particularly true for binary release decisions where thresholds are needed to determine whether a vulnerability metric's value is too high or acceptable. Such a value depends on the context and needs to be informed by the sensitivity of the data, potential harm, and appropriateness of consent and notice.

A widely adopted approach to set thresholds is to rely on precedents. For example, precedents have been used to set thresholds for (absolute) identity disclosure vulnerability with anonymized data.[38,118,119] Similarly, the Singapore regulatory guidelines on SDG present precedents from identity disclosure, even while acknowledging that such values may not be directly applicable to vulnerabilities in synthetic data.[100]

In membership disclosure, a value of 0.2 has been used to evaluate the relative Frel for membership disclosure vulnerability by some authors.[14,43,73] Whether or not this should be a recommendation remained uncertain in our study (see statement S13.2 in Table 2).

In attribute disclosure, there were no thresholds established in the literature, but it is commonly understood that models are generally not capable of performing as well on unseen data as they do on the training data.[120–122] Consequently, it is likely that a difference from the non-member baseline (i.e., unseen data) will always remain,[56,60] and an acceptable deviation must be agreed on. Considerations for deriving such a threshold may be based on experiences with performance measurements more generally (see an example for AUROC in the report[47]). While we included thresholds based on these considerations as initial statements in the Delphi study, we ultimately omitted them, acknowledging that the supporting evidence was not directly related to privacy (see Tables S16 and S17). In general, more empirical precedents are needed, especially given the large space of performance measurements. This is a relevant area for future research with significant practical implications.

Overall summary

The aim of our study was to establish standardized practices to evaluate privacy in synthetic data through recommendations based on a critical analysis of privacy metrics used in the literature and agreed upon by experts in a formal consensus process. Membership disclosure and attribute disclosure vulnerability were identified to be the most suitable for evaluating privacy in synthetic data while the use of record-level similarity was discouraged. Also, DP synthetic datasets would require the same privacy evaluation as non-DP datasets (if ε was not close to zero).

Most published metrics on membership and attribute disclosure either rely on assumptions that must be validated for the specific use case, are not interpretable by themselves, or make use of an inappropriate baseline. As a practical guidance, the first step in applying any privacy metric in synthetic data should therefore be an explicit threat model. This model can inform metric choice and configuration of parameters.

The need for threat modeling became particularly relevant in membership disclosure where current metrics implicitly or explicitly cover one specific threat model that may not correspond to the one under evaluation. Only if the threat models align are current metrics able to give a realistic estimate for vulnerability. Figure 3 offers practical steps to avoid common pitfalls and improve the use and interpretation of commonly used

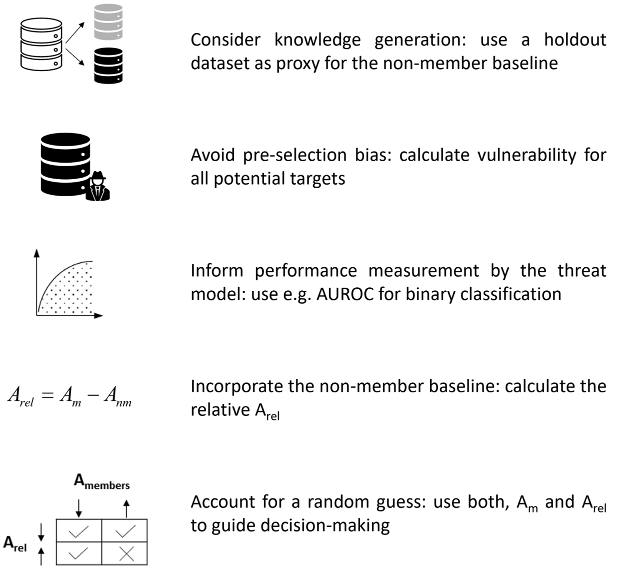

Figure 4. Practical guidance to calculate attribute disclosure vulnerability

Steps to avoid common pitfalls and enhance the proper use of current attribute disclosure metrics (e.g., Giomi et al. and Taub et al.[56,61]). A, performance measurement (i.e., AUROC); m, members; nm, non-members.

membership disclosure metrics (e.g., the metric by El Emam et al.[43] or Yan et al.[71]).

Metrics that quantify attribute disclosure vulnerability come with the challenge of disentangling attribute inference that arises from being part of a dataset from that which arises from being part of the population where the dataset is drawn from. Figure 4 provides a practical solution to this challenge and offers further steps to improve the use and interpretation of current attribute disclosure metrics (e.g., the metrics by Giomi et al.[56] or Taub et al.[61]).

Future work

Our recommendations are based on the practical reality of how privacy metrics are commonly used and how their application can be improved. As highlighted throughout this study, there are specific opportunities for further research to improve the framework. Crucial points that need to be addressed are as follows.

(1) Adversarial strategies. In synthetic data, an adversary can be motivated to leverage subsets of QIs or use generalized attributes—such as reducing a 3-digit diagnosis code to its 2-digit parent—instead of relying on the complete set. A comprehensive analysis of different strategies is needed to account for worst-case scenarios.
(2) Performance measurements. The choice of performance measurement can considerably influence the final vulnerability estimate. This has not been examined in the literature. Also, current metrics of attribute disclosure often focus on classification, but regression is another relevant task in attribute disclosure and should be explicitly addressed.
(3) Thresholds. Precedents can provide a valuable resource to inform thresholds and thereby facilitate the wide adoption of SDG. Future research and experience with synthetic data can inform the choice of threshold values.
(4) Identity disclosure metrics. Identity disclosure can be strictly interpreted as establishing a link between a record and a real identity. There is, however, currently no metric available that provides a meaningful application for synthetic data.
(5) Membership disclosure metrics. There is a gap in current membership disclosure metrics in terms of providing correct vulnerability estimates for different threat models.
(6) Attribute disclosure metrics. There are many ways to model prediction tasks and to quantify their prediction performance. Standardizing prediction models would provide results that can be comparable across studies.

Furthermore, the current framework should be operationalized and implemented in practical settings to gain experience with its strengths and weaknesses.

Limitations of the study

There are several limitations to this study that we wish to highlight. While our Delphi design is justified and based on respective guidance, we cannot ultimately exclude that there was group bias in our study or forced consensus (due to a misconception by some panelists that the stopping criterion was related to consensus). Also, qualitative results may vary depending on the researcher who carries out the analysis.[123] In this sense, we cannot exclude that there may have been further key topics that have not been identified but could have prompted refinement of the statements or the report. Also, the qualitative analysis informed the rephrasing, omission, or introduction of statements. Under an ideal process (as noted by the RAND guidelines), the statements should not be adjusted to comply with the iteration criterion.[124] It is, however, common practice in modified designs, and the incorporation of the panel's feedback is mentioned as an integral component of the consensus-building process according to other guidance.[125,126]

We do not recommend specific privacy metrics (i.e., implementations) but good practices for realistically calculating vulnerability. For all reviewed metrics, we identified challenges and drawbacks that are provided in detail in the report (see the report[47]). The present paper helps to improve metrics that are currently in use, but the development of metrics was outside the scope of the study. Real-world case studies based on improved metrics are therefore left for future research. We also did not address the question of how the SDG process can be optimized to mitigate disclosure.

Finally, other modalities such as image or text were not considered. While our recommendations for good practice very likely hold for these modalities as well, quantification of disclosure vulnerability can differ in material ways so that further considerations apply.

METHODS

Study process

The typical process to achieve expert agreement involves a literature review, followed by a report and a formal consensus method. Such processes have been used widely in health

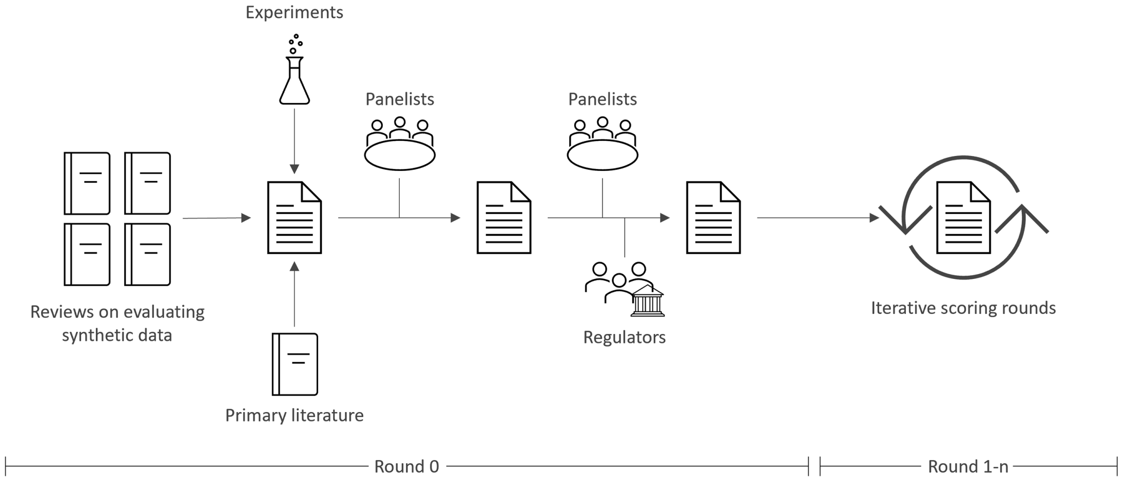

Figure 5. Study process

Four literature reviews on the evaluation of synthetic data served as a starting point to identify commonly used privacy metrics.[32,48–50] Their primary literature was reviewed, and various additional simulation experiments conducted to better understand their behavior. Panelists reviewed and revised the report (round 0), and regulators were invited to comment on the report. Statements were then created from the report's conclusions and rated by the panelists in Delphi rounds (round 1-n).

research, for example, to develop guidelines or to decide on important fields of research.[124,126–130] As a formal consensus method, we conducted a Delphi process as it aims at free choice while preventing personal bias and dominance (''halo effect''). We applied a modified Delphi design as follows.

(1) Round 0: develop a report on privacy metrics that is reviewed by the panelists, formulating most relevant findings into clear statements (i.e., proposed recommendations).
(2) Round 1: scoring the statements generated in round 0.
(3) Rounds 2-n: re-scoring the statement after controlled feedback with a minimum of $n = 2$ until the stopping criterion is met.

This process followed the respective guidelines[124–129,131] and is depicted in Figure 5.

The overall study process lasted from February 2024 until November 2024. Three scoring rounds were required to meet the stopping criterion. The participation rate was 100% in all Delphi rounds.

Expert panel

The choice of the panel is known to heavily impact a consensus study's results.[124,132] For this study, an expert panel of 13 individuals was set up. It has been argued that panelists should reflect the diversity of the topic.[124] While privacy indeed is a multidisciplinary topic, the purpose of this consensus study was to evaluate existing privacy metrics for synthetic data from a technical perspective. Consequently, the panelists were expected to have a high level of technical expertise in privacy metrics to understand, discuss, and give opinions from a technical perspective. Consistent with that scope, we did not include laypeople or other professions such as members of ethics committees.

We used a mixed recruitment strategy for the panel.[124] Identification of experts can be done, for example, through objective criteria such as a literature review or subjective criteria such as a colleague's recommendation. In this study, the following criteria were used to select experts for the panel: editorial board member of *Transactions on Data Privacy* during the period 2019–2024 and consistent conference committee membership of Privacy in Statistical Databases during the period 2019–2024. The journal *Transactions on Data Privacy* was chosen due to its outstanding role in communicating high-quality findings in data privacy technologies. Privacy in Statistical Databases is a key conference attracting a global audience in the field of data privacy. It was sponsored by the United Nations Educational, Scientific, and Cultural Organization Chair in Data Privacy. Both resources are very focused on technical privacy topics, and their members are representative of the respective research community. We identified 11 experts according to this criterion and invited them by e-mail to participate in the panel. We extended this approach by including recommended experts. These were nominated by the initially identified experts or by the study's coordinators. For these additional nominated experts, we confirmed that they have published scholarly work relevant to our topic in the last 5 years. In this way, an additional 9 experts were identified and invited to participate. Of those 20 experts who were identified, 13 responded positively to the invitation to participate in this study. This is within the range of typical panel sizes reported in the literature.[124,133]

Literature-informed critical analysis (round 0)

The literature-informed critical analysis focused on ways to assess privacy vulnerability in synthetic data. Instead of conducting yet another systematic review on privacy metrics in synthetic data in round 0, we used four recently published reviews on the evaluation of synthetic data[32,48–50] and conducted a critical analysis built upon their findings. This critical analysis is provided as a report on OSF.[47] In the report, four categories (i.e., record-level similarity, membership disclosure, attribute disclosure, DP) were defined, illustrated through exemplar metrics, and this was followed by a critical appraisal.

As suggested by Fitch et al.,[125] we included broad feedback into round 0. This was collected from the panelists as well as from experts from six privacy and health regulators that have done work on synthetic data privacy. These experts were from Canada, Italy, Singapore, South Korea, the United Kingdom and the United States. They did not participate in the Delphi rounds, and their views did not represent their agency or imply endorsement.

The report was used to identify the most relevant questions on privacy metrics in synthetic data, formulated as statements (i.e., proposed recommendations), and served as background material for the panelists during the entire study process (available on OSF[47]).

Scoring rounds and analysis

In the scoring rounds, panelists indicated their level of agreement to the statements on a five-point Likert scale, which is commonly used in Delphi studies.[132] Comments could be provided to give explanations for the indicated level of agreement. The scoring rounds were conducted online using the Welphi software and were pilot tested within the coordinator's research lab beforehand. Throughout all Delphi rounds anonymity was maintained.

After each round, responses were analyzed both quantitatively and qualitatively. Relative frequency distributions, median, and interquartile range (IQR) were calculated. Panelists received a personalized statistical summary with the relative frequency distribution of responses of the previous round alongside their own previous response.[124] Comments were analyzed with two objectives. The first objective was to refine the statements and the report in between the rounds, and the second was to identify counterarguments. Details of the qualitative analysis and its results are given in Tables S1–S17.

Stopping criterion

The stopping criterion was defined *a priori* as group stability. We did not use consensus as the stopping criterion to avoid forced consensus and to account for scenarios where plurality (i.e., no consensus) might be a stable outcome.[53,126,134] We therefore checked for significant group differences between two successive rounds by the Wilcoxon matched-pairs signed rank test consistent with other Delphi studies.[53,54] Stability was then defined as $p > 0.05$. As soon as all statements achieved stability, no further round of re-scoring was initiated.[124]

Consensus measurement

Consensus in the literature has been defined in various ways, with criticisms highlighting that multiple definitions fail to distinguish between stability, consensus, and agreement.[53,126,132,135,136] In our study, consensus was measured after stability was achieved and included both determining (1) whether a consensus exists and (2) whether agreement was observed. The IQR has been proposed as an objective way to determine whether consensus is achieved.[53] Consensus was defined as a maximum 1.0-point range of the IQR and non-consensus (i.e., stable plurality) as a more than 1.0-point range.[53] In those statements where consensus was achieved, the median was used to determine the type of consensus (i.e., agreement, uncertainty, or disagreement).[125] The agreement definition in Fitch et al.[125] was adapted to the five-point Likert scale as shown in Figure 6. All methods were defined *a priori*.

Evolution of statements

With the panelists' feedback, statements were refined during the rounds. This is depicted for each statement in detail in Tables S1–S17. Figure 1 illustrates how the statements evolved. The identifier (e.g., S1) serves as a unique label for each statement throughout the study. When a statement was rephrased without a change in meaning (i.e., a minor change), it retained its original label with a version number added (e.g., S1 becomes S1.1). If the meaning of the statement changed substantially (i.e., a major change), then it was treated as a new statement and assigned a new identifier.

Importantly, each statement needed to be consistent in its meaning across at least two consecutive rounds as part of the stopping criterion. This means that, for example, statements could only undergo minor adjustments to be considered in the stability assessment. Substantially changed statements were treated as newly introduced statements that would require another round as part of the stopping criterion.

RESOURCE AVAILABILITY

Lead contact

Requests for further information and resources should be directed to and will be fulfilled by the lead contact, Khaled El Emam (kelemam@ehealthinformation.ca).

Materials availability

This study did not generate new unique reagents.

Data and code availability

We used publicly available as well as confidential data for the simulations in the report.[47] The data sources are listed alongside the simulations on OSF: https://doi.org/10.17605/OSF.IO/QAHUV,[137] and access can be requested directly at the source. The lead contact can be contacted for further information or support to gain access. All original code for our simulations has been deposited on OSF[137] as of the date of publication.

ACKNOWLEDGMENTS

We want to thank the privacy and health regulators from Canada, Italy, Singapore, South Korea, the United Kingdom, and the United States for their input on the critical analysis report that was developed as part of this research. Their views did not represent their agency or imply endorsement. We also want to thank Karen Otte (Berlin Institute of Health at Charité – Universitätsmedizin Berlin, Medical Informatics Group, Berlin, Germany) for independently reviewing the manuscript and providing valuable feedback during the revision process. This work is funded by the Canadian Institute for Advanced Research (CIFAR) and the Bill & Melinda Gates Foundation. K.E.E. is funded by a Discovery Grant RGPIN-2022-04811 from the Natural Sciences and Engineering Research Council of Canada and the Canada Research Chairs program from the Canadian Institutes of Health Research. L.P. is funded by the Deutsche Forschungsgemeinschaft (DFG, German Research Foundation) (grant no. 530282197). J.D. receives funding from the US Census Bureau for conducting research on formal privacy methods for survey data (corporate agreement CB20ADR016000). J.D.-F. is funded by the Government of Catalonia (ICREA Acadèmia Prize) and the European Union's NextGenerationEU/PRTR via INCIBE (project "HERMES" and INCIBE-URV cybersecurity chair). B.M. receives funding from the National Institutes of Health grant nos. RM1HG009034, U54HG012510, and K99LM014428. M.E. receives funding from the UK Research and Innovation (UKRI) grant ES/Z502984/1. L.K. was partially supported by grants from the CIFAR AI Chairs program, the Alberta Machine Intelligence Institute (AMII), the Natural Sciences and Engineering Council of Canada (NSERC), and the Canada Research Chair program from NSERC. J.L.R. is partially funded by the SYNTHIA project, an IHIJU under grant no. 101172872.

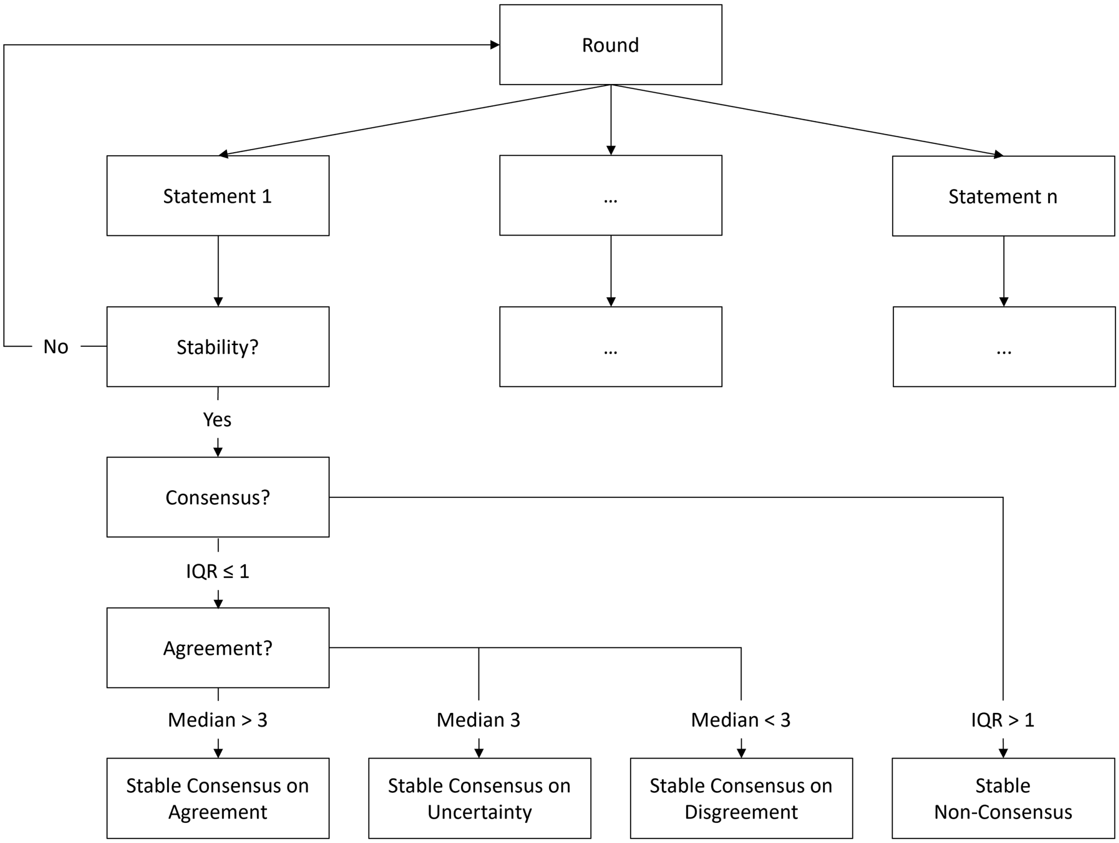

Figure 6. Consensus measurement
Consensus was measured after the stability of all statements was achieved. The measurement included two steps: (1) whether consensus is achieved and (2) whether agreement is achieved. Four possible outcomes could be expected. IQR, interquartile range.

AUTHOR CONTRIBUTIONS

Conceptualization and design: L.P. and K.E.E.; analysis, simulations, and drafting of the initial version of the report: L.P.; reviewing & revising the report: L.P., F.K.D., J.D., M.E., K.E.E., J.D.-F., P.F., M.K., L.K., B.M., K.M., P.M., F.P., J.L.R., and C.Y.; panelists in the Delphi study: F.K.D., J.D., M.E., K.E.E., J. D.-F., P.F., M.K., B.M., K.M., P.M., F.P., J.L.R., and C.Y.; drafting the manuscript: L.P. and K.E.E.; manuscript review & editing: L.P., F.K.D., J.D., M.E., K.E.E., J.D.-F., P.F., M.K., L.K., B.M., K.M., P.M., F.P., J.L.R., and C.Y.

DECLARATION OF INTERESTS

K.E.E. is the scholar-in-residence at the Office of the Information and Privacy Commissioner of Ontario and owns shares in Aetion Inc., which acquired his university spinoff that develops synthetic data software. P.M. has led on the research and development of synthetic data that are made available for researchers for a licensing fee by the CPRD, which is the Medicines and Healthcare Products Regulatory Agency's (MHRA) real-world data research service. The views expressed by P.M. are her own and do not represent the MHRA's policy position.

SUPPLEMENTAL INFORMATION

Supplemental information can be found online at https://doi.org/10.1016/j.patter.2025.101320.

Received: December 7, 2024
Revised: April 29, 2025
Accepted: June 17, 2025
Published: July 29, 2025

REFERENCES

1. Doshi, P. (2016). Data too important to share: do those who control the data control the message? BMJ *352*, i1027. https://doi.org/10.1136/bmj.i1027.
2. Rankin, D., Black, M., Bond, R., Wallace, J., Mulvenna, M., and Epelde, G. (2020). Reliability of Supervised Machine Learning Using Synthetic Data in Health Care: Model to Preserve Privacy for Data Sharing. JMIR Med. Inform. *8*, e18910. https://doi.org/10.2196/18910.
3. Ventresca, M., Schünemann, H.J., Macbeth, F., Clarke, M., Thabane, L., Griffiths, G., Noble, S., Garcia, D., Marcucci, M., Iorio, A., et al. (2020). Obtaining and managing data sets for individual participant data meta-analysis: scoping review and practical guide. BMC Med. Res. Methodol. *20*, 113. https://doi.org/10.1186/s12874-020-00964-6.
4. Polanin, J.R. (2018). Efforts to retrieve individual participant data sets for use in a meta-analysis result in moderate data sharing but many data sets remain missing. J. Clin. Epidemiol. *98*, 157–159. https://doi.org/10.1016/j.jclinepi.2017.12.014.
5. Naudet, F., Sakarovitch, C., Janiaud, P., Cristea, I., Fanelli, D., Moher, D., and Ioannidis, J.P.A. (2018). Data sharing and reanalysis of randomized controlled trials in leading biomedical journals with a full data sharing policy: survey of studies published in The BMJ and PLOS Medicine. BMJ *360*, k400. https://doi.org/10.1136/bmj.k400.
6. Nevitt, S.J., Marson, A.G., Davie, B., Reynolds, S., Williams, L., and Smith, C.T. (2017). Exploring changes over time and characteristics

associated with data retrieval across individual participant data meta-analyses: systematic review. BMJ *357*, 1390. https://doi.org/10.1136/bmj.j1390.

7. Villain, B., Dechartres, A., Boyer, P., and Ravaud, P. (2015). Feasibility of individual patient data meta-analyses in orthopaedic surgery. BMC Med. *13*, 131. https://doi.org/10.1186/s12916-015-0376-6.
8. Iqbal, S.A., Wallach, J.D., Khoury, M.J., Schully, S.D., and Ioannidis, J.P.A. (2016). Reproducible Research Practices and Transparency across the Biomedical Literature. PLoS Biol. *14*, e1002333. https://doi.org/10.1371/journal.pbio.1002333.
9. Foraker, R.E., Yu, S.C., Gupta, A., Michelson, A.P., Pineda Soto, J.A., Colvin, R., Loh, F., Kollef, M.H., Maddox, T., Evanoff, B., et al. (2020). Spot the difference: comparing results of analyses from real patient data and synthetic derivatives. JAMIA Open *3*, 557–566. https://doi.org/10.1093/jamiaopen/ooaa060.
10. Tucker, A., Wang, Z., Rotalinti, Y., and Myles, P. (2020). Generating high-fidelity synthetic patient data for assessing machine learning healthcare software. npj Digit. Med. *3*, 147. https://doi.org/10.1038/s41746-020-00353-9.
11. Wang, Z., Myles, P., and Tucker, A. (2019). Generating and Evaluating Synthetic UK Primary Care Data: Preserving Data Utility Patient Privacy. In 2019 IEEE 32nd International Symposium on Computer-Based Medical Systems (CBMS), pp. 126–131. https://doi.org/10.1109/CBMS.2019.00036.
12. Wang, Z., Myles, P., and Tucker, A. (2021). Generating and evaluating cross-sectional synthetic electronic healthcare data: Preserving data utility and patient privacy. Comput. Intell. *37*, 819–851. https://doi.org/10.1111/coin.12427.
13. Reiner Benaim, A., Almog, R., Gorelik, Y., Hochberg, I., Nassar, L., Mashiach, T., Khamaisi, M., Lurie, Y., Azzam, Z.S., Khoury, J., et al. (2020). Analyzing Medical Research Results Based on Synthetic Data and Their Relation to Real Data Results: Systematic Comparison From Five Observational Studies. JMIR Med. Inform. *8*, e16492. https://doi.org/10.2196/16492.
14. Mendelevitch, O., and Lesh, M.D. (2021). Fidelity and Privacy of Synthetic Medical Data. Preprint at arXiv. https://arxiv.org/abs/2101.08658.
15. Muniz-Terrera, G., Mendelevitch, O., Barnes, R., and Lesh, M.D. (2021). Virtual Cohorts and Synthetic Data in Dementia: An Illustration of Their Potential to Advance Research. Front. Artif. Intell. *4*, 613956. https://doi.org/10.3389/frai.2021.613956.
16. Foraker, R., Guo, A., Thomas, J., Zamstein, N., Payne, P.R., and Wilcox, A.; N3C Collaborative (2021). Analyses of Original and Computationally-Derived Electronic Health Record Data: The National COVID Cohort Collaborative. J. Med. Internet Res. *23*, e30697. https://doi.org/10.2196/30697.
17. Azizi, Z., Zheng, C., Mosquera, L., Pilote, L., and El Emam, K.; GOING-FWD Collaborators (2021). Can synthetic data be a proxy for real clinical trial data? A validation study. BMJ Open *11*, e043497. https://doi.org/10.1136/bmjopen-2020-043497.
18. El Emam, K., Mosquera, L., Jonker, E., and Sood, H. (2021). Evaluating the utility of synthetic COVID-19 case data. JAMIA Open *4*, ooab012. https://doi.org/10.1093/jamiaopen/ooab012.
19. Beaulieu-Jones, B.K., Wu, Z.S., Williams, C., Lee, R., Bhavnani, S.P., Byrd, J.B., and Greene, C.S. (2019). Privacy-Preserving Generative Deep Neural Networks Support Clinical Data Sharing. Circ. Cardiovasc. Qual. Outcomes *12*, e005122. https://doi.org/10.1161/CIRCOUTCOMES.118.005122.
20. El Emam, K., Mosquera, L., and Hoptroff, R. (2020). Practical Synthetic Data Generation: Balancing Privacy and the Broad Availability of Data (O'Reilly Media).
21. Haendel, M.A., Chute, C.G., Bennett, T.D., Eichmann, D.A., Guinney, J., Kibbe, W.A., Payne, P.R.O., Pfaff, E.R., Robinson, P.N., Saltz, J.H., et al. (2021). The National COVID Cohort Collaborative (N3C): Rationale, design, infrastructure, and deployment. J. Am. Med. Inform. Assoc. *28*, 427–443. https://doi.org/10.1093/jamia/ocaa196.
22. Hod, S., and Canetti, R. (2024). Differentially Private Release of Israel's National Registry of Live Births. Preprint at arXiv. https://arxiv.org/abs/2405.00267v1.
23. Lun, R., Siegal, D., Ramsay, T., Stotts, G., and Dowlatshahi, D. (2024). Synthetic data in cancer and cerebrovascular disease research: A novel approach to big data. PLoS One *19*, e0295921. https://doi.org/10.1371/journal.pone.0295921.
24. Rousseau, O., Karakachoff, M., Gaignard, A., Bellanger, L., Bijlenga, P., Constant Dit Beaufils, P., L'Allinec, V., Levrier, O., Aguettaz, P., Desilles, J.-P., et al. (2021). Location of intracranial aneurysms is the main factor associated with rupture in the ICAN population. J. Neurol. Neurosurg. Psychiatry *92*, 122–128. https://doi.org/10.1136/jnnp-2020-324371.
25. Guillaudeux, M., Rousseau, O., Petot, J., Bennis, Z., Dein, C.-A., Goronflot, T., Vince, N., Limou, S., Karakachoff, M., Wargny, M., and Gourraud, P.A. (2023). Patient-centric synthetic data generation, no reason to risk re-identification in biomedical data analysis. npj Digit. Med. *6*, 37. https://doi.org/10.1038/s41746-023-00771-5.
26. Thomas, A., Jaffré, S., Guardiolle, V., Perennec, T., Gagnadoux, F., Goupil, F., Bretonnière, C., Danielo, V., Morin, J., and Blanc, F.-X. (2024). Does PaCO2 correction have an impact on survival of patients with chronic respiratory failure and long-term non-invasive ventilation? Heliyon *10*, e26437. https://doi.org/10.1016/j.heliyon.2024.e26437.
27. Hundepool, A., Domingo-Ferrer, J., Franconi, L., Giessing, S., Schulte Nordholt, E., Spicer, K., and de Wolf, P.P. (2012). Statistical Disclosure Control (Wiley).
28. Duncan, G., Elliot, M., and Salazar, G. (2011). Statistical Confidentiality - Principles and Practice (Springer).
29. Willenborg, L., and de Waal, T. (1996). Statistical Disclosure Control in Practice (Springer-Verlag).
30. Willenborg, L., and de Wall, T. (2001). Elements of Statistical Disclosure Control, 1st ed. (New York: Springer-Verlag).
31. van Breugel, B., Liu, T., Oglic, D., and van der Schaar, M. (2024). Synthetic data in biomedicine via generative artificial intelligence. Nat. Rev. Bioeng. *2*, 991–1004. https://doi.org/10.1038/s44222-024-00245-7.
32. Kaabachi, B., Despraz, J., Meurers, T., Otte, K., Halilovic, M., Kulynych, B., Prasser, F., and Raisaro, J.L. (2025). A scoping review of privacy and utility metrics in medical synthetic data. npj Digit. Med. *8*, 60. https://doi.org/10.1038/s41746-024-01359-3.
33. Drechsler, J., and Haensch, A.-C. (2024). 30 Years of Synthetic Data. Stat. Sci. *39*, 221–242. https://doi.org/10.1214/24-STS927.
34. Reiter, J.P. (2023). Synthetic Data: A Look Back and A Look Forward. Trans. Data Priv. *16*, 15–24.
35. Arora, A., Wagner, S.K., Carpenter, R., Jena, R., and Keane, P.A. (2025). The urgent need to accelerate synthetic data privacy frameworks for medical research. Lancet Digit. Health *7*, e157–e160. https://doi.org/10.1016/S2589-7500(24)00196-1.
36. Boraschi, D., van der Schaar, M., Costa, A., and Milne, R. (2025). Governing synthetic data in medical research: the time is now. Lancet Digit. Health *7*, e233–e234. https://doi.org/10.1016/j.landig.2025.01.012.
37. Abgrall, G., Monnet, X., and Arora, A. (2025). Synthetic Data and Health Privacy. JAMA *333*, 567–568. https://doi.org/10.1001/jama.2024.25821.
38. International Standards Organization (2022). ISO/IEC 27559:2022: Information Security, Cybersecurity and Privacy Protection – Privacy Enhancing Data De-identification Framework (ISO).
39. U.S. Department of Health and Human Services. 45 C.F.R. § 164.514 – Other Requirements Relating to Uses and Disclosures of Protected Health Information. Electronic Code of Federal Regulations.
40. Matwin, S., Nin, J., Sehatkar, M., and Szapiro, T. (2015). A Review of Attribute Disclosure Control. In Advanced Research in Data Privacy Studies in Computational Intelligence, G. Navarro-Arribas and V. Torra, eds. (Springer International Publishing), pp. 41–61. https://doi.org/10.1007/978-3-319-09885-2_4.
41. Duncan, G.T., Keller-McNulty, S.A., and Stokes, S.L. (2001). National Institute of Statistical Sciences Task Force Report. Disclosure Risk vs.

data Utility: The R-U Confidentiality Map. https://www.niss.org/sites/default/files/research_attachments/Disclosure%20Risk%20vs%20Data%20Utility-FT.pdf.

42. Office of the Information and Privacy Commissioner of Ontario (2016). Deidentification Guidelines for Structured Data (Office of the Information and Privacy Commissioner of Ontario).

43. El Emam, K., Mosquera, L., and Fang, X. (2022). Validating A Membership Disclosure Metric For Synthetic Health Data. JAMIA Open *5*, ooac083. https://doi.org/10.1093/jamiaopen/ooac083.

44. Francis, P., and Wagner, D. (2024). Towards more accurate and useful data anonymity vulnerability measures. Preprint at arXiv. https://arxiv.org/abs/2403.06595.

45. Elliot, M., and Dale, A. (1999). Scenarios of Attack: The Data Intruders Perspective on Statistical Disclosure Risk. Netherlands Official Statistics *14*, 6–10.

46. Elliot, M., Mandalari, A.M., Mourby, M., O'Hara, K., and Fabian, H. (2024). Dictionary of Privacy, Data Protection and Information Security. (Edward Elgar Publishing Limited). https://doi.org/10.4337/9781035300921.

47. Pilgram, L., Dankar, F.K., Drechsler, J., Elliot, M., Domingo-Ferrer, J., Francis, P., Kantarcioglu, M., Malin, B., Muralidhar, K., Myles, P., et al. (2025). A Consensus Privacy Metrics Framework for Synthetic Data - Critical Analysis of Privacy Metrics (Report) (OSF). https://osf.io/vz5x9.

48. Vallevik, V.B., Babic, A., Marshall, S.E., Elvatun, S., Brøgger, H.M.B., Alagaratnam, S., Edwin, B., Veeraragavan, N.R., Befring, A.K., and Nygård, J.F. (2024). Can I trust my fake data - A comprehensive quality assessment framework for synthetic tabular data in healthcare. Int. J. Med. Inform. *185*, 105413. https://doi.org/10.1016/j.ijmedinf.2024.105413.

49. Boudewijn, A., Ferraris, A.F., Panfilo, D., Cocca, V., Zinutti, S., De Schepper, K., and Chauvenet, C.R. (2023). Privacy Measurement in Tabular Synthetic Data: State of the Art and Future Research Directions. Preprint at arXiv. https://doi.org/10.48550/arXiv.2311.17453.

50. Budu, E., Etminani, K., Soliman, A., and Rögnvaldsson, T. (2024). Evaluation of synthetic electronic health records: A systematic review and experimental assessment. Neurocomputing *603*, 128253. https://doi.org/10.1016/j.neucom.2024.128253.

51. Francis, P. (2022). A Note on the Misinterpretation of the US Census Re-identification Attack. In International Conference on Privacy in Statistical Databases, pp. 299–311. https://doi.org/10.1007/978-3-031-13945-1_21.

52. Muralidhar, K., and Domingo-Ferrer, J. (2023). Database Reconstruction Is Not So Easy and Is Different from Reidentification. J. Off. Stat. *39*, 381–398. https://doi.org/10.2478/jos-2023-0017.

53. Von der Gracht, H.A. (2012). Consensus measurement in Delphi studies: Review and implications for future quality assurance. Technol. Forecast. Soc. Change *79*, 1525–1536. https://doi.org/10.1016/j.techfore.2012.04.013.

54. De Vet, E., Brug, J., De Nooijer, J., Dijkstra, A., and De Vries, N.K. (2005). Determinants of forward stage transitions: a Delphi study. Health Educ. Res. *20*, 195–205. https://doi.org/10.1093/her/cyg111.

55. El Emam, K. (2013). Guide to the De-identification of Personal Health Information (CRC Press (Auerbach)).

56. Giomi, M., Boenisch, F., Wehmeyer, C., and Tasnádi, B. (2023). A Unified Framework for Quantifying Privacy Risk in Synthetic Data. Preprint at arXiv. https://doi.org/10.48550/arXiv.2211.10459.

57. Meurers, T., Baum, L., Haber, A.C., Halilovic, M., Heinz, B., Milicevic, V., Neves, D.T., Otte, K., Pasquier, A., Poikela, M., et al. (2024). Health Data Re-Identification: Assessing Adversaries and Potential Harms. Stud. Health Technol. Inform. *316*, 1199–1203. https://doi.org/10.3233/SHTI240626.

58. Wan, Z., Vorobeychik, Y., Xia, W., Liu, Y., Wooders, M., Guo, J., Yin, Z., Clayton, E.W., Kantarcioglu, M., and Malin, B.A. (2021). Using game theory to thwart multistage privacy intrusions when sharing data. Sci. Adv. *7*, eabe9986. https://doi.org/10.1126/sciadv.abe9986.

59. Wan, Z., Vorobeychik, Y., Xia, W., Clayton, E.W., Kantarcioglu, M., Ganta, R., Heatherly, R., and Malin, B.A. (2015). A game theoretic framework for analyzing re-identification risk. PLoS One *10*, e0120592. https://doi.org/10.1371/journal.pone.0120592.

60. Stadler, T., Oprisanu, B., and Troncoso, C. (2022). Synthetic Data – Anonymisation Groundhog Day. Preprint at arXiv. https://arxiv.org/abs/2011.07018.

61. Taub, J., Elliot, M., Pampaka, M., and Smith, D. (2018). Differential Correct Attribution Probability for Synthetic Data: An Exploration. In Privacy in Statistical Databases Lecture Notes in Computer Science, J. Domingo-Ferrer and F. Montes, eds. (Springer International Publishing), pp. 122–137.

62. Oprisanu, B., Ganev, G., and De Cristofaro, E. (2022). On Utility and Privacy in Synthetic Genomic Data. Preprint at arXiv. https://doi.org/10.48550/arXiv.2102.03314.

63. Stadler, T., Oprisanu, B., and Troncoso, C. (2020). Synthetic Data – A Privacy Mirage. Preprint at arXiv. https://arxiv.org/abs/2011.07018v2.

64. Kotelnikov, A., Baranchuk, D., Rubachev, I., and Babenko, A. (2022). TabDDPM: Modelling Tabular Data with Diffusion Models. Preprint at arXiv. https://arxiv.org/abs/2209.15421.

65. Lu, P.-H., Wang, P.-C., and Yu, C.-M. (2019). Empirical Evaluation on Synthetic Data Generation with Generative Adversarial Network. In Proceedings of the 9th International Conference on Web Intelligence, Mining and Semantics WIMS2019 (Association for Computing Machinery), pp. 1–6. https://doi.org/10.1145/3326467.3326474.

66. Platzer, M., and Reutterer, T. (2021). Holdout-Based Empirical Assessment of Mixed-Type Synthetic Data. Front. Big Data *4*, 679939. https://doi.org/10.3389/fdata.2021.679939.

67. Yale, A., Dash, S., Dutta, R., Guyon, I., Pavao, A., and Bennett, K.P. (2020). Generation and evaluation of privacy preserving synthetic health data. Neurocomputing *416*, 244–255. https://doi.org/10.1016/j.neucom.2019.12.136.

68. D'Acquisto, G., Cohen, A., Naldi, M., and Nissim, K. (2024). From Isolation to Identification. In Privacy in Statistical Databases, J. Domingo-Ferrer and M. Önen, eds. (Springer Nature Switzerland), pp. 3–17. https://doi.org/10.1007/978-3-031-69651-0_1.

69. Raab, G.M., Nowok, B., and Dibben, C. (2024). Practical privacy metrics for synthetic data. Preprint at arXiv. https://doi.org/10.48550/arXiv.2406.16826.

70. Rocher, L., Hendrickx, J.M., and de Montjoye, Y.-A. (2019). Estimating the success of re-identifications in incomplete datasets using generative models. Nat. Commun. *10*, 1–9. https://doi.org/10.1038/s41467-019-10933-3.

71. Yan, C., Yan, Y., Wan, Z., Zhang, Z., Omberg, L., Guinney, J., Mooney, S. D., and Malin, B.A. (2022). A Multifaceted benchmarking of synthetic electronic health record generation models. Nat. Commun. *13*, 7609. https://doi.org/10.1038/s41467-022-35295-1.

72. Choi, E., Biswal, S., Malin, B., Duke, J., Stewart, W.F., and Sun, J. (2017). Generating Multi-label Discrete Patient Records using Generative Adversarial Networks. In Proceedings of Machine Learning for Healthcare 2017 (MLResearchPress), pp. 286–305.

73. El Kababji, S., Mitsakakis, N., Fang, X., Beltran-Bless, A.-A., Pond, G., Vandermeer, L., Radhakrishnan, D., Mosquera, L., Paterson, A., Shepherd, L., et al. (2023). Evaluating the Utility and Privacy of Synthetic Breast Cancer Clinical Trial Datasets. J. Clin. Orthod. *7*, e2300116. https://doi.org/10.1200/CCI.23.00116.

74. Goncalves, A., Ray, P., Soper, B., Stevens, J., Coyle, L., and Sales, A.P. (2020). Generation and evaluation of synthetic patient data. BMC Med. Res. Methodol. *20*, 108. https://doi.org/10.1186/s12874-020-00977-1.

75. Zhang, Z., Yan, C., and Malin, B.A. (2022). Membership inference attacks against synthetic health data. J. Biomed. Inform. *125*, 103977. https://doi.org/10.1016/j.jbi.2021.103977.

76. Zhang, Z., Yan, C., Mesa, D.A., Sun, J., and Malin, B.A. (2020). Ensuring electronic medical record simulation through better training, modeling, and evaluation. J. Am. Med. Inform. Assoc. *27*, 99–108. https://doi.org/10.1093/jamia/ocz161.

77. Torfi, A., and Fox, E.A. (2020). CorGAN: Correlation-Capturing Convolutional Generative Adversarial Networks for Generating Synthetic Healthcare Records. Preprint at arXiv. https://arxiv.org/abs/2001.09346.

78. Ghosheh, G.O., Li, J., and Zhu, T. (2024). A Survey of Generative Adversarial Networks for Synthesizing Structured Electronic Health Records. ACM Comput. Surv. *56*, 1–34. https://doi.org/10.1145/3636424.

79. Park, N., Mohammadi, M., Gorde, K., Jajodia, S., Park, H., and Kim, Y. (2018). Data synthesis based on generative adversarial networks. Proc. VLDB Endow. *11*, 1071–1083. https://doi.org/10.14778/3231751.3231757.

80. Kuppa, A., Aouad, L., and Le-Khac, N.-A. (2021). Towards Improving Privacy of Synthetic DataSets. In Privacy Technologies and Policy, N. Gruschka, L.F.C. Antunes, K. Rannenberg, and P. Drogkaris, eds. (Springer International Publishing), pp. 106–119. https://doi.org/10.1007/978-3-030-76663-4_6.

81. Hayes, J., Melis, L., Danezis, G., and De Cristofaro, E. (2018). LOGAN: Membership Inference Attacks Against Generative Models. Preprint at arXiv. https://doi.org/10.48550/arXiv.1705.07663.

82. Hu, H., and Pang, J. (2021). Membership Inference Attacks against GANs by Leveraging Over-representation Regions. In Proceedings of the 2021 ACM SIGSAC Conference on Computer and Communications Security CCS '21 (Association for Computing Machinery), pp. 2387–2389. https://doi.org/10.1145/3460120.3485338.

83. Houssiau, F., Jordon, J., Cohen, S.N., Daniel, O., Elliott, A., Geddes, J., Mole, C., Rangel-Smith, C., and Szpruch, L. (2022). TAPAS: a Toolbox for Adversarial Privacy Auditing of Synthetic Data. Preprint at arXiv. https://doi.org/10.48550/arXiv.2211.06550.

84. Meeus, M., Guépin, F., Cretu, A.-M., and de Montjoye, Y.-A. (2024). Achilles' Heels: Vulnerable Record Identification in Synthetic Data Publishing. In Computer Security – ESORIC 2023 Lecture Notes in Computer Science, G. Tsudik, M. Conti, K. Liang, and G. Smaragdakis, eds. (Cham: Springer), pp. 380–399. https://doi.org/10.1007/978-3-031-51476-0_19.

85. Van Breugel, B., Sun, H., Qian, Z., and van der Schaar, M. (2023). Membership Inference Attacks against Synthetic Data through Overfitting Detection. Preprint at arXiv. https://doi.org/10.48550/arXiv.2302.12580.

86. Li, J., Cairns, B.J., Li, J., and Zhu, T. (2023). Generating synthetic mixed-type longitudinal electronic health records for artificial intelligent applications. npj Digit. Med. *6*, 98. https://doi.org/10.1038/s41746-023-00834-7.

87. Hyeong, J., Kim, J., Park, N., and Jajodia, S. (2022). An Empirical Study on the Membership Inference Attack against Tabular Data Synthesis Models. Preprint at arXiv. https://doi.org/10.48550/arXiv.2208.08114.

88. Chen, D., Yu, N., Zhang, Y., and Fritz, M. (2020). GAN-Leaks: A Taxonomy of Membership Inference Attacks against Generative Models. In Proceedings of the 2020 ACM SIGSAC Conference on Computer and Communications Security (ACM), pp. 343–362. https://doi.org/10.1145/3372297.3417238.

89. Shokri, R., Stronati, M., Song, C., and Shmatikov, V. (2017). Membership Inference Attacks Against Machine Learning Models. In 2017 IEEE Symposium on Security and Privacy (SP), pp. 3–18. https://doi.org/10.1109/SP.2017.41.

90. Maeda, W., Higuchi, Y., Minami, K., and Morikawa, I. (2022). Membership Inference Countermeasure With A Partially Synthetic Data Approach. In 2022 4th International Conference on Data Intelligence and Security (ICDIS), pp. 374–381. https://doi.org/10.1109/ICDIS55630.2022.00063.

91. Yale, A., Dash, S., Dutta, R., Guyon, I., Pavao, A., and Bennett, K.P. (2019). Assessing privacy and quality of synthetic health data. In Proceedings of the Conference on Artificial Intelligence for Data Discovery and Reuse AIDR '19 (Association for Computing Machinery), pp. 1–4. https://doi.org/10.1145/3359115.3359124.

92. Hand, D.J., Christen, P., and Ziyad, S. (2024). Selecting a classification performance measure: matching the measure to the problem. Preprint at arXiv. https://arxiv.org/abs/2409.12391v1.

93. Li, J., and Fine, J.P. (2011). Assessing the dependence of sensitivity and specificity on prevalence in meta-analysis. Biostatistics *12*, 710–722. https://doi.org/10.1093/biostatistics/kxr008.

94. Van Calster, B., Collins, G.S., Vickers, A.J., Wynants, L., Kerr, K.F., Barreñada, L., Varoquaux, G., Singh, K., Moons, K.G.M., Hernandez-boussard, T., et al. (2024). Performance evaluation of predictive AI models to support medical decisions: Overview and guidance. Preprint at arXiv. https://arxiv.org/abs/2412.10288v1.

95. Hittmeir, M., Mayer, R., and Ekelhart, A. (2020). A Baseline for Attribute Disclosure Risk in Synthetic Data. In Proceedings of the Tenth ACM Conference on Data and Application Security and Privacy CODASPY '20 (Association for Computing Machinery), pp. 133–143. https://doi.org/10.1145/3374664.3375722.

96. Kwatra, S., and Torra, V. (2024). Empirical Evaluation of Synthetic Data Created by Generative Models via Attribute Inference Attack. In Privacy and Identity Management. Sharing in a Digital World, F. Bieker, S. de Conca, N. Gruschka, M. Jensen, and I. Schiering, eds. (Springer Nature Switzerland), pp. 282–291. https://doi.org/10.1007/978-3-031-57978-3_18.

97. Emam, K.E., Mosquera, L., and Bass, J. (2020). Evaluating Identity Disclosure Risk in Fully Synthetic Health Data: Model Development and Validation. JMIR *22*, e23139. https://doi.org/10.2196/23139.

98. Mühlhoff, R. (2021). Predictive privacy: towards an applied ethics of data analytics. Ethics Inf. Technol. *23*, 675–690. https://doi.org/10.1007/s10676-021-09606-x.

99. Mantelero, A. (2017). From Group Privacy to Collective Privacy: Towards a New Dimension of Privacy and Data Protection in the Big Data Era. In Group Privacy: New Challenges of Data Technologies, L. Taylor, L. Floridi, and B. van der Sloot, eds. (Springer International Publishing), pp. 139–158. https://doi.org/10.1007/978-3-319-46608-8_8.

100. Personal Data Protection Commission Singapore (2024). Privacy Enhancing Technology (PET): Proposed Guide on Synthetic Data Generation. Report No.: Version 1.0. https://www.pdpc.gov.sg/-/media/files/pdpc/pdf-files/other-guides/proposed-guide-on-synthetic-data-generation.pdf.

101. UK Information Commissioner's Office (ICO) (2023). Guidance on Privacy-Enhancing Technologies. https://ico.org.uk/for-organisations/uk-gdpr-guidance-and-resources/data-sharing/privacy-enhancing-technologies/.

102. Personal Information Protection Commission of South Korea (2024). Guide for Generating and Utilizing Synthetic Data. https://www.pipc.go.kr/np/cop/bbs/selectBoardArticle.do?bbsId=BS217&mCode=G010030000&nttId=10915.

103. Dwork, C., Smith, A., Steinke, T., and Ullman, J. (2017). Exposed! A Survey of Attacks on Private Data. Annu. Rev. Stat. Appl. *4*, 61–84. https://doi.org/10.1146/annurev-statistics-060116-054123.

104. Abowd, J.M., Adams, T., Ashmead, R., Darais, D., Dey, S., Garfinkel, S. L., Goldschlag, N., Kifer, D., Leclerc, P., Lew, E., et al. (2023). The 2010 Census Confidentiality Protections Failed, Here's How and Why. Preprint at arXiv. https://doi.org/10.48550/arXiv.2312.11283.

105. Boudewijn, A., and Ferraris, A.F. (2024). Legal and Regulatory Perspectives on Synthetic Data as an Anonymization Strategy. J. Pers. Data Prot. L. *17*, 17–31. https://journal.pdps.ge/doc/18-32.pdf.

106. Hyrup, T., Lautrup, A.D., Zimek, A., and Schneider-Kamp, P. (2025). A systematic review of privacy-preserving techniques for synthetic tabular health data. Discov. Data *3*, 5. https://doi.org/10.1007/s44248-025-00022-w.

107. Kohli, N. (2024). Differentially Private Synthetic Microdata. A Practical Guide (UNHCR). https://microdata.unhcr.org/index.php/synthetic-data.

108. Rosenblatt, L., Liu, X., Pouyanfar, S., de Leon, E., Desai, A., and Allen, J. (2020). Differentially Private Synthetic Data: Applied Evaluations and Enhancements. Preprint at arXiv. https://doi.org/10.48550/arXiv.2011.05537.

109. Dwork, C., and Roth, A. (2013). The Algorithmic Foundations of Differential Privacy. Theor. Comput. Sci. *9*, 211–407. https://doi.org/10.1561/0400000042.

110. Dwork, C., Kohli, N., and Mulligan, D. (2019). Differential Privacy in Practice: Expose your Epsilons! J. Priv. Confid. *9*, https://doi.org/10.29012/jpc.689.

111. Muralidhar, K., Domingo-Ferrer, J., and Martínez, S. (2020). E-Differential Privacy for Microdata Releases Does Not Guarantee Confidentiality (Let Alone Utility). In Privacy in Statistical Databases, J. Domingo-Ferrer and K. Muralidhar, eds. (Springer Nature Switzerland), pp. 21–31. https://doi.org/10.1007/978-3-030-57521-2_2.

112. Rogers, R., Cardoso, A.R., Mancuhan, K., Kaura, A., Gahlawat, N., Jain, N., Ko, P., and Ahammad, P. (2020). A Members First Approach to Enabling LinkedIn's Labor Market Insights at Scale. Preprint at arXiv. https://arxiv.org/abs/2010.13981.

113. Abowd, J., Ashmead, R., Cumings-Menon, R., Garfinkel, S., Heineck, M., Heiss, C., Johns, R., Kifer, D., Leclerc, P., Machanavajjhala, A., et al. (2022). The 2020 Census Disclosure Avoidance System TopDown Algorithm. Harv. Data Sci. Rev. *Special Issue 2: Differential Privacy for the 2020 U.S. Census*. https://doi.org/10.1162/99608f92.529e3cb9.

114. Near, J., Darais, D., Lefkovitz, N., and Howarth, G. (2025). Guidelines for Evaluating Differential Privacy Guarantees (National Institute of Standards and Technology). https://doi.org/10.6028/NIST.SP.800-226.

115. Adams, T., Birkenbihl, C., Otte, K., Ng, H.G., Rieling, J.A., Näher, A.-F., Sax, U., Prasser, F., and Fröhlich, H.; Alzheimer's Disease Neuroimaging Initiative (2025). On the fidelity versus privacy and utility trade-off of synthetic patient data. iScience *28*, 112382. https://doi.org/10.1016/j.isci.2025.112382.

116. Raghunathan, T., Reiter, J., and Rubin, D. (2003). Multiple Imputation for Statistical Disclosure control. J. Off. Stat. *19*, 1–16.

117. El Emam, K., Mosquera, L., Fang, X., and El-Hussuna, A. (2024). An evaluation of the replicability of analyses using synthetic health data. Sci. Rep. *14*, 6978. https://doi.org/10.1038/s41598-024-57207-7.

118. EMA (2018). External Guidance on the Implementation of the European Medicines Agency Policy on the Publication of Clinical Data for Medicinal Products for Human Use. https://www.ema.europa.eu/en/human-regulatory-overview/marketing-authorisation/clinical-data-publication/support-industry-clinical-data-publication/external-guidance-implementation-european-medicines-agency-policy-publication-clinical-data-medicinal-products-human-use.

119. Health Canada (2019). Guidance document on Public Release of Clinical Information. https://www.canada.ca/en/health-canada/services/drug-health-product-review-approval/profile-public-release-clinical-information-guidance.html.

120. Steyerberg, E.W. (2019). Overfitting and Optimism in Prediction Models. In Clinical Prediction Models: A Practical Approach to Development, Validation, and Updating, E.W. Steyerberg, ed. (Springer International Publishing), pp. 95–112. https://doi.org/10.1007/978-3-030-16399-0_5.

121. Copas, J.B. (1983). Regression, Prediction and Shrinkage. J. Roy. Stat. Soc. B *45*, 311–335. https://doi.org/10.1111/j.2517-6161.1983.tb01258.x.

122. Efron, B. (1983). Estimating the Error Rate of a Prediction Rule: Improvement on Cross-Validation. J. Am. Stat. Assoc. *78*, 316–331. https://doi.org/10.1080/01621459.1983.10477973.

123. Malterud, K. (2001). Qualitative research: standards, challenges, and guidelines. Lancet *358*, 483–488. https://doi.org/10.1016/S0140-6736(01)05627-6.

124. Khodyakov, D., Grant, S., Kroger, J., and Bauman, M. (2023). RAND Methodological Guidance for Conducting and Critically Appraising Delphi Panels (RAND Corporation). https://www.rand.org/pubs/tools/TLA3082-1.html.

125. Fitch, K., Bernstein, S.J., Aguilar, M.D., Burnand, B., LaCalle, J.R., Lazaro, P., van het Loo, M., McDonnell, J., Vader, J., and Kahan, J.P. (2001). The RAND/UCLA Appropriateness Method User's Manual (RAND Corporation). https://www.rand.org/pubs/monograph_reports/MR1269.html.

126. Nasa, P., Jain, R., and Juneja, D. (2021). Delphi methodology in healthcare research: How to decide its appropriateness. World J. Methodol. *11*, 116–129. https://doi.org/10.5662/wjm.v11.i4.116.

127. Fink, A., Kosecoff, J., Chassin, M., and Brook, R.H. (1984). Consensus methods: characteristics and guidelines for use. Am. J. Public Health *74*, 979–983.

128. Jones, J., and Hunter, D. (1995). Qualitative Research: Consensus methods for medical and health services research. BMJ *311*, 376–380. https://doi.org/10.1136/bmj.311.7001.376.

129. Moher, D., Schulz, K.F., Simera, I., and Altman, D.G. (2010). Guidance for Developers of Health Research Reporting Guidelines. PLoS Med. *7*, e1000217. https://doi.org/10.1371/journal.pmed.1000217.

130. Schlussel, M.M., Sharp, M.K., de Beyer, J.A., Kirtley, S., Logullo, P., Dhiman, P., MacCarthy, A., Koroleva, A., Speich, B., Bullock, G.S., et al. (2023). Reporting guidelines used varying methodology to develop recommendations. J. Clin. Epidemiol. *159*, 246–256. https://doi.org/10.1016/j.jclinepi.2023.03.018.

131. Gattrell, W.T., Logullo, P., van Zuuren, E.J., Price, A., Hughes, E.L., Blazey, P., Winchester, C.C., Tovey, D., Goldman, K., Hungin, A.P., and Harrison, N. (2024). ACCORD (ACcurate COnsensus Reporting Document): A reporting guideline for consensus methods in biomedicine developed via a modified Delphi. PLoS Med. *21*, e1004326. https://doi.org/10.1371/journal.pmed.1004326.

132. Jünger, S., Payne, S.A., Brine, J., Radbruch, L., and Brearley, S.G. (2017). Guidance on Conducting and REporting DElphi Studies (CREDES) in palliative care: Recommendations based on a methodological systematic review. Palliat. Med. *31*, 684–706. https://doi.org/10.1177/0269216317690685.

133. Akins, R.B., Tolson, H., and Cole, B.R. (2005). Stability of response characteristics of a Delphi panel: application of bootstrap data expansion. BMC Med. Res. Methodol. *5*, 37. https://doi.org/10.1186/1471-2288-5-37.

134. Dajani, J.S., Sincoff, M.Z., and Talley, W.K. (1979). Stability and agreement criteria for the termination of Delphi studies. Technol. Forecast. Soc. Change *13*, 83–90. https://doi.org/10.1016/0040-1625(79)90007-6.

135. Diamond, I.R., Grant, R.C., Feldman, B.M., Pencharz, P.B., Ling, S.C., Moore, A.M., and Wales, P.W. (2014). Defining consensus: A systematic review recommends methodologic criteria for reporting of Delphi studies. J. Clin. Epidemiol. *67*, 401–409. https://doi.org/10.1016/j.jclinepi.2013.12.002.

136. Van Zuuren, E.J., Logullo, P., Price, A., Fedorowicz, Z., Hughes, E.L., and Gattrell, W.T. (2022). Existing guidance on reporting of consensus methodology: a systematic review to inform ACCORD guideline development. BMJ Open *12*, e065154. https://doi.org/10.1136/bmjopen-2022-065154.

137. Pilgram, L., and El Emam, K. (2024). Consensus Privacy Metrics Framework (OSF). https://doi.org/10.17605/OSF.IO/QAHUV.

Patterns, Volume *6*

Supplemental information

A consensus privacy metrics

framework for synthetic data

Lisa Pilgram, Fida Kamal Dankar, Jörg Drechsler, Mark Elliot, Josep Domingo-Ferrer, Paul Francis, Murat Kantarcioglu, Linglong Kong, Bradley Malin, Krishnamurty Muralidhar, Puja Myles, Fabian Prasser, Jean Louis Raisaro, Chao Yan, and Khaled El Emam

Supplemental Methods and Notes

Evolution of Statements, Explanations and Qualitative Analysis

The initial question for the consensus process was formulated as follows "How should we evaluate privacy in synthetic data". From this question, our critical analysis (i.e., the report in [1]) was developed which then served to identify relevant statements around privacy metrics in synthetic data.

In the first round, there were 15 statements to score. In the second round, statements were revised to address any ambiguity, two new statements were introduced and four statements omitted based on the panelists' feedback. In the third round, two more statements were omitted so that a total of 11 statements were considered in the final round.

In the scoring rounds, panelists scored statements while having the report (as provided in [1]) as background information. The level of agreement was indicated on a five-point Likert scale and comments could be provided to give explanations for the indicated level of agreement. The scoring rounds were conducted online using the Welphi software. In the online tool, each statement was presented to the panelist with an explanation to clarify the meaning and underlying reasoning.

In the following, we explain the evolution of statements including rephrasing, removal, or introduction of each statement (S1 to S17) and its explanation (E1 to E17). The numbering is meant as unique identifier of the statement. If, for example, S1 is rephrased without changes in meaning (i.e., minor change), a version number is added and it becomes S1.1; if the statement changes its initial meaning (i.e., major change), it is introduced as a new statement with a new identifier. The numbering aligned with the chronological order of the rounds meaning that the numbering becomes discontinuous throughout the study due to the omission of certain statements and the introduction of new ones. If statements or explanations are rephrased, their identifier is highlighted in bold in the round when the change is introduced.

We further present the qualitative analysis from panelists' comments with respect to statements where an agreement score below 4 was indicated in the final round. Note that the qualitative analysis was limited by the panelists' comments, meaning that there were statements (e.g. S16.1) where an agreement level below 4 was indicated but no comment provided. Key findings from this analysis have also been included in the main manuscript where they help to understand the outcomes of the Delphi process.

The panelists' comments were analysed with two objectives: First, to refine the statements and the report in between the rounds and second, to understand the perspective of panelists who indicated an agreement level below 4 in the statements. The comments were coded according to key topics for each statement [2,3]. Key topics were then classified into the following categories:

1. Misunderstandings and ambiguity: Topics that resulted from misunderstandings and reflected ambiguity in the report and/or statement.
2. Misconceptions: Topics that resulted from misconceptions that have been proved wrong in literature and/or through simulations in the report.
3. Counterarguments: Topics that covered remaining counterarguments to the statement.
4. Unrelated: Topics that were unrelated to the disagreement or uncertainty in the statement.

While comments of categories 1 and 2 were used in between the rounds to adjust the statements, category 3 informed the discussion presented in this manuscript.

Threat Modeling

The Adversary’s Background Knowledge (S1)

Table S1. Statement S1

	**Round 1**	**Round 2**	**Round 3 (Final Round)**
**Statement**	S1: Disclosure vulnerability metrics should be based on quasi-identifiers rather than on complete records.	S1: Disclosure vulnerability metrics should be based on quasi-identifiers rather than on complete records.	**S1.1**: Disclosure vulnerability metrics should be based on quasi-identifiers. These may vary depending on the data context (e.g., can still be all attributes) and are ascertained by the data controller.

**Explanation**	E1: Decades of research and practice on identity disclosure in anonymized data, known re-identification attacks, and respective guidelines are based on the assumption that adversary prior knowledge is represented by the quasi-identifiers. Assuming that an adversary knows all of the variables is not necessarily a worst-case assumption for synthetic data.	**E1.1**: Quasi-identifies represent the background knowledge of an adversary. The primary reasons for this recommendation are:<br><br>(a) Decades of research and practice on identity disclosure in anonymized data, known re-identification attacks, and respective guidelines are based on the assumption that adversary prior knowledge is represented by the quasi-identifiers.<br><br>(b) Assuming that an adversary knows all of the variables is not necessarily a worst-case assumption for synthetic data (please see report section 1.5.1 in the report for an explanation of this point). It can be shown that by considering all of the variables we may be underestimating disclosure vulnerability.<br><br>(c) There are generally accepted criteria for deciding what a quasi-identifier is. Quasi-identifiers can vary and are ultimately ascertained by the data controller for a given dataset.	E1.1: Quasi-identifies represent the background knowledge of an adversary. The primary reasons for this recommendation are:<br><br>(a) Decades of research and practice on identity disclosure in anonymized data, known re-identification attacks, and respective guidelines are based on the assumption that adversary prior knowledge is represented by the quasi-identifiers.<br><br>(b) Assuming that an adversary knows all of the variables is not necessarily a worst-case assumption for synthetic data (please see section 1.5.1 in the report for a detailed explanation and justification for this point). It can be shown that by considering all the variables we may be underestimating disclosure vulnerability.<br><br>(c) There are generally accepted criteria for deciding what a quasi-identifier is. Quasi-identifiers can vary and are ultimately ascertained by the data controller for a given dataset. Treating all attributes as quasi-identifiers is itself an assumption.
**Commentary**	NA	The explanation was rephrased to include further details from the report as a rationale for this statement.	The rephrasing of the statement did not change its meaning but incorporated parts of the explanation.

**Score Distribution**	Strongly Disagree: 0/13 (0.0%)<br>Disagree: 3/13 (23.1%)<br>Neutral: 3/13 (23.1%)<br>Agree: 4/13 (30.8%)<br>Strongly Agree: 3/13 (23.1%)	Strongly Disagree: 0/13 (0.0%)<br>Disagree: 3/13 (23.1%)<br>Neutral: 2/13 (15.4%)<br>Agree: 5/13 (38.5%)<br>Strongly Agree: 3/13 (23.1%)	Strongly Disagree: 0/13 (0.0%)<br>**Disagree: 2/13 (15.4%)**<br>**Neutral: 1/13 (7.7%)**<br>Agree: 7/13 (53.8%)<br>Strongly Agree: 3/13 (23.1%)
**Qualitative Analysis**	Even though there was stable consensus on agreement for this recommendation, we want to acknowledge that there were 2/13 (15.4%) experts who disagreed with this statement. While our simulation on quasi-identifiers (QIs) in the critical analysis (see [1]) provided compelling evidence against the first argument, subjectivity in defining QIs remained as a key topic of disagreement. For example, the diagnosis of obesity or diabetes mellitus can be considered as a QI since its knowability can be quite high (e.g., public awareness, photos, discussions on social media) while the diagnosis of interstitial nephropathy may not be a familiar term to the patient themselves. Consequently, it can be challenging to label the attribute *diagnosis* as either QI or non-QI across all diagnoses. Also, the context of the data may shift over time and an attribute that was once deemed not to be a QI may then be considered one. This contextual interpretation is very likely to result in inter-individual variability. Another key point was made that any attribute could be a QI depending on the adversary so that all attributes in a record should be considered as QIs. While it may be the most prudent approach to treat all attributes as QIs, this could, in practice, become computationally problematic as a prudent approach would also require considering all potential combinations of those QIs when calculating the metrics.		
**Score Distribution Plot**	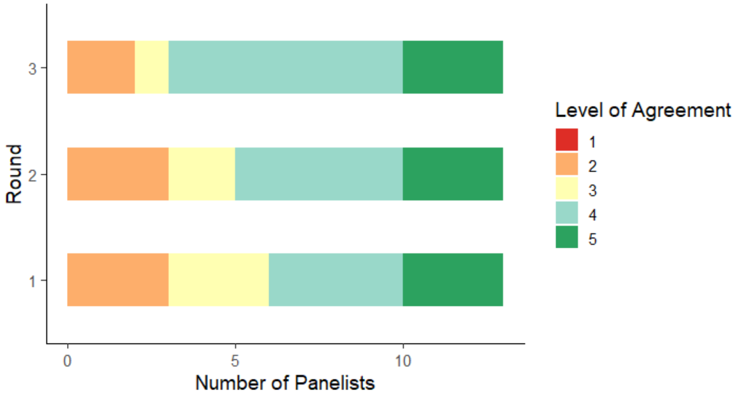		

Motivations, Constraints and Targets (S2)

Table S2. Statement S2

	**Round 1**	**Round 2**	**Round 3 (Final Round)**
**Statement**	S2: Disclosure vulnerability metrics should not be calculated on a pre-selected subset of “vulnerable” records but for all of the records.	S2: Disclosure vulnerability metrics should not be calculated on a pre-selected subset of “vulnerable” records but for all of the records.	S2: Disclosure vulnerability metrics should not be calculated on a pre-selected subset of “vulnerable” records but for all of the records.
**Explanation**	E2: Pre-selection of records mainly involves choosing “vulnerable” records but it remains unclear if these records indeed are the ones with highest disclosure vulnerability or rather vulnerable in a more ethical sense.	**E2.1**: Pre-selection of records mainly involves choosing “vulnerable” records but it remains unclear if these records indeed are the ones with highest disclosure vulnerability or rather vulnerable in a more ethical sense. Knowledge on which records are the ones with highest disclosure vulnerability can only be obtained when calculating the vulnerability for all records in the first place.	E2.1: Pre-selection of records mainly involves choosing “vulnerable” records but it remains unclear if these records indeed are the ones with highest disclosure vulnerability or rather vulnerable in a more ethical sense. Knowledge on which records are the ones with highest disclosure vulnerability can only be obtained when calculating the vulnerability for all records in the first place.
**Commentary**	NA	The explanation was rephrased to include further details from the report as a rationale for this statement.	NA
**Score Distribution**	Strongly Disagree: 0/13 (0.0%)<br>Disagree: 0/13 (0.0%)<br>Neutral: 3/13 (23.1%)<br>Agree: 3/13 (23.1%)<br>Strongly Agree: 7/13 (53.8%)	Strongly Disagree: 0/13 (0.0%)<br>Disagree: 0/13 (0.0%)<br>Neutral: 2/13 (15.4%)<br>Agree: 2/13 (15.4%)<br>Strongly Agree: 9/13 (69.2%)	Strongly Disagree: 0/13 (0.0%)<br>Disagree: 0/13 (0.0%)<br>Neutral: 2/13 (15.4%)<br>Agree: 2/13 (15.4%)<br>Strongly Agree: 9/13 (69.2%)

Score Distribution Plot	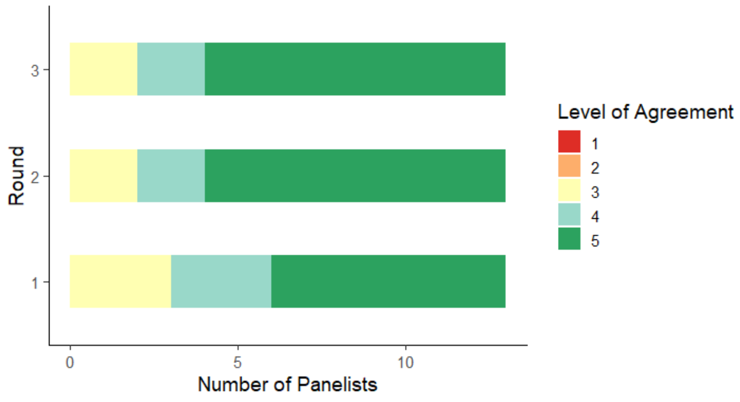

Identity Disclosure

Record-Level Similarity (S3)

Table S3. Statement S3

	**Round 1**	**Round 2**	**Round 3 (Final Round)**
**Statement**	S3: Similarity metrics that are not part of attribute or membership disclosure should not be used to report privacy in synthetic data.	**S3.1**: Stand-alone similarity metrics (i.e., that are not part of attribute or membership disclosure) should not be used to report privacy in synthetic data.	S3.1: Stand-alone similarity metrics (i.e., that are not part of attribute or membership disclosure) should not be used to report privacy in synthetic data.
**Explanation**	E3: Similarity metrics that are not part of calculating membership or attribute disclosure metrics have no precise interpretation. In contrast to anonymized data, matching a synthetic to training record by itself does not translate into an identity disclosure vulnerability as it does not imply learning correct information. Similarity by itself may or may not translate into disclosure vulnerability. This ambiguity when interpreting such metrics makes clear that they are not proper disclosure vulnerability metrics.	**E3.1**: Stand-alone similarity metrics (in fully synthetic data) that are not part of calculating membership or attribute disclosure metrics have no precise interpretation. Our analysis in Section 2.2.6 shows that these metrics do not convey additional information beyond membership and attribute disclosure metrics.	E3.1: Stand-alone similarity metrics (in fully synthetic data) that are not part of calculating membership or attribute disclosure metrics have no precise interpretation. Our analysis in Section 2.2.6 shows that these metrics do not convey additional information beyond membership and attribute disclosure metrics.
**Commentary**	NA	The rephrasing in the statement did not change its meaning but avoided ambiguity in the definition of record-level similarity (versus its use as part of other metrics). The explanation was rephrased to include further details from the report as a rationale for this statement.	NA
**Score**	Strongly Disagree: 0/13 (0.0%)	Strongly Disagree: 0/13 (0.0%)	Strongly Disagree: 0/13 (0.0%)

	Disagree: 0/13 (0.0%)<br>Neutral: 3/13 (23.1%)<br>Agree: 6/13 (46.1%)<br>Strongly Agree: 4/13 (30.8%)	Disagree: 0/13 (0.0%)<br>Neutral: 2/13 (15.4%)<br>Agree: 6/13 (46.1%)<br>Strongly Agree: 5/13 (38.5%)	Disagree: 0/13 (0.0%)<br>Neutral: 2/13 (15.4%)<br>Agree: 7/13 (53.8%)<br>Strongly Agree: 4/13 (30.8%)
**Score Distribution Plot**	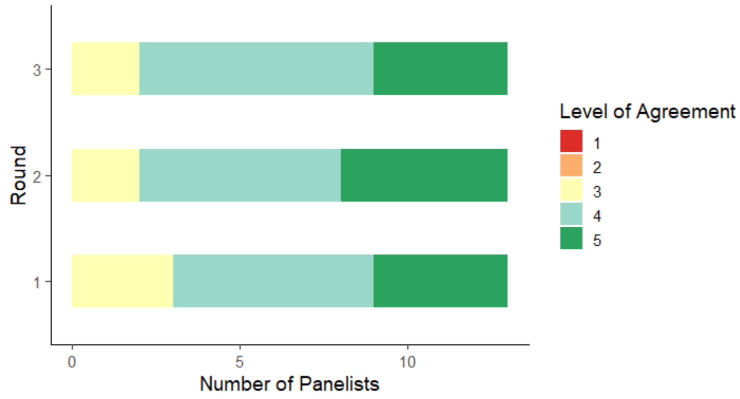		

Membership Disclosure

Assumptions in Narrative and Metric (S4)

Table S4. Statement S4

	**Round 1**	**Round 2**	**Round 3 (Final Round)**
**Statement**	S4: Membership disclosure vulnerability should only be evaluated when the adversary would learn something new for targets drawn from the same population as the training dataset.	**S4.1:** Membership disclosure vulnerability should only be evaluated when the assumptions of the current metrics hold which is that the adversary would learn something new for targets drawn from the same population as the training dataset.	S4.1: Membership disclosure vulnerability should only be evaluated when the assumptions of the current metrics hold which is that the adversary would learn something new for targets drawn from the same population as the training dataset.
**Explanation**	E4: Membership disclosure metrics assume that the adversary draws target records from the same population as the training dataset is drawn from. The information they learn from such an attack is membership and in case of interventional studies the information that a certain procedure was applied. A vaccination study is an example where the calculation of membership disclosure vulnerability by current metrics is meaningful.	**E4.1:** Current membership disclosure metrics assume that the adversary draws target records from the same population as the training dataset is drawn from. Most narratives, however, make the assumption that targets are drawn from a different population. While the latter may be a reasonable scenario, it is not reflected in current metrics. Therefore, current metrics should only be applied when the assumption of the metric is in line with the adversarial assumptions, otherwise using the current metrics in the wrong context may give wrong vulnerability estimates.	E4.1: Current membership disclosure metrics assume that the adversary draws target records from the same population as the training dataset is drawn from. Most narratives, however, make the assumption that targets are drawn from a different population. While the latter may be a reasonable scenario, it is not reflected in current metrics. Therefore, current metrics should only be applied when the assumption of the metric is in line with the adversarial assumptions, otherwise using the current metrics in the wrong context may give wrong vulnerability estimates.
**Commentary**	NA	The rephrasing in the statement did not change its meaning but incorporated parts of the explanation. The explanation was rephrased to include further details from	NA

		the report as a rationale for this statement.	
**Score Distribution**	Strongly Disagree: 1/13 (7.7%)<br>Disagree: 2/13 (15.4%)<br>Neutral: 0/13 (0.0%)<br>Agree: 8/13 (61.5%)<br>Strongly Agree: 2/13 (15.4%)	Strongly Disagree: 2/13 (15.4%<br>Disagree: 2/13 (15.4%)<br>Neutral: 0/13 (0.0%)<br>Agree: 8/13 (61.5%)<br>Strongly Agree: 1/13 (7.7%)	Strongly Disagree: 0/13 (0.0%)<br>**Disagree: 1/13 (7.7%)**<br>Neutral: 0/13 (0.0%)<br>Agree: 9/13 (69.2%)<br>Strongly Agree: 3/13 (23.1%)
**Qualitative Analysis**	While 12/13 (92.3%) panelists agreed to this statement, there was 1/13 (7.7%) disagreement. Key topics that have been raised as counterarguments are the benefits of measuring membership disclosure in terms of trust, the consideration of membership rather as a flavor of attribute disclosure than a disclosure by itself and the unclear informative value of membership disclosure in practice. The remaining disagreement cannot be clearly attributed to one of these key topics. Given that most common key topics in previous rounds were misunderstandings and ambiguity, it may be a remnant of that.		
**Score Distribution Plot**	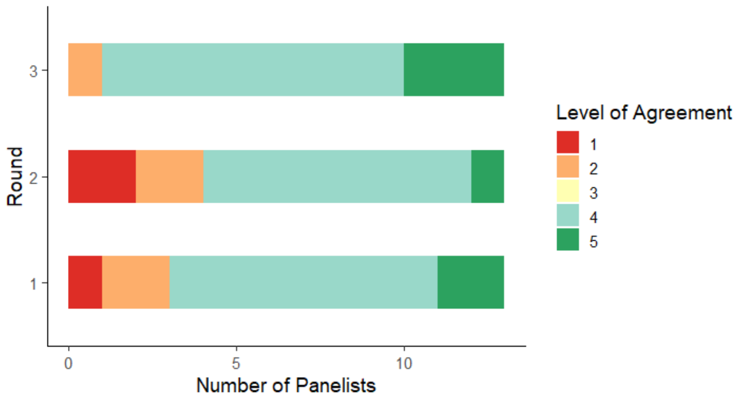		

Prevalence-Aware Interpretation: A Naïve Membership Guess (S5, S6 & S16)

Table S5. Statement S5

	Round 1	Round 2	Round 3 (Final Round)
**Statement**	S5: Calculating membership disclosure vulnerability is only meaningful when the naïve (i.e., inherent) membership disclosure vulnerability is low.	**S5**: omitted	NA
**Explanation**	E5: If the F_naive value is already high then the calculation of an absolute or relative membership disclosure vulnerability of the synthetic data does not provide meaningful guidance in decisions on SDG. The computation does not alter the conclusion that the situation exhibits high membership disclosure and the focus should only be on attribute disclosure.	NA	NA
**Commentary**	NA	The statement was removed since it was overly prescriptive and not universally applicable as good practice.	NA
**Score Distribution**	Strongly Disagree: 0/13 (0.0%)<br>Disagree: 4/13 (30.8%)<br>Neutral: 1/13 (7.7%)<br>Agree: 6/13 (46.1%)<br>Strongly Agree: 2/13 (15.4%)	NA	NA

**Score Distribution Plot**	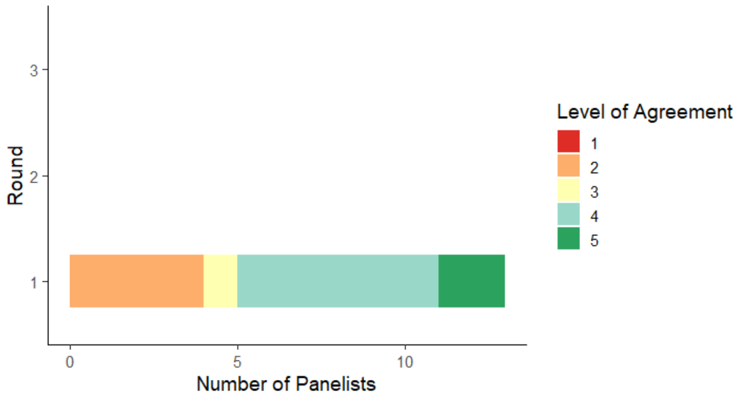

Table S6. Statement S6

	Round 1	Round 2	Round 3 (Final Round)
**Statement**	S6: In membership disclosure vulnerability, a relative vulnerability higher than its threshold should only be considered as unacceptably high when the absolute vulnerability is higher than its threshold.	**S6**: omitted	NA
**Explanation**	E6: The relative metric cannot be interpreted uniformly across the entire scale and is probably not always informative by itself.	NA	NA
**Commentary**	NA	The statement was removed since it does not hold for *F1 score* where S16 accounts for a prevalence-aware interpretation.	NA
**Score Distribution**	Strongly Disagree: 1/13 (7.7%)<br>Disagree: 0/13 (0.0%)<br>Neutral: 2/13 (15.4%)<br>Agree: 8/13 (61.5%)<br>Strongly Agree: 2/13 (15.4%)	NA	NA

Score Distribution Plot

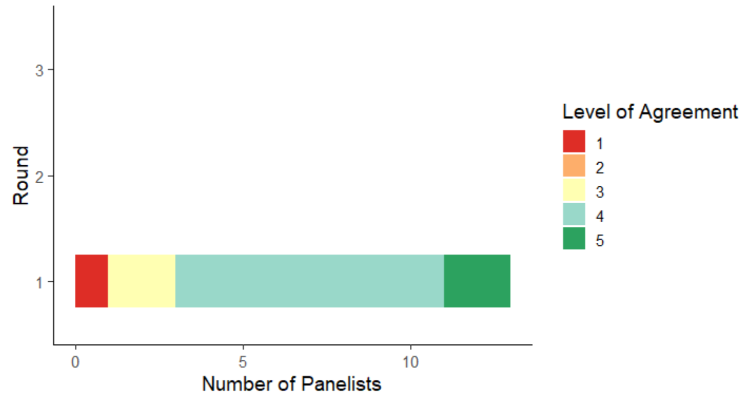

Table S7. Statement S16

	Round 1	Round 2	Round 3 (Final Round)
**Statement**	NA	**S16**: Because the most commonly used membership disclosure metric is an F1 score, and this is prevalence dependent, it needs to be reported relative to an adversary guessing membership.	**S16.1**: Because the F1 score, which is commonly used in membership disclosure metrics, is prevalence dependent, it needs to be reported relative to an adversary guessing membership.
**Explanation**	NA	**E16**: It is known that metrics like the F1 score vary with prevalence. The absolute value can therefore not be uniformly interpreted. For example, with a low naïve guess (e.g., 0.01), an F1 score of 0.5 would still be considered as high, reflecting reasonable success given the rarity of members. This naïve baseline can be accounted for by a relative metric (F_rel) or by adjusting threshold according to the baseline.	**E16.1**: It is known that metrics like the F1 score are affected by prevalence. The absolute value can therefore not be uniformly interpreted. For example, with a low naïve guess (e.g., 0.01), an F1 score of 0.5 would still be considered as high, reflecting reasonable success given the rarity of members. This naïve baseline can be accounted for by a relative metric (F_rel) or by adjusting threshold according to the baseline.
**Commentary**	NA	The statement was introduced to replace S5 & S6, addressing the same theme with a more applicable recommendation.	The rephrasing in the statement did not change the recommendation but clarified the causal explanation. The explanation was rephrased to include further details from the report as a rationale for this statement.
**Score Distribution**	NA	Strongly Disagree: 1/13 (7.7%)<br>Disagree: 0/13 (0.0%)<br>Neutral: 2/13 (15.4%)<br>Agree: 4/13 (30.8%)<br>Strongly Agree: 6/13 (46.1%)	Strongly Disagree: 0/13 (0.0%)<br>Disagree: 0/13 (0.0%)<br>Neutral: 1/13 (7.7%)<br>Agree: 5/13 (38.5%)<br>Strongly Agree: 7/13 (53.8%)

**Score Distribution Plot**	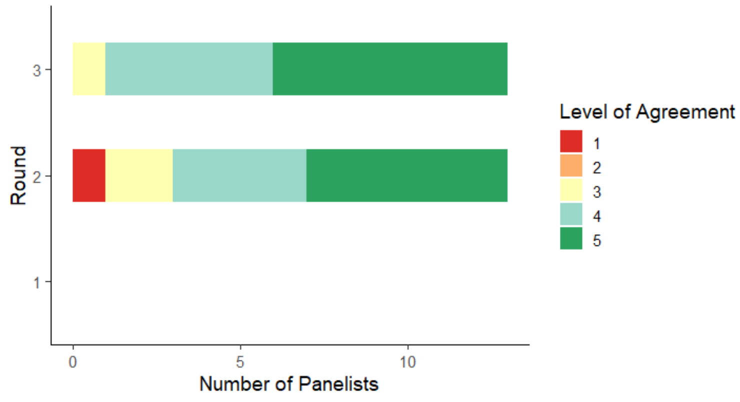

Attribute Disclosure

The Scope of Attribute Disclosure Vulnerability (S7)

Table S8. Statement S7

	Round 1	Round 2	Round 3 (Final Round)
**Statement**	S7: Meaningful attribute disclosure vulnerability only applies to individuals that are in the dataset (i.e., members). Penalizing accurate prediction on individuals that have not been part of the dataset (i.e., group privacy) requires a broader ethical framework.	S7: Meaningful attribute disclosure vulnerability only applies to individuals that are in the dataset (i.e., members). Penalizing accurate prediction on individuals that have not been part of the dataset (i.e., group privacy) requires a broader ethical framework.	S7: Meaningful attribute disclosure vulnerability only applies to individuals that are in the dataset (i.e., members). Penalizing accurate prediction on individuals that have not been part of the dataset (i.e., group privacy) requires a broader ethical framework.
**Explanation**	E7: In technical privacy research an increasingly accepted principle is to make sure that being a member in a dataset does not increase the likelihood of an adversary gaining sensitive information about an individual so that everything that can be learned about the individual can also be learned without them being a member of the dataset. From this perspective, it follows that inferences about non-members cannot be attribute disclosure in the sense of a privacy compromise. This would be more consistent with the goals of research where inference about non-members results from publishing aggregate statistics and prognostic results.	E7: In technical privacy research an increasingly accepted principle is to make sure that being a member in a dataset does not increase the likelihood of an adversary gaining sensitive information about an individual so that everything that can be learned about the individual can also be learned without them being a member of the dataset. From this perspective, it follows that inferences about non-members cannot be attribute disclosure in the sense of a privacy compromise. This would be more consistent with the goals of research where inference about non-members results from publishing aggregate statistics and prognostic results.	E7: In technical privacy research an increasingly accepted principle is to make sure that being a member in a dataset does not increase the likelihood of an adversary gaining sensitive information about an individual so that everything that can be learned about the individual can also be learned without them being a member of the dataset. From this perspective, it follows that inferences about non-members cannot be attribute disclosure in the sense of a privacy compromise. This would be more consistent with the goals of research where inference about non-members results from publishing aggregate statistics and prognostic results.
**Score**	Strongly Disagree: 0/13 (0.0%)	Strongly Disagree: 0/13 (0.0%)	Strongly Disagree: 0/13 (0.0%)

	Disagree: 2/13 (15.4%)<br>Neutral: 0/13 (0.0%)<br>Agree: 4/13 (30.8%)<br>Strongly Agree: 7/13 (53.8%)	Disagree: 2/13 (15.4%)<br>Neutral: 0/13 (0.0%)<br>Agree: 3/13 (23.1%)<br>Strongly Agree: 8/13 (61.5%)	**Disagree: 1/13 (7.7%)**<br>**Neutral: 1/13 (7.7%)**<br>Agree: 1/13 (7.7%)<br>Strongly Agree: 10/13 (76.9%)
**Qualitative Analysis**	**Qualitative Analysis**<br>While achieving broad agreement among the panelists (11/13, 84.6%), this statement also resulted in 1/13 (7.7%) rating on disagreement and 1/13 (7.7%) on uncertainty. There were no counterarguments in the key topics identified regarding this statement. However, there are opinions in the literature that would support a different conclusion. A legal analysis, for example, describes that inferring information about non-members could indeed be interpreted as a privacy violation [4]. At the same time, however, the authors acknowledge that in the United States (US) this would probably raise First Amendment challenges. The First Amendment in the US protects freedom of speech and inference constitutes speech. Similarly, it has been proposed that the concept of privacy can be extended to groups or to collective privacy in the context of big data [5,6]. The remaining disagreement may be in line with these opinions, may be opposed to the idea of a broader ethical framework or result from misunderstanding and/or ambiguity in the statement.		
**Score Distribution Plot**	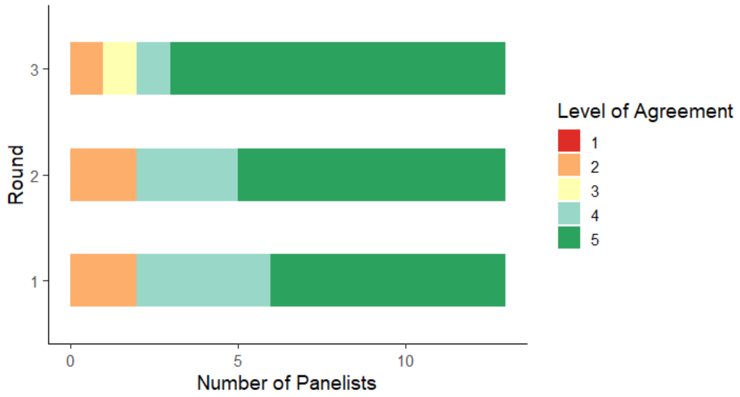		

Knowledge Generation: A Non-Member Baseline (S8)

Table S9. Statement S8.

	**Round 1**	**Round 2**	**Round 3 (Final Round)**
**Statement**	S8: A relative attribute disclosure vulnerability that takes a non-member baseline into account is meaningful.	S8: A relative attribute disclosure vulnerability that takes a non-member baseline into account is meaningful.	S8: A relative attribute disclosure vulnerability that takes a non-member baseline into account is meaningful.
**Explanation**	E8: A baseline derived from the prediction accuracy experienced by non-members distinguishes attribute disclosure vulnerability from knowledge generation. In technical privacy research an increasingly accepted principle is to make sure that being a member in a dataset does not materially increase the likelihood of an adversary gaining sensitive information about an individual so that everything that can be learned about the individual can also be learned without them being a member of the dataset. From this perspective, it follows that inferences about non-members cannot be attribute disclosure in the sense of a privacy compromise.	E8: A baseline derived from the prediction accuracy experienced by non-members distinguishes attribute disclosure vulnerability from knowledge generation. In technical privacy research an increasingly accepted principle is to make sure that being a member in a dataset does not materially increase the likelihood of an adversary gaining sensitive information about an individual so that everything that can be learned about the individual can also be learned without them being a member of the dataset. From this perspective, it follows that inferences about non-members cannot be attribute disclosure in the sense of a privacy compromise.	E8: A baseline derived from the prediction accuracy experienced by non-members distinguishes attribute disclosure vulnerability from knowledge generation. In technical privacy research an increasingly accepted principle is to make sure that being a member in a dataset does not materially increase the likelihood of an adversary gaining sensitive information about an individual so that everything that can be learned about the individual can also be learned without them being a member of the dataset. From this perspective, it follows that inferences about non-members cannot be attribute disclosure in the sense of a privacy compromise.
**Score Distribution**	Strongly Disagree: 0/13 (0.0%)<br>Disagree: 0/13 (0.0%)<br>Neutral: 0/13 (0.0%)<br>Agree: 6/13 (46.1%)<br>Strongly Agree: 7/13 (53.8%)	Strongly Disagree: 0/13 (0.0%)<br>Disagree: 0/13 (0.0%)<br>Neutral: 0/13 (0.0%)<br>Agree: 5/13 (38.5%)<br>Strongly Agree: 8/13 (61.5%)	Strongly Disagree: 0/13 (0.0%)<br>Disagree: 0/13 (0.0%)<br>Neutral: 0/13 (0.0%)<br>Agree: 3/13 (23.1%)<br>Strongly Agree: 10/13 (76.9%)

**Score Distribution Plot**	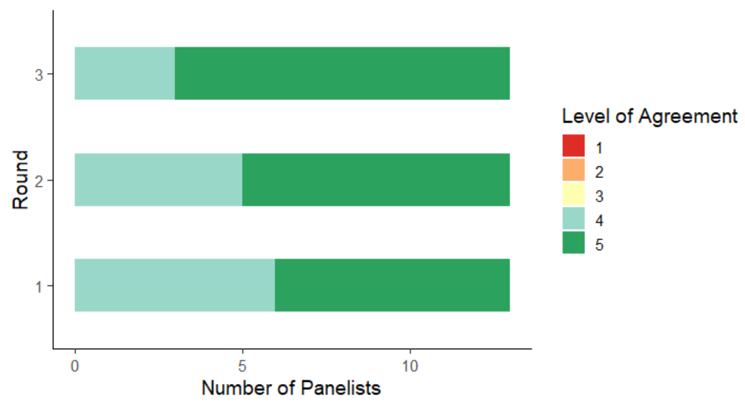

Interpretation Beyond Random Guessing (S9)

Table S10. Statement S9

	**Round 1**	**Round 2**	**Round 3 (Final Round)**
**Statement**	S9: In attribute disclosure vulnerability, a relative vulnerability higher than its threshold is only considered as unacceptably high when the absolute vulnerability is higher than its threshold.	S9: In attribute disclosure vulnerability, a relative vulnerability higher than its threshold is only considered as unacceptably high when the absolute vulnerability is higher than its threshold.	S9: In attribute disclosure vulnerability, a relative vulnerability higher than its threshold is only considered as unacceptably high when the absolute vulnerability is higher than its threshold.
**Explanation**	E9: The relative metric cannot be interpreted uniformly across the entire scale and is probably not always informative by itself. Therefore the decision rule for acceptable attribute disclosure needs to also consider the absolute vulnerability value.	E9: The relative metric cannot be interpreted uniformly across the entire scale and is probably not always informative by itself. Therefore the decision rule for acceptable attribute disclosure needs to also consider the absolute vulnerability value.	E9: The relative metric cannot be interpreted uniformly across the entire scale and is probably not always informative by itself. Therefore the decision rule for acceptable attribute disclosure needs to also consider the absolute vulnerability value.
**Score Distribution**	Strongly Disagree: 1/13 (7.7%)<br>Disagree: 0/13 (0.0%)<br>Neutral: 2/13 (15.4%)<br>Agree: 9/13 (69.2%)<br>Strongly Agree: 1/13 (7.7%)	Strongly Disagree: 1/13 (7.7%)<br>Disagree: 0/13 (0.0%)<br>Neutral: 1/13 (7.7%)<br>Agree: 9/13 (69.2%)<br>Strongly Agree: 2/13 (15.4%)	Strongly Disagree: 0/13 (0.0%)<br>**Disagree: 1/13 (7.7%)**<br>Neutral: 0/13 (0.0%)<br>Agree: 10/13 (76.9%)<br>Strongly Agree: 2/13 (15.4%)

**Qualitative Analysis**	Among our experts, there was 1/13 (7.7%) who disagreed with this statement. Counterarguments in key topics related to this statement did not question the relevance of the absolute scale but rather the application of a threshold in this context or the use of two metrics instead of a proportional metric as in [7]. The discussion on thresholds in general is presented below in the main manuscript. A proportional metric as in [7] is defined relative to the maximum possible attribute disclosure vulnerability beyond the non-member baseline: $$R = \frac{r_{train} - r_{control}}{1 - r_{control}} \quad (1)$$ where r_{train} is the prediction accuracy for members and $r_{control}$ the one for non-members. The idea is that this metric accounts for both absolute and relative scale. It treats differences at the upper end of the scale as more severe than those at the lower end. At the same time, however, it penalizes high non-member baselines and thereby penalizes potentially relevant population-level information (i.e., knowledge generation). Assuming, for example, a non-member baseline of 0.9 accuracy. This is commonly seen as a very good or excellent model [8]. The high accuracy on unseen data (i.e., non-members) suggests that relevant and externally valid population-level information have been derived from the data. At this end of the scale, however, a slightly higher member accuracy of 0.95 would result in a high proportional vulnerability estimate of 0.5. Using a threshold of 0.2 which has been used for such ratios elsewhere [9–11], this would be considered as an unacceptably high vulnerability. A proportional metric thus comes with the challenge that potentially highly valuable insights would be considered as disclosure even though the actual difference is negligible.
**Score Distribution Plot**	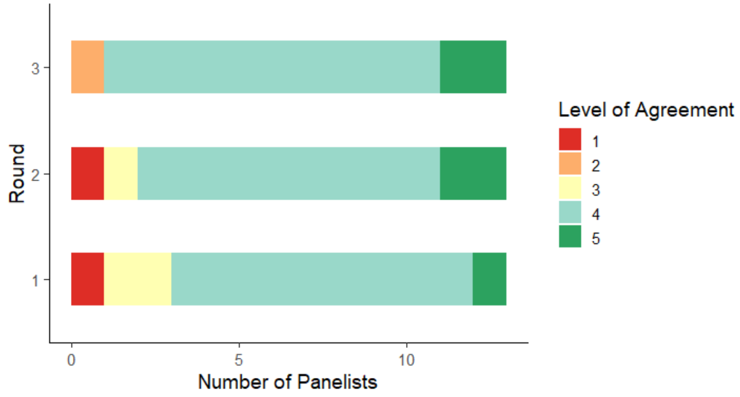

Differential Privacy (S10 & S17)

Table S11. Statement S10

	Round 1	Round 2	Round 3 (Final Round)
**Statement**	S10: Non-differentially private SDG models should be preferred over current applications of Differential Privacy in SDG.	**S10:** omitted	NA
**Explanation**	E10: The value of the privacy budget epsilon has no uniform translation into privacy and can only be interpreted contextually. The consequences of the privacy budget are not the same across different statistical analyses and its value cannot be easily translated into empirical privacy. Differentially private synthetic data would therefore still require a privacy evaluation. Current implementations cannot provide a reasonable privacy-utility trade-off.	NA	NA
**Commentary**	The statement was developed based on the initial report.	The statement was removed since it assessed the application of DP to SDG instead of focusing on epsilon as a privacy metric.	NA
**Score Distribution**	Strongly Disagree: 3/13 (23.1%)<br>Disagree: 1/13 (7.7%)<br>Neutral: 7/13 (53.8%)<br>Agree: 1/13 (7.7%)<br>Strongly Agree: 1/13 (7.7%)	NA	NA

Score Distribution Plot	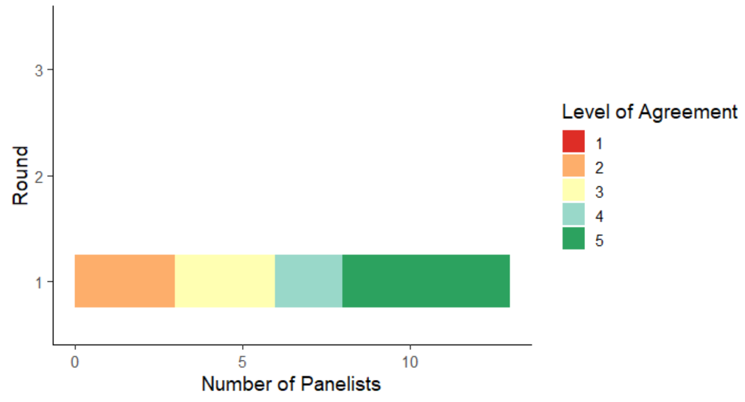

Table S12. Statement S17

	Round 1	Round 2	Round 3 (Final Round)
**Statement**	NA	**S17**: The privacy budget epsilon is not an adequate metric to report disclosure vulnerability. Disclosure vulnerability would still need to be evaluated using the same metrics as those applied to non-differentially private synthetic data.	**S17.1**: The privacy budget epsilon is not an adequate metric to report disclosure vulnerability unless it is set to a value close to 0. Even when differential privacy methods are used, disclosure vulnerability would still need to be evaluated using the same metrics as those applied to non-differentially private synthetic data.
**Explanation**	NA	**E17**: The value of the privacy budget epsilon has no uniform translation into empirical privacy and can only be interpreted contextually. This is in particular true when the privacy budget is relatively large. Differentially private synthetic data would therefore still require a full privacy evaluation.	E17: The value of the privacy budget epsilon has no uniform translation into empirical privacy and can only be interpreted contextually. This is in particular true when the privacy budget is relatively large. Differentially private synthetic data would therefore still require a full privacy evaluation.
**Commentary**	NA	The statement was introduced to focus on the privacy evaluation of DP-SDG, rather than on utility or implementation challenges.	The rephrasing in the statement did not change the recommendation but increased readability.
**Score Distribution**	NA	Strongly Disagree: 1/13 (7.7%)<br>Disagree: 1/13 (7.7%)<br>Neutral: 1/13 (7.7%)<br>Agree: 4/13 (30.8%)<br>Strongly Agree: 6/13 (46.1%)	Strongly Disagree: 0/13 (0.0%)<br>Disagree: 0/13 (0.0%)<br>Neutral: 2/13 (15.4%)<br>Agree: 2/13 (15.4%)<br>Strongly Agree: 9/13 (69.2%)

Score Distribution Plot

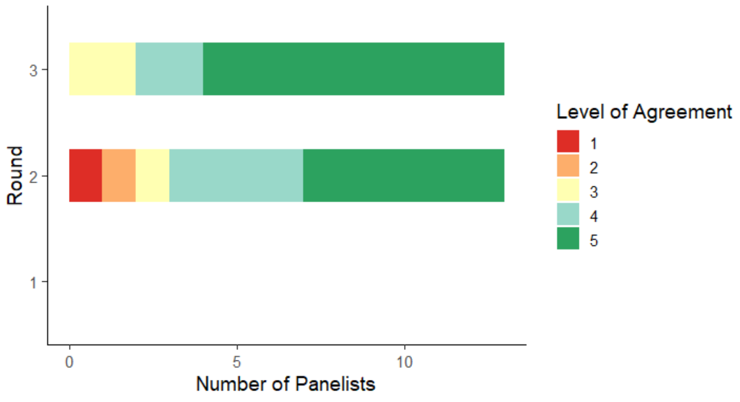

Metric Interpretation and Decision-Making

Stochasticity of the Process (S11)

Table S13. Statement S11

	**Round 1**	**Round 2**	**Round 3 (Final Round)**
**Statement**	S11: When evaluating an SDG model, disclosure vulnerability metrics need to be reported for multiple (e.g. averaged) synthetic datasets.	**S11.1:** When evaluating a specific trained SDG model, disclosure vulnerability metrics need to be reported both for individual and multiple synthetic datasets (e.g. averaged across them and variation).	S11.1: When evaluating a specific trained SDG model, disclosure vulnerability metrics need to be reported both for individual and multiple synthetic datasets (e.g. averaged across them and variation).
**Explanation**	E11: SDG is a generative process with stochastic variability in its output. When evaluating an SDG model rather than a single synthetic dataset, an aggregate across multiple synthetic datasets from the same model would be appropriate.	**E11.1**: SDG is a generative process with stochastic variability in its output. When evaluating an SDG model rather than a single synthetic dataset, an aggregate (average and standard deviation) across multiple synthetic datasets from the same model would be appropriate. This would then reflect the vulnerability of an SDG model tied to a training dataset. However, if the decision-making scenario is data release, then the disclosure vulnerability for the specific synthetic dataset(s) may be the most relevant. Given that it is not always possible to determine a priori the exact decision-making scenario, it would be prudent to have both types of results.	E11.1: SDG is a generative process with stochastic variability in its output. When evaluating an SDG model rather than a single synthetic dataset, an aggregate (average and standard deviation) across multiple synthetic datasets from the same model would be appropriate. This would then reflect the vulnerability of an SDG model tied to a training dataset. However, if the decision-making scenario is data release, then the disclosure vulnerability for the specific synthetic dataset(s) may be the most relevant. Given that it is not always possible to determine a priori the exact decision-making scenario, it would be prudent to have both types of results.
**Commentary**	The statement was developed based on the initial report.	The rephrasing in the statement included the implicit assumptions about other decision-making scenarios. The explanation was rephrased to include	NA

		further details from the report as a rationale for this statement.	
**Score Distribution**	Strongly Disagree: 0/13 (0.0%)<br>Disagree: 2/13 (15.4%)<br>Neutral: 2/13 (15.4%)<br>Agree: 4/13 (30.8%)<br>Strongly Agree: 5/13 (38.5%)	Strongly Disagree: 0/13 (0.0%)<br>Disagree: 0/13 (0.0%)<br>Neutral: 1/13 (7.7%)<br>Agree: 5/13 (38.5%)<br>Strongly Agree: 7/13 (53.8%)	Strongly Disagree: 0/13 (0.0%)<br>Disagree: 0/13 (0.0%)<br>Neutral: 1/13 (7.7%)<br>Agree: 5/13 (38.5%)<br>Strongly Agree: 7/13 (53.8%)
**Score Distribution Plot**	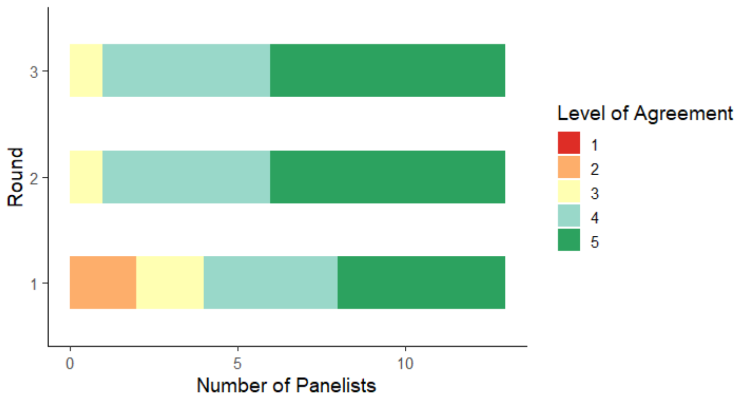		

Thresholds (S12, S13, S14 & S15)

Table S14. Statement S12

	**Round 1**	**Round 2**	**Round 3 (Final Round)**
**Statement**	S12: In membership disclosure vulnerability, an absolute vulnerability (i.e., F1 value) lower than or equal to 0.5 can be considered as acceptably low.	**S12**: omitted	NA
**Explanation**	E12: Membership disclosure vulnerability is a binary classification problem. Accordingly, an accuracy of 0.5 would be a random guess between members and non-members. This threshold applies to both, the naïve (inherent) vulnerability and the absolute vulnerability of the synthetic data.	NA	NA
**Commentary**	NA	The statement was removed and replaced by S16 since it does not hold for *F1 score* which is commonly used to report membership disclosure vulnerability.	NA
**Score Distribution**	Strongly Disagree: 2/13 (15.4%)<br>Disagree: 1/13 (7.7%)<br>Neutral: 3/13 (23.1%)<br>Agree: 5/13 (38.5%)<br>Strongly Agree: 2/13 (15.4%)	NA	NA

Score Distribution Plot	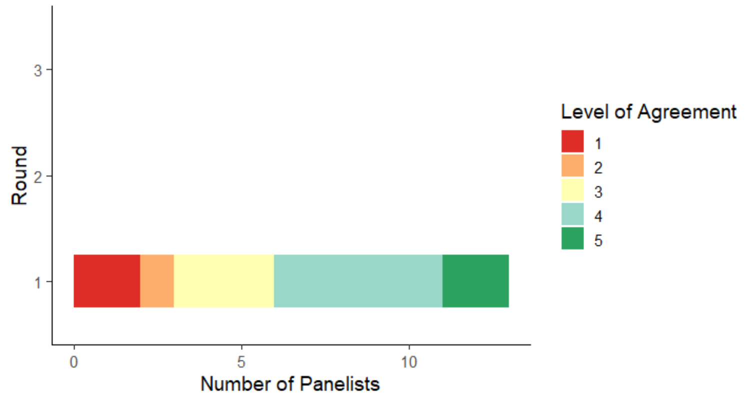

Table S15. Statement S13

	Round 1	Round 2	Round 3 (Final Round)
**Statement**	S13: In membership disclosure vulnerability, a relative vulnerability (i.e., F_rel value) lower than or equal to 0.2 can be considered as acceptably low.	**S13.1**: As a default threshold value for membership disclosure vulnerability, a relative vulnerability (i.e., F_rel value) less than or equal to 0.2 can be considered as acceptably low.	**S13.2**: As an anchor for membership disclosure vulnerability, a relative F1 score vulnerability (i.e., F_rel value) of 0.2 is suggested.
**Explanation**	E13: A common way to define thresholds is to rely on precedents. The threshold that have been used in the literature is 0.2 for relative metrics when compared to a naïve baseline (F_rel).	**E13.1**: The relative metric (F_rel) is a good way to account for the naïve baseline in a non uniformly interpretable F1. This threshold has been used in the literature for the relative membership disclosure metric (F_rel). In practice, thresholds that are recommended based on precedents may be adjusted up or down based on the context. Such adjustments need to be considered carefully and be justified. This context is characterized by the sensitivity of the data, potential harm and appropriateness of consent and notice.	**E13.2**: The relative metric (F_rel) is a good way to account for a naïve baseline to interpret F1. This threshold has been used in the literature for the relative membership disclosure metric (F_rel). In practice, thresholds that are recommended based on precedents may be adjusted up or down based on the context. Such adjustments need to be considered carefully and be justified. This context is characterized by the sensitivity of the data, potential harm and appropriateness of consent and notice.
**Commentary**	NA	The rephrasing in the statement did not change the recommendation but incorporated parts of the explanation. The explanation was rephrased to include further details from the report as a rationale for this statement.	The rephrasing in the statement did not change the recommendation but clarified the meaning of a default threshold / anchor value. The explanation was rephrased to increase readability.
**Score Distributio**	Strongly Disagree: 2/13 (15.4%)<br>Disagree: 0/13 (0.0%)<br>Neutral: 6/13 (46.1%)	Strongly Disagree: 3/13 (23.1%)<br>Disagree: 0/13 (0.0%)<br>Neutral: 7/13 (53.8%)	Strongly Disagree: 1/13 (7.7%)<br>Disagree: 1/13 (7.7%)<br>Neutral: 7/13 (53.8%)

	Agree: 4/13 (30.8%)<br>Strongly Agree: 1/13 (7.7%)	Agree: 2/13 (15.4%)<br>Strongly Agree: 1/13 (7.7%)	Agree: 2/13 (15.4%)<br>Strongly Agree: 2/13 (15.4%)
**Qualitative Analysis**	The panel's ratings reflected a consensus on uncertainty regarding this statement. In the qualitative analysis, two key topics could be identified: First, any threshold, anchor or default value for disclosure vulnerability is considered as inadequate. One reason that was given for this perspective is the contextual interpretation of privacy vulnerability as described above. Another reason is that vulnerability can be reported very differently, for example, based on an average value as well as a maximum value. A concern is that when giving an anchor value people may rely on this value without considering relevant aspects that may trigger adjustments to the value. And second, there is not enough evidence for the very anchor value proposed. Implications of this result are discussed below.		
**Score Distribution Plot**	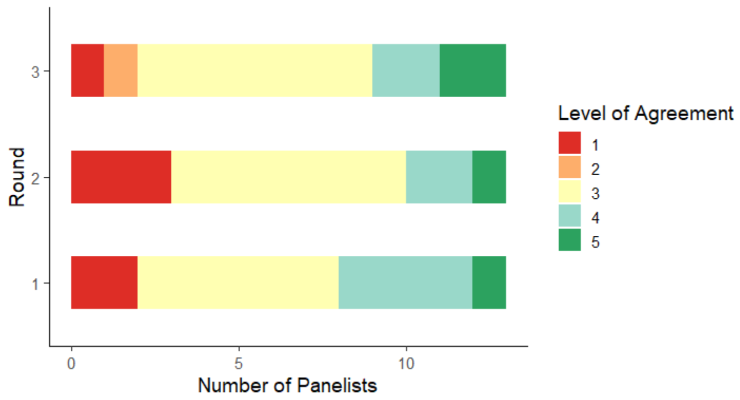		

Table S16. Statement S14

	Round 1	Round 2	Round 3 (Final Round)
**Statement**	S14: In attribute disclosure vulnerability, a distance to the non-member baseline lower than or equal to 0.15 can be considered as acceptably low.	**S14.1**: As a default threshold value for attribute disclosure vulnerability, a difference from the non-member baseline less than or equal to 0.15 can be considered as acceptably low.	**S14.1**: omitted
**Explanation**	E14: For attribute disclosure vulnerability, thresholds could be based on common interpretations of prediction accuracy. Across different labeling systems, labels such as poor, moderate or good prediction accuracy typically change with differences of 0.1 to 0.2 in AUC. A threshold of 0.15 therefore appears reasonable. The threshold would apply to the difference in AUC between member and non-member accuracy, not to a proportional metric (such as a Kappa-like metrics that are relative to the maximum possible attribute disclosure vulnerability beyond knowledge generation).	**E14.1**: For attribute disclosure vulnerability, thresholds could be based on common interpretations of prediction accuracy. Across different labeling systems, labels such as poor, moderate or good prediction accuracy typically change with differences of 0.1 to 0.2 in AUC. A threshold of 0.15 therefore appears reasonable. The threshold would apply to the difference in AUC between member and non-member accuracy. In practice, thresholds that are recommended based on precedents may be adjusted up or down based on the context. Such adjustments need to be considered carefully and be justified. This context is characterized by the sensitivity of the data, potential harm and appropriateness of consent and notice.	NA
**Commentary**	NA	The rephrasing in the statement did not change the recommendation but incorporated parts of the explanation. The explanation was rephrased to include further details from the report as a rationale for this statement.	The statement was removed since it was overly speculative drawing from evidence that was not directly related to privacy.

**Score Distribution**	Strongly Disagree: 1/13 (7.7%)<br>Disagree: 2/13 (15.4%)<br>Neutral: 6/13 (46.1%)<br>Agree: 2/13 (15.4%)<br>Strongly Agree: 2/13 (15.4%)	Strongly Disagree: 3/13 (23.1%)<br>Disagree: 1/13 (7.7%)<br>Neutral: 7/13 (53.8%)<br>Agree: 1/13 (7.7%)<br>Strongly Agree: 1/13 (7.7%)	NA
**Score Distribution Plot**	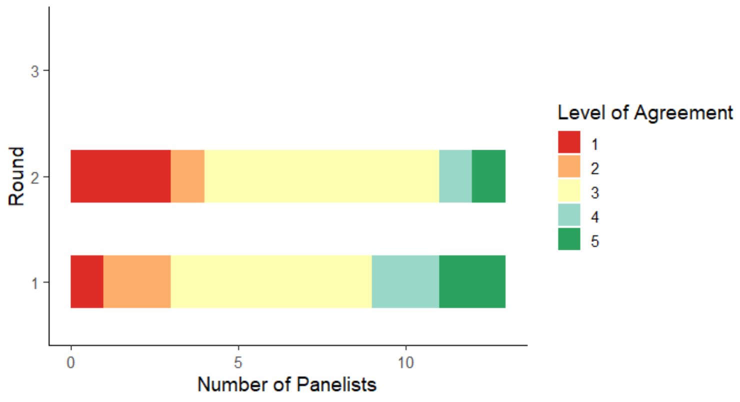 		

Table S17. Statement S15

	Round 1	Round 2	Round 3 (Final Round)
**Statement**	S15: In attribute disclosure vulnerability, an absolute vulnerability (i.e., accuracy of a member) lower than or equal to 0.6 can be considered as acceptably low.	**S15.1**: As a default threshold value for attribute disclosure vulnerability, an absolute vulnerability (i.e., accuracy of predicting a member sensitive attribute) less than or equal to 0.6 can be considered as acceptably low.	**S15.1**: omitted
**Explanation**	E15: Across different labeling systems, poor accuracy is mainly assigned to an AUC lower than or equal to 0.6. Following this interpretation, a threshold of 0.6 could be established for absolute vulnerability, meaning that only accuracies that exceed poor prediction (i.e., 0.6) can be meaningful disclosure.	**E15.1**: Across different labeling systems, poor accuracy is mainly assigned to an AUC lower than or equal to 0.6. Following this interpretation, a threshold of 0.6 could be established for absolute vulnerability, meaning that only accuracies that exceed poor prediction (i.e., 0.6) can be meaningful disclosure. In practice, thresholds that are recommended based on precedents may be adjusted up or down based on the context. Such adjustments need to be considered carefully and be justified. This context is characterized by the sensitivity of the data, potential harm and appropriateness of consent and notice.	NA
**Commentary**	NA	The rephrasing in the statement did not change the recommendation but incorporated parts of the explanation. The explanation was rephrased to include further details from the report as a rationale for this statement.	The statement was removed since it was overly speculative drawing from evidence that was not directly related to privacy.

**Score Distribution**	Strongly Disagree: 1/13 (7.7%)<br>Disagree: 2/13 (15.4%)<br>Neutral: 4/13 (30.8%)<br>Agree: 4/13 (30.8%)<br>Strongly Agree: 2/13 (15.4%)	Strongly Disagree: 2/13 (15.4%)<br>Disagree: 1/13 (7.7%)<br>Neutral: 3/13 (23.1%)<br>Agree: 4/13 (30.8%)<br>Strongly Agree: 3/13 (23.1%)	NA
**Score Distribution Plot**	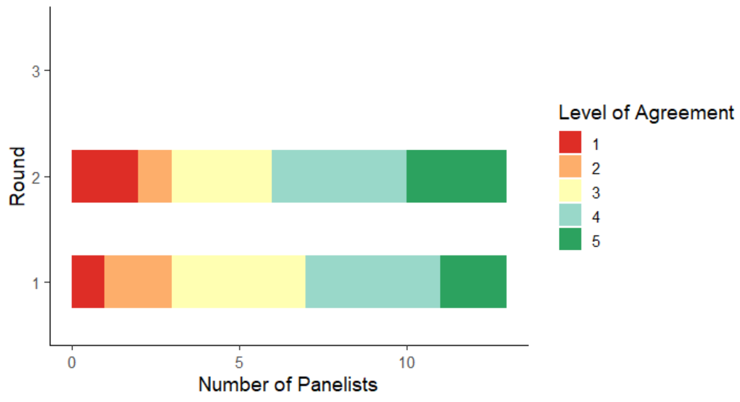		

Supplemental References

1. Pilgram, L., Dankar, F.K., Drechsler, J., Elliot, M., Domingo-Ferrer, J., Francis, P., Kantarcioglu, M., Malin, B., Muralidhar, K., Myles, P., et al. (2025). A Consensus Privacy Metrics Framework for Synthetic Data - Critical Analysis of Privacy Metrics (Report). OSF, https://osf.io/vz5x9.

2. Khodyakov, D., Grant, S., Kroger, J., and Bauman, M. (2023). RAND Methodological Guidance for Conducting and Critically Appraising Delphi Panels (RAND Corporation). https://www.rand.org/pubs/tools/TLA3082-1.html.

3. Malterud, K. (2001). Qualitative research: standards, challenges, and guidelines. Lancet *358*, 483–488. https://doi.org/10.1016/S0140-6736(01)05627-6.

4. Gal, M., and Lynskey, O. (2023). Synthetic Data: Legal Implications of the Data-Generation Revolution. 109 Iowa Law Review, Forthcoming, https://doi.org/10.2139/ssrn.4414385.

5. Mühlhoff, R. (2021). Predictive privacy: towards an applied ethics of data analytics. Ethics Inf Technol *23*, 675–690. https://doi.org/10.1007/s10676-021-09606-x.

6. Mantelero, A. (2017). From Group Privacy to Collective Privacy: Towards a New Dimension of Privacy and Data Protection in the Big Data Era. In Group Privacy: New Challenges of Data Technologies, L. Taylor, L. Floridi, and B. van der Sloot, eds. (Springer International Publishing), pp. 139–158. https://doi.org/10.1007/978-3-319-46608-8_8.

7. Giomi, M., Boenisch, F., Wehmeyer, C., and Tasnádi, B. (2023). A Unified Framework for Quantifying Privacy Risk in Synthetic Data. Preprint at arXiv, https://doi.org/10.48550/arXiv.2211.10459.

8. Hond, A.A.H. de, Steyerberg, E.W., and Calster, B. van (2022). Interpreting area under the receiver operating characteristic curve. The Lancet Digital Health *4*, e853–e855. https://doi.org/10.1016/S2589-7500(22)00188-1.

9. Mendelevitch, O., and Lesh, M.D. (2021). Fidelity and Privacy of Synthetic Medical Data. Preprint at arXiv, 2101.08658 [cs].

10. El Emam, K., Mosquera, L., and Fang, X. (2022). Validating A Membership Disclosure Metric For Synthetic Health Data. JAMIA Open *5*, ooac083. https://doi.org/10.1093/jamiaopen/ooac083.

11. El Kababji, S., Mitsakakis, N., Fang, X., Beltran-Bless, A.-A., Pond, G., Vandermeer, L., Radhakrishnan, D., Mosquera, L., Paterson, A., Shepherd, L., et al. (2023). Evaluating the Utility and Privacy of Synthetic Breast Cancer Clinical Trial Data Sets. JCO Clin Cancer Inform, e2300116. https://doi.org/10.1200/CCI.23.00116.
